# The relationship between college students’ learning engagement and academic self-efficacy: a moderated mediation model

**DOI:** 10.3389/fpsyg.2024.1425172

**Published:** 2024-09-03

**Authors:** Yaxing Wang, Wen Zhang

**Affiliations:** ^1^Mental Health Service Center, Huanghuai University, Zhumadian, China; ^2^School of Psychology, Northwest Normal University, Lanzhou, China; ^3^Engineering Training Center, Huanghuai University, Zhumadian, China

**Keywords:** learning engagement, academic self-efficacy, professional commitment, psychological resilience, college students

## Abstract

**Introduction:**

Despite the return of college students to campus in the post-pandemic era, the deep influence of COVID-19 on learning approaches persists. Existing research has explored fewer mechanisms underlying academic self-efficacy and learning engagement. In line with social cognitive theory, the psychological resilience framework, and vocational socialization theory, this research investigated academic self-efficacy, professional commitment, psychological resilience, and academic engagement among college students in the post-pandemic era. In this research, the focus was on understanding the impact of academic self-efficacy on learning engagement, taking into account gender as a moderator and psychological resilience and professional commitment as mediators.

**Methods:**

We conducted a survey with 1,032 college students in Henan Province, China, utilizing the Psychological Resilience Scale, Academic Self-Efficacy Scale, College Student Learning Engagement Questionnaire, and College Student Professional Commitment Scale. SPSS and the Process plugin were used to assess mediating and moderating effects.

**Results:**

Academic self-efficacy significantly and positively correlates with college students’ commitment to learning. The positive anticipation of learning engagement facilitated by academic self-efficacy exerts its effect through the fully parallel mediation of psychological resilience and professional commitment. Notably, the mediation effect of professional commitment was greater than that of psychological resilience. Further research found that the mediation of professional commitment was moderated by gender, with female students demonstrating stronger perceptions of professional commitment associated with elevated levels of learning engagement. Gender did not exhibit a significant moderating effect on psychological resilience.

**Conclusion:**

College students’ academic self-efficacy, professional commitment, and psychological resilience must be addressed to enhance their learning engagement.

## 1 Introduction

Educators are concerned with the level of learners’ engagement (Zheng, 2023). In the post-pandemic era, effective implementation of measures to enhance learning engagement among college students is a concern for many countries. Research suggests that effective learning hinges on learners themselves (Kumar and Todd, 2022). Effective learning necessitates students’ active participation, the internalization of acquired knowledge, and the formation of their own learning experiences (Rashid and Asghar, 2016). Learning engagement is a crucial factor that influences students’ academic performance (Sahni, 2023). Increasingly, countries are associating the extent of learning engagement with academic performance, reward and punishment systems, as well as dropout and graduation rates.

Learning engagement serves as a crucial predictor of the quality of learning (Bayoumy and Alsayed, 2021). During the pandemic, college students predominantly engaged in home-based learning over the Internet. The learning mode changed significantly from the pre-pandemic period, transitioning from traditional in-person group learning to solo distance learning. After the pandemic, students returned to the classroom for in-person learning; however, they demonstrated low learning initiative and diminished levels of learning engagement (Yang et al., 2022, Salmela-Aro et al., 2023)–perhaps due to the effects of learning modes used during the pandemic. Indeed, studies show that prolonged remote learning has resulted in students demonstrating lower levels of interaction and autonomous learning, ultimately impacting their participation in traditional classroom settings (Järvelä et al., 2022, Kahu et al., 2022). Most existing studies on learning engagement have focused on its current situation, characteristics, and influencing factors (Wang et al., 2020, Pelikan et al., 2021). Notably, academic self-efficacy, an important concept in social cognitive theory, is a significant factor influencing students’ learning behaviors and motivation. However, little research has been done in the post-pandemic era on the impact of academic self-efficacy on student learning engagement. In response to this gap in the literature, this study sought to investigate the reasons behind reduced learning initiative and engagement among college students post-pandemic and to examine the intrinsic link between academic self-efficacy and learning engagement. The findings stand to provide new ideas on how to improve college students’ learning engagement in the post-pandemic era.

Learning engagement refers to the degree of time and effort students invest in the learning process, resulting in sustained and enriched affective and cognitive states (Schaufeli et al., 2002; Fredricks et al., 2004). Various theories elucidate the learning engagement process, such as social cognitive and self-determination theories. Related to this paper is social cognitive theory, which posits that individual behavior can be influenced by social environmental and personal factors (Ryan and Deci, 2020; Schunk and DiBenedetto, 2021).

Importantly, social cognitive theory led to the concept of self-efficacy (Bandura, 2021), pertains to an individual’s confidence and feelings regarding their organization and execution of a specific task (Bandura, 1986; Bandura, 1997). First introduced by Bandura, self-efficacy is an important predictor of learning and strongly influences behavior and performance. Self-efficacy is a comprehensive concept and can be broken down into two components: efficacy and outcome expectations (You, 2022). Since its introduction, diverse fields have undertaken extensive research on the topic, resulting in the development of derivative concepts, such as academic and organizational self-efficacy. Bandura believed that self-efficacy is affected by the environment in which it is situated. On the one hand, it affects cognitive processes, with high self-efficacy fostering individual cognitive development, thereby enhancing academic behavior. On the other hand, it influences individual behavior (Bandura, 2012). People with strong self-efficacy select difficult academic projects and work hard to complete them. Moreover, when they encounter significant setbacks, they recover swiftly and pursue their goals. Prior research has substantiated social cognitive theory and identified a close relationship between learning engagement, psychological resilience (Smith et al., 2008; Hartley, 2011; Zeng et al., 2016; Zhao et al., 2021), and perceived learning ineffectiveness (Ye et al., 2023).

Academic self-efficacy involves learners’ self-assessment of their learning abilities. Learners exhibit confidence and a sense of competence in organizing and executing specific learning tasks, leading to a successful understanding of learning materials (Bandura, 1997). As a significant predictive factor in learning, it profoundly influences students’ learning behavior and performance (Maslow, 1987). Here, it is useful to recall that the pandemic might have influenced students’ societal requirements and feelings of safety, potentially affecting their eagerness to learn (Gagne and Deci, 2020). Further, numerous investigations have shown a positive association between college students’ academic self-efficacy and learning engagement (Allari et al., 2020). Individuals with robust academic self-efficacy showed heightened confidence in completing learning tasks and demonstrated elevated levels of engagement in their studies. Conversely, students with lower academic self-efficacy may experience heightened feelings of helplessness, encounter increased negative emotions, and exhibit reduced participation in their studies (Namaziandost et al., 2023). Academic self-efficacy motivates learners to adopt methods that align with their goals, thereby exerting a substantial influence on the completion of learning tasks. Individuals with robust academic self-efficacy possess a solid cognitive understanding of the learning process and attribute a lack of success to insufficient effort rather than a lack of ability. Significant associations have been observed between students’ academic self-efficacy and their levels of learning engagement (Xie and Xie, 2019).

### 1.1 Mediating effect of psychological resilience

Psychological resilience denotes an individual’s capacity to bolster their ability to cope with adversities and respond effectively to sources of stress when confronted with challenges (Ahern and Norris, 2011; Cooper et al., 2020). It refers to the capacity to maintain a positive adaptive state or “bounce back” to normal life when facing adversity, trauma, misfortune, or significant stressors (Kumpfer, 2002). The psychological resilience framework posits that individuals generate three adaptive outcomes when dealing with stress: an increase in resilience levels, maintaining the original level of resilience, and a decrease in resilience levels after experiencing the shock of stress. The emergence of various adaptive outcomes are influenced by the environment, individual factors, and individual-environment interactions (Luthar et al., 2000). This theory proposes that psychological resilience is dynamic and malleable, playing a crucial role in safeguarding psychological growth (Cheung et al., 2019). Psychological resilience is not an inherent personality trait; rather, it continuously develops throughout an individual’s entire life course and is influenced by the surrounding living environment (Gillespie et al., 2007; Celik et al., 2015). Leontopoulou (2006) found that both positive and avoidance coping strategies significantly influenced psychological resilience even in adversity. Individuals with robust psychological resilience exhibit strong adaptive capabilities and a high capacity to absorb and utilize coping strategies. Notably, positive emotions play a crucial role in the development of psychological resilience—they not only broaden an individual’s attention and cognition but also support the development of personal positive resources, enhancing behavioral positivity (Chmitorz et al., 2018). Alazemi et al. (2023) revealed that greater academic psychological resilience in students correlates with increased self-efficacy, while lower self-efficacy shows the opposite trend. The discovery that academic self-efficacy reflects self-efficacy in learning further suggests a strong correlation between self-efficacy and psychological resilience.

Social cognitive theory highlights that self-efficacious people possess strong convictions of successfully completing tasks, set challenging goals, and invest energy and perseverance in coping when facing difficulties. In their study of second language learning, Wicaksono et al. (2023) discovered a robust association between self-efficacy, perseverance, academic demotivation, and academic resilience. Self-efficacy and perseverance enable learners to cultivate positive expectations for learning outcomes in the process of second language acquisition, enhance academic resilience, and sustain efficient learning engagement in the long run. Shao and Kang (2022) revealed close relationships among academic psychological resilience, self-efficacy, and learning engagement. Despite encountering challenges, students with academic psychological resilience frequently possess strong confidence in successfully completing learning tasks and believe in their ability to do so. Consequently, they exhibit elevated levels of learning engagement. Through a survey of 155 high school students in India, Rajan et al. (2017) found a significant gender difference in academic resilience, and academic resilience and self-efficacy were positively correlated among high school students. These studies suggest close relationships among psychological resilience, learning engagement, and academic self-efficacy. However, the underlying mechanisms between these three factors still need to be clarified.

### 1.2 Mediating effect of professional commitment

Professional commitment refers to an individual’s attitude and behavior toward their chosen major, indicating their identification with the major and willingness to invest time and effort in the field of study (Lian et al., 2005), and is a manifestation of individuals’ love for and fidelity to their majors. Professional commitment serves as a crucial indicator for comprehending the extent of student engagement in their majors. According to vocational socialization theory, people slowly cultivate a sense of identity and dedication to their profession through their career progression and decision-making processes. The dynamics of these processes are shaped by a mix of individual convictions, societal anticipations, and work environments (Weidman, 1989). Within educational settings, students’ dedications to their fields of study play a crucial role in shaping their professional socialization. Notably, college students exhibiting a strong professional commitment tend to demonstrate greater academic involvement, as their career aspirations and convictions propel them to invest more in their current educational pursuits. Previous research has substantiated a significant association between professional commitment and learning engagement (Lu et al., 2023). With 750 preschool education college students as participants, Chen (2018) investigated their learning satisfaction, professional commitment and learning engagement using a questionnaire method, and found that the participants demonstrated a moderate level of professional commitment while achieving high scores in learning engagement. Students deeply committed to their major exhibit greater academic involvement, as their strong connection to their field motivates them to exert more effort in their academic tasks (Yan et al., 2023).

It has been shown that self-efficacy is closely related to professional commitment, particularly emotional commitment (Lent et al., 2002). Tsai et al. (2014) concluded that a heightened level of self-efficacy positively influences emotional commitment. This beneficial impact arises from individuals with higher self-efficacy being more inclined to align with an organization’s objectives and principles compared to those with lower self-efficacy. Wang and Hsu (2014) indicate that professional commitment mediates the link between self-efficacy and academic achievement. Specifically, their study revealed that students with higher self-efficacy typically exhibit greater commitment to their field of study, which in turn enhances their motivation and engagement in learning, ultimately leading to higher academic achievement. Self-efficacy is an important factor influencing individual behavior and motivation (Bandura, 1997). Individuals with high academic self-efficacy tend to show strong professional commitment and demonstrate greater engagement and perseverance in fulfilling those commitments. Consequently, the research posits that professional commitment may mediate the relationship between academic self-efficacy and professional commitment, highlighting the complex relationship among professional commitment, academic self-efficacy, and learning engagement.

### 1.3 The moderating effect of gender

Gender is a crucial demographic variable affecting learning engagement. Previous studies indicate notable disparities in the learning involvement of college students based on gender (Xie and Shauman, 2003). Gender differentiation theory suggests that due to physiological differences, individuals gradually develop gender role concepts through social construction. As individuals grow and socialize, they slowly assimilate societal norms and expectations for various genders (Skaar et al., 2014). According to social role theory, disparities in behavior and attitude between men and women are often attributed to gendered social expectations and cultural norms (Eagly, 1987). For example, men are commonly expected to demonstrate higher levels of competitiveness and autonomy in their academic and professional pursuits, while women are presumed to play more collaborative and supportive roles (Eagly and Wood, 1999). Such gendered differences may affect professional and educational commitments.

Gender differentiation theory suggests that due to the physiological differentiation of gender, individuals gradually develop gender role concepts in the process of social construction. This process implies the ongoing development of individuals and progression of the socialization process. Individuals of different genders uniquely engage in professional learning, adjusting their expectations of their major based on the understanding formed through learning. The level of professional commitment derived from this process is also diverse, resulting in varying levels of learning engagement (Chen, 2018). Meece et al. (2006) indicate that while women exhibit significant learning engagement with strong professional commitment, they tend to sustain this level of engagement even given lower levels of professional commitment, likely owing to varied learning approaches and motivational factors. More specifically, this may be because women tend to focus more on academic aspects related to processes and personal gratification. Nonetheless, evidence also indicates that men demonstrate greater involvement in learning given strong professional commitments (Eccles, 2009). Notably, men may be influenced by societal norms to participate more actively in learning to attain their career objectives given high professional commitment. Current research on the effects of gender on learning engagement and professional commitment is controversial, highlighting the need for further investigation.

### 1.4 Objective and hypotheses

**Figure 1:**
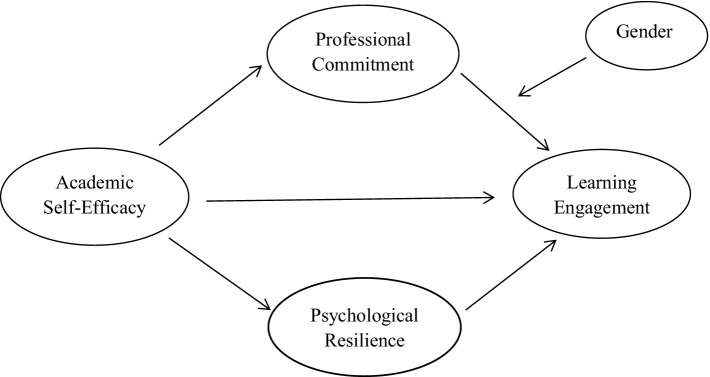
Diagram of the model.

In light of the above, this study integrates various theories and existing research to develop a moderated mediation model (Figure 1). We apply the model to investigate the mediating roles of psychological resilience and professional commitment in the relationship between academic self-efficacy and learning engagement. Additionally, we use the model to examine the indirect effect of gender on the relationship between professional commitment and learning engagement.

Based on the above literature review, the following four hypotheses are proposed:

*H1*: Academic self-efficacy positively predicts learning engagement.

*H2*: Psychological resilience mediates the relationship between academic self-efficacy and learning engagement.

*H3*: Professional commitment mediates the relationship between academic self-efficacy and learning engagement.

*H4*: Gender moderates the relationship between professional commitment and learning engagement.

## 2 Materials and methods

### 2.1 Participants

This study was approved by the Academic Committee of Huanghuai University. Undergraduate students from freshman to senior year at some universities in Henan, China were recruited as participants in the study using whole-cluster random sampling. We employed the anonymous survey platform Wenjuanxing to collect data, garnering a total of 1,187 responses. After excluding incomplete or insincere responses, we obtained 1,032 valid questionnaires. Participants included 479 males (46.4%) and 553 females (53.6%). The participants aged between 18 and 23 years; the average age was 19.63 years old (SD = 1.333).

The participants were enrolled in the following majors: (1) Engineering—this major encompasses classes like Financial Engineering and Software Engineering and focuses on integrating theoretical knowledge with practical application, necessitating student involvement in laboratory activities and designing projects; (2) Information Technology—this major includes courses such as Computer Science, Software Engineering, and Information Systems, which cover topics such as programming, database management, network technology, and system analysis; (3) Medical Studies—this major encompasses subjects like Clinical Medicine, Nursing, and Public Health, which introduce students to basic medical knowledge, clinical applications, and public health management; (4) Broadcasting and Hosting Arts—this major includes courses such as Broadcasting, TV Directing, and Journalism, which covers voice techniques, program hosting, and news writing. Students majoring in these fields transitioned from in-person learning to online learning and subsequently returned to in-person learning. Consequently, the significance of their learning initiative and engagement is crucial to our research goals.

The study was conducted from March to September 2023.hrough the online survey platform Wenjuanxing, with participants collectively tested by class. Before administering the test, the primary examiner described the instructions to the participating students, explained confidentiality, and obtained informed consent from all participants. Participants were assured that participation was anonymous and voluntary, with withdrawal possible in the middle of the test. A small gift was provided to the participants as a token of appreciation for their cooperation. Questionnaires that were carelessly filled out or missing too much data were filtered out. After identifying usable samples, we used the mean imputation method to process the missing values. Questionnaires that were carelessly filled out or missing too much data were filtered out. After identifying usable samples, we used the mean imputation method process the missing values.

### 2.2 Measures

#### 2.2.1 Psychological resilience

This research utilized the Chinese adaptation of the Connor-Davidson Resilience Scale (CD-RISC), modified by academics Yu and Zhang (2007), to evaluate psychological resilience. Developed by American psychologists Connor and Davidson in 2003, the 25-item CD-RISC comprises three dimensions: self-improvement, toughness, and optimism. Utilizing a 5-point scale, participants rate responses from “1 = never” to “5 = almost always.” As scores rise, so does the level of psychological resilience. The Cronbach’s α for CD-RISC was 0.92, and it was 0.96 in this study. The validity of the scale was as follows: *χ*^2^ = 21202.8, df = 300, *p* < 0.001, *χ*^2^/df = 2.97, CFI = 0.98, TLI = 0.98, RMSEA = 0.04.

#### 2.2.2 Academic self-efficacy

The Academic Self-Efficacy Scale developed by Liang (2000) was employed to evaluate academic self-efficacy in the current study. The scale comprises 22 items encompassing two dimensions: self-efficacy for learning ability and self-efficacy for learning behavior. A 5-point scale ranging from “1 = strongly disagree” to “5 = strongly agree” was employed. Questions 14, 16, 17, and 20 were reverse scored, whereas the other items were scored positively. Higher scores on the questionnaire indicate stronger academic self-efficacy. The Cronbach’s α in this study was 0.91. The validity of the scale also improved: *χ*^2^ = 19593.4, df = 231, *p* < 0.001, *χ*^2^/df = 2.977, CFI = 0.98, TLI = 0.97, RMSEA = 0.04.

#### 2.2.3 Learning engagement

The learning engagement questionnaire for college students was employed to assess participants level of learning engagement (Ni, 2020). The questionnaire comprises 20 questions across three dimensions: behavior, cognition, and emotion. A 5-point scale is used, ranging from “1 = not at all compliant” to “5 = fully compliant.” Elevated scores signify increased levels of learning engagement. The Cronbach’s α in this study was 0.96. The validity of the scale was as follows: *χ*^2^ = 19786.3, df = 190, *p* < 0.001, *χ*^2^/df = 2.922, CFI = 0.98, TLI = 0.98, RMSEA = 0.04.

#### 2.2.4 Professional commitment

The College Student Professional Commitment Scale developed by Lian et al. (2005) was used to measure professional commitment. The scale comprises 27 questions organized into dimensions such as affective commitment, continuance commitment, normative commitment, and ideal commitment. A 5-point Likert scale is used to calculate the score, ranging from “1 = not at all” to “5 = completely.” Scores for questions 6, 8, and 12 are reversed-scored. Higher scores indicate a heightened level of professional commitment. The Cronbach’s α in this study was 0.95. The validity of the scale was also good: *χ*^2^ = 22283.2, df = 351, *p* < 0.001, *χ*^2^ /df = 2.969, CFI = 0.97, TLI = 0.96, RMSEA = 0.04.

### 2.3 Statistical analyses

First, SPSS was used to conduct descriptive statistical analyses and Pearson correlation studies. The descriptive statistical analysis, which included measures such as the mean and standard deviation, yielded basic information about college students’ professional commitment, psychological resilience, academic self-efficacy, and learning engagement. Meanwhile, the Pearson correlation coefficient offered a preliminary understanding of the relationships between variables, providing a foundation for the subsequent mediation effect analysis. Next, the study variables were standardized. The independent variable was academic self-efficacy, the dependent variable was learning engagement, and the mediating variables were professional commitment and psychological resilience. SPSS macro PROCESS Model 4 (Hayes, 2015) was used to analyze the parallel mediation effects; this analytical approach can reveal the independent effects and mechanisms of various mediating variables. We used this method to separately explore how professional commitment and psychological resilience influence learning engagement through academic self-efficacy; ultimately, this inquiry clarified the role of these mediating variables within the overall model and their relative importance. Further, bootstrap analyses were performed on 5,000 samples while controlling for gender to estimate the 95% confidence intervals for the mediating effect to confirm the reliability of the mediation effect and thus the robustness of the research findings. Finally, SPSS macro PROCESS Model 14 was employed to analyze the moderated mediating effects to identify the potential role of gender in this relationship and differences between gender groups.

## 3 Results

### 3.1 Common method Bias test

In this research, the gathered data originated from participants’ own accounts, potentially leading to common method bias. Hence, Harman’s one-way test was employed for analysis. The findings revealed 11 factors, each with eigenvalues exceeding 1, accounting for 29.766% of the variance and falling short of the essential threshold of 40% (Podsakoff et al., 2003). Consequently, this research does not exhibit a significant common method bias.

### 3.2 Correlation analysis

The findings in Table 1 reveal a positive link between academic self-efficacy and factors such as psychological resilience, professional commitment, and learning engagement (*r* = 0.340, 0.577, 0.277, respectively; *p* < 0.01). There was also a positive link between psychological resilience and both professional commitment and learning engagement (*r* = 0.352, 0.370, respectively; *p* < 0.01), as well as between professional commitment and learning engagement (*r* = 0.320; *p* < 0.01).

**Table 1:** Descriptive statistics and variable correlation analysis (*n* = 1,032).

Variable	1	2	3	4	5
1. Gender	1				
2. AS	−0.039	1				3. PR	0.015	0.340^**^	1			4. PC	−0.065^*^	0.577^**^	0.352^**^	1	
5. LE	−0.038	0.227^**^	0.370^**^	0.320^**^	1	*M*	1.54	3.42	3.45	3.54	3.65
SD	0.50	0.50	0.64	0.55	0.68

M, mean; SD, standard deviation; AS, academic self-efficacy; PR, psychological resilience; PC, professional commitment; LE, learning engagement.*p < 0.05; **p < 0.01; ***p < 0.001.

### 3.3 Parallel mediation tests

**Figure 2:**
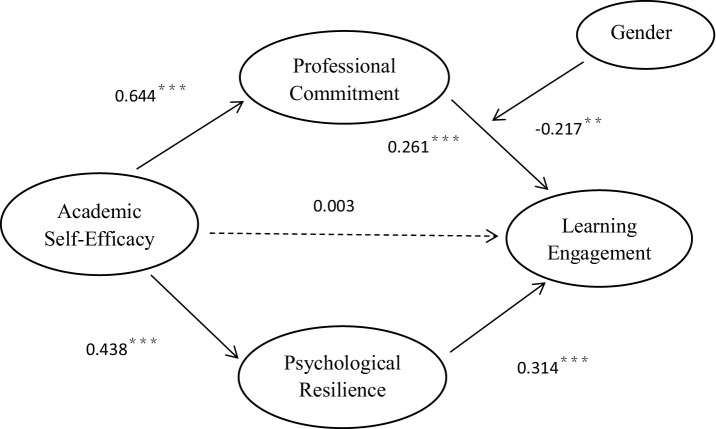
A moderated mediation model.

The present study aimed to investigate the potential mediating roles of psychological resilience and professional commitment in the association between academic self-efficacy and learning engagement. For this analysis, Model 4 of the SPSS macro process, developed by Hayes (2015), was utilized. The findings are displayed in Table 2 and Figure 2. With gender as a control variable, academic self-efficacy positively predicted psychological resilience (*β* = 0.438, *p* < 0.001) and professional commitment (*β* = 0.644, *p* < 0.001). Both psychological resilience and professional commitment can predict positive learning engagement (*β* = 0.314, *p* < 0.001; *β* = 0.261, *p* < 0.001). In contrast, academic self-efficacy became insignificant in predicting learning engagement (*β* = 0.003, *p* = 0.9476). This suggests that academic self-efficacy does not directly influence learning engagement, and that professional commitment and psychological resilience fully mediate the relationship between academic self-efficacy and learning engagement.

**Table 2:** Results with mediation effects.

	Influence path	Effect	95% CI	Relative mediating effect (%)
Indirect effect	PR	0.138	[0.097 0.183]	45.10%
	PC	0.168	[0.105 0.230]	54.9%
Total indirect effect		0.306	[0.235 0.378]	100%

CI, confidence interval; PR, psychological resilience; PC, professional commitment.

Findings from the analysis of mediating effects revealed that the mediating effect value for academic self-efficacy → psychological resilience → learning engagement stood at 0.138, while that of academic self-efficacy → professional commitment → learning engagement was 0.168. The 95% confidence interval for this effect value excluded 0, reflecting the significance of both psychological resilience and professional commitment in the link between academic self-efficacy and learning engagement among college students. Put differently, the link between academic self-efficacy and learning engagement is completely mediated by psychological resilience and professional commitment.

### 3.4 Moderated mediation effects tests

To further explore the reasons for gender differences in professional commitment, Model 14 was used to test gender’s moderating role between the original parallel mediator models. The results are shown in Table 3, with the significance of the original paths is revealed to be consistent with previous observations. Gender exhibited a significant moderating effect on the latter segment of professional commitment mediation (*β* = −0.217, *p* < 0.01), whereas the moderating effects on the initial part of professional commitment and both segments of psychological resilience mediation were not statistically significant.

**Table 3:** Results with moderated mediation effects.

Regression equation	Fit index	Significance of regression coefficient				
Outcome variable	Predictor variable	*R*	*R* ^2^	*F*	*β*	*t*
Learning engagement		0.337	0.113	32.836^***^		
	AS				0.092	1.874
	PC				0.3282	7.384^***^
	Gender				−0.023	−0.582
	Gender × PC				−0.217	−3.000^**^

AS, academic self-efficacy; PC, professional commitment. *p < 0.05; **p < 0.01; ***p < 0.001.

**Figure 3:**
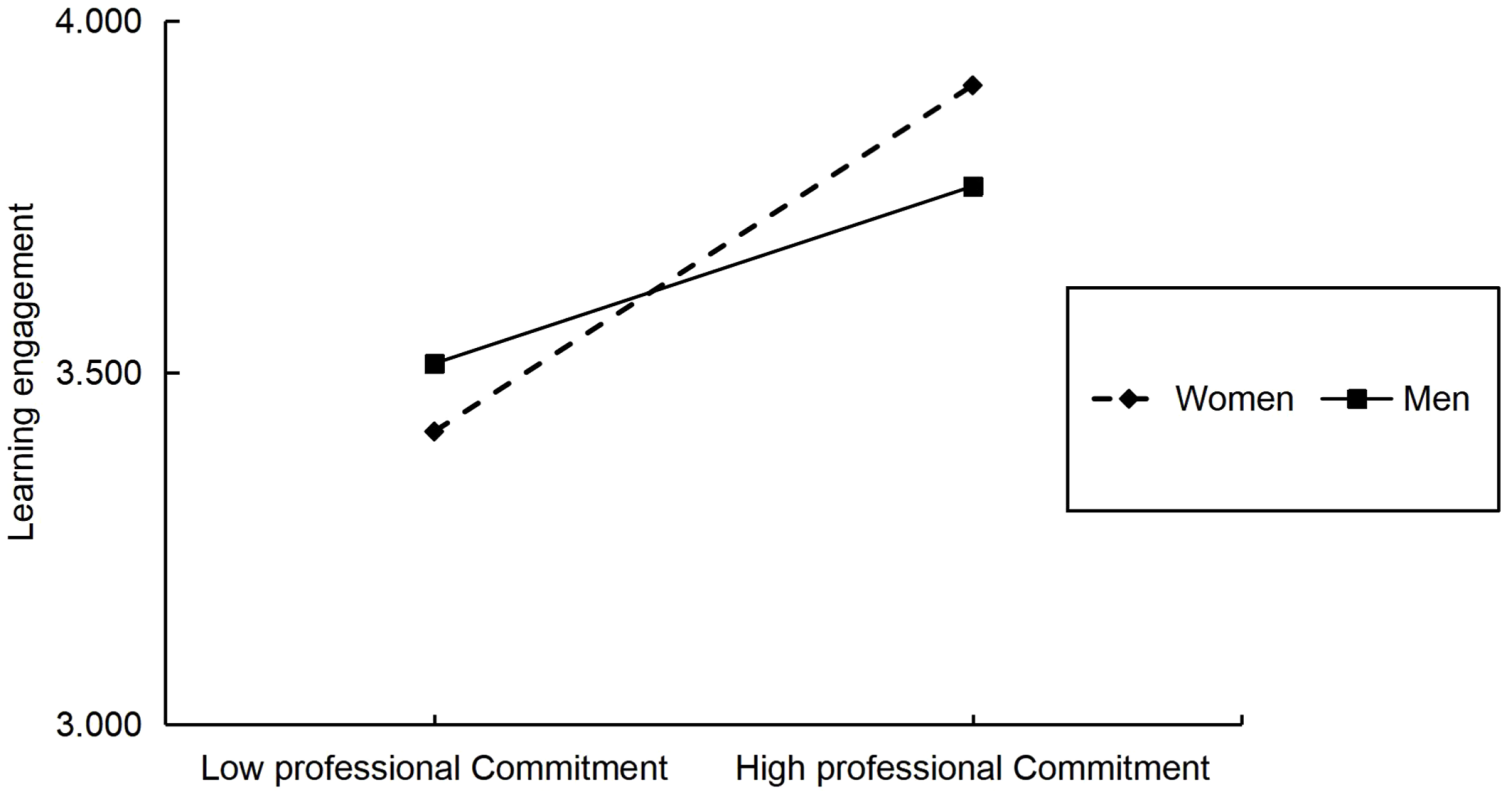
The moderating role of gender in learning engagement and professional commitment.

To advance our understanding of how professional commitment and gender moderate learning engagement, the results were analyzed by simple slope analysis using one standard deviation above and below the professional commitment scores and dividing professional commitment into high and low groups. The corresponding plots are shown in Figure 3.

Regarding female students, the growing influence of professional commitment on learning engagement showed a notable positive predictive impact (*β* = 0.444, *t* = 8.102, *p* < 0.001). In the case of males, professional commitment continued to be a key determinant of learning engagement (*β* = 0.228, *t* = 3.845, *p* < 0.001).

## 4 Discussion

This study integrates multiple theories, including social cognitive theory and the psychological resilience framework, to investigate the impact of academic self-efficacy on learning engagement. The findings elucidated the pathway through which academic self-efficacy influences learning engagement through psychological resilience and professional commitment, along with gender differences. The findings have theoretical and practical value for improving students’ learning engagement.

The existence of a direct link between academic self-efficacy and learning engagement was verified, corroborating Hypothesis 1. College students with strong self-belief in their academic abilities are better equipped to handle academic challenges. Conversely, students with weak academic self-efficacy tend to experience self-doubt and resist the execution of learning tasks, thereby avoiding academic failure (Allari et al., 2020). Maslow’s hierarchy of needs theory posits seven hierarchical needs: physiological, safety, belongingness and love, esteem, cognitive, esthetic, and self-actualization (Maslow, 1987). Maslow argues that satisfaction of lower-level needs is a prerequisite for achieving self-actualization. This theory suggests that students may lack strong learning motivation when certain needs are not met. When students anticipate positive learning outcomes and believe in their ability to complete learning tasks, their need for esteem and cognition becomes exceptionally strong. Once these needs are satisfied, higher-level knowledge-seeking needs emerge, and students continue to choose challenging tasks, willingly investing more resources into the learning process, thus demonstrating higher levels of engagement. Conversely, when students have adverse expectations about learning outcomes and doubt their own capabilities, they may worry about poor grades, leading to potential rejection by teachers and peers. This can result in reluctance to invest excessive energy in learning, potentially leading to learning fatigue and even truancy. In addition, self-doubt regarding one’s learning abilities may gradually lead to learned helplessness and feelings of inferiority. When belongingness, love, and esteem needs are not met, motivation for knowledge seeking tends to weaken.

It was discovered that academic self-efficacy influences learning engagement via psychological resilience, corroborating Hypothesis 2. Notably, academic self-efficacy encompasses not only students’ self-assurance in their academic abilities but also their belief in their capacity to complete academic assignments and overcome challenges; this conviction influences learning engagement by boosting psychological resilience. More specifically, psychological resilience originates from a specific belief system that encompasses one’s views of oneself, others, and the goodness and beauty of the world. This belief system is influenced by various factors associated with an individual’s life stages (Gillespie et al., 2007, Celik et al., 2015). Individual factors, such as attention, cognition, emotion, and behavior, can influence the cultivation of psychological resilience. The psychological resilience framework suggests that the reason why individuals experience different adaptation outcomes are determined by a combination of three factors: the environment, intra-individual factors, and individual-environment interactions (Luthar et al., 2000). Individuals who experience positive emotions during learning employ various effective strategies to augment their enthusiasm and engagement (Chmitorz et al., 2018). This study supports this theory and proves that individuals who possess high psychological resilience levels will have a more positive mood, a more optimistic attitude, a greater belief in their abilities, and engage in more positive actions when faced with learning tasks. This proactive behavior, in turn, motivates individuals to invest more effort in the learning process. Furthermore, studies indicate that psychological resilience is crucial not only in the face of stress and challenges, but also for improving daily learning adaptability; specifically, it supports the effective management of academic challenges and sustained learning engagement. Enhancing psychological resilience can thus enable university students to cultivate constructive educational mindsets and positive conduct patterns and is therefore crucial for boosting their academic achievements (Bandura, 1997).

This study’s results indicate that professional commitment plays a mediating role between academic self-efficacy and learning engagement, supporting Hypothesis 3. In particular, we found that students’ academic self-efficacy can indirectly impact their academic engagement, thereby enhancing their academic achievements. The results support social cognitive occupational theory, highlighting the significance of professional dedication in shaping how academic self-efficacy impacts behavior and outcomes. Students with strong academic self-efficacy indirectly boost their involvement in learning by strengthening their emotional bond with and excitement about their field of study. Students deeply dedicated to their profession frequently view their field of study not only as a means to just gain knowledge, but also as a crucial pathway to realizing their personal ethics and professional goals. Pursuing such a clear career objective encourages them to exert more effort and concentrate on academic pursuits. Along these lines, their engagement extends beyond classroom activities, including academic pursuits, internships, and research endeavors. Such beneficial educational practices boost not just their academic performance but also their self-assurance and professional identity, thereby creating a beneficial cycle. This finding aligns with earlier research reporting that college students with high levels of belief in their own academic abilities demonstrate relatively high levels of dedication to their fields of study, which, in turn, boosts their involvement in academic activities (Lent et al., 2002).

Through a professional commitment survey of over 400 medical students, Lu et al. (2023) confirmed the impact of self-efficacy on academic performance was validated by professional commitment and learning engagement. In other words, students who assess their learning abilities positively often express strong affection for their chosen profession. They have high expectations for development in their chosen field, willingly adhere to the norms and requirements of their chosen profession, believe in their ability to overcome internal and external challenges in learning, continuously experience and validate their ideas in practical learning, and invest energy into professional learning. Consequently, teachers and educational leaders can implement specific strategies to boost students’ academic achievements and professional identities, thereby advancing their comprehensive growth.

Ultimately, the findings suggest that gender plays a moderating role in how professional commitment impacts learning engagement, supporting Hypothesis 4. The results from the gender difference test indicate that professional commitment has a notable influence on the educational involvement of both male and female college students, and a more pronounced effect on female students. This result may be intimately linked to the conventional placement of gender roles or societal expectations. Through socialization, people develop gender cognitive frameworks via familial, educational, media, and various other mediums. Such cognitive schema regarding gender serve as an intrinsic structure influencing personal conduct and mindset, facilitating the demonstration of clear gender orientations in various contexts (Skaar et al., 2014). Accordingly, girls’ superior learning engagement may be partially attributed to Chinese societal and cultural traditions: in the Chinese context, girls are frequently motivated to comply and collaborate, and this may compel them to demonstrate strong academic engagement. On the one hand, girls tend to recognize their professional knowledge emotionally and subconsciously idealize it. Such an emotional identity encourages them to dedicate more time and effort to learning; that is, it increases their learning engagement (Meece et al., 2006). On the other hand, boys tend to be more rational and critical in their studies. Here, it is useful to note that social role theory suggests that boys tend to be encouraged to be more independent and innovative in their thinking and decision-making; this tendency may compel boys to approach academic tasks more holistically and objectively. Along these lines, boys tend to think more about their professions and identify with them more rationally and calmly. Although this rational way of thinking makes boys less emotionally involved than girls, they can still show higher academic engagement when they are highly committed (Eccles, 2009).

In sum, gender role cognition and social expectations influence students’ professional identification levels. Due to differing social expectations, girls tend to show a higher emotional attachment to their professional identity, while boys are more rational and objective. Despite both genders having a similar level of professional identity, female students are generally more actively engaged in their learning (Yan et al., 2023). This suggests that gender role cognition plays an important role in regulating academic behavior.

## 5 Conclusion

This research developed a moderated mediation model to elucidate the interconnection between academic self-efficacy and learning engagement. As a result, it was discovered that academic self-efficacy significantly forecasts college students’ learning engagement, and psychological resilience and professional commitment were discovered to mediate the link between academic self-efficacy and learning engagement in tandem, with the mediation of professional commitment being greater than that of psychological resilience. Academic self-efficacy’s role in predicting college students’ learning engagement was fully mediated by psychological resilience and professional commitment. Furthermore, the study revealed a gender moderation in the latter part of the pathway for professional commitment. Specifically, women exhibited stronger professional commitment than men, leading to elevated levels of learning engagement.

## 6 Implications and limitations

### 6.1 Implications

This study confirmed the inherent link between college students’ academic self-efficacy and learning engagement by developing a parallel mediation model of academic self-efficacy, psychological resilience, professional commitment, and learning engagement. The findings offer valuable insights useful for enhancing college students’ learning engagement. In the post-pandemic era, blended online and offline teaching methods have become dominant and student engagement has been popularly figured as a crucial determinant of overall learning quality. Consequently, enhancing students’ engagement levels has become increasingly vital to improving educational outcomes (Aparicio et al., 2023; Chen et al., 2022). Drawing from this study’s findings, interventions can be developed to enhance the psychological resilience and professional commitment of college students. Given the beneficial impacts of professional commitment, one approach is to encourage students to consider their individual characteristics and career preferences while choosing a college major. Students should thoroughly understand the study content, future employment directions, and prospects of the chosen major to enhance their emotional satisfaction with their field of study. Alternatively, students who cannot adapt to their chosen majors after a certain period during the first year should be allowed to make adjustments. School departments could support students by conducting career aptitude tests to help them choose a more suitable major. Leveraging the positive impact of psychological resilience, teachers could integrate positive psychology content, such as resilience education, into classrooms and daily activities to enhance college students’ learning engagement and increase their psychological resilience. For students facing psychological trauma and learning challenges due to COVID-19, focused interventions, such as psychological counseling, group counseling, and therapy, are required to facilitate their swift recovery of their initial level of psychological resilience. Furthermore, teachers should be mindful of gender disparities and implement specific teaching methods and support strategies to enhance learning engagement and professional identity among students of various genders. As an illustration, the academic achievements of female students can be boosted through the enhancement of their rational and critical thinking skills; meanwhile, the learning engagement of male students can be augmented by motivating them to share their emotions and form emotional bonds during their academic pursuits.

### 6.2 Limitations

While our study has yielded valuable insights, it involved several limitations that suggest promising directions for future research. First, our reliance on self-report measures introduced the potential for common method bias; although we mitigated the threat of this bias, we did not entirely eliminate it. Future research should consider employing a variety of data collection methods, such as behavioral observations and third-party assessments, to enhance the comprehensiveness and reliability of the findings. Second, the study’s cross-sectional design limited our ability to infer causal relationships among the variables. Therefore, future research should utilize experimental designs or longitudinal approaches to further clarify the dynamic and causal relationships among academic self-efficacy, professional commitment, resilience, and learning engagement over time. Third, our focus on university students from central China somewhat restricts the generalizability of the findings. To improve external validity, future research should include participants from diverse educational and cultural backgrounds both within other regions of China and internationally. By applying this broader approach, researchers may examine cultural differences and commonalities in the relationships among academic self-efficacy, professional commitment, resilience, and academic engagement, thereby contributing to the development of a more comprehensive and universally applicable theoretical framework. Finally, this study only conducted a preliminary exploration of the mediating and moderating roles of professional commitment and resilience in the relationship between academic self-efficacy and academic engagement. Given the complexity of learning behaviors and psychological processes, future research should consider additional potential mediators and moderators, such as parenting styles, peer support, and future orientation. By elucidating how these factors collectively impact academic engagement, future research may offer insights useful for developing more nuanced intervention strategies and recommendations for educational practice.

## Data Availability

The raw data supporting the conclusions of this article will be made available by the authors, without undue reservation.
