# Supplementary material for: The relationship between college students’ learning engagement and academic self-efficacy: a moderated mediation model
Source: Front Psychol. 2024 Sep 3;15:1425172. doi: 10.3389/fpsyg.2024.1425172 (PMC11407112; doi:10.3389/fpsyg.2024.1425172)
Supplement: Supplementary file 1 [file Data_Sheet_1.zip › supplementary materials/manuscript/English article/2024.1.3Aricle.docx]

****Relationship between College Students' Learning Engagement and Academic Self-efficacy in the Post-pandemic Era: A Moderated Mediation Model of Gender, Professional Commitment, and Psychological Resilience****

Introduction：In the post-pandemic era, although college students have returned to campus, the profound impact of COVID-19 on their learning approaches continues. Based on social cognitive theory, this study explores the relationships among academic self-efficacy, professional commitment, psychological resilience, and learning engagement in college students in the post-pandemic era. The study investigates how academic self-efficacy influences learning engagement with gender as a moderating variable and psychological resilience, and professional commitment as mediating variables.

Methods：Conducted a survey on 1032 college students in Henan Province, China, using the Psychological Resilience Scale, Academic Self-Efficacy Scale, College Student Learning Engagement Questionnaire, and College Student Professional Commitment Scale. Data were analyzed for mediating and moderating effects using SPSS and its process plugin.

Results：The results reveal a significant positive correlation between academic self-efficacy and college students' learning engagement. The positive prediction of academic self-efficacy on learning engagement is mediated by the fully parallel effects of psychological resilience and professional commitment, with the mediating effect of professional commitment being greater than that of psychological resilience. Further investigation indicates that the mediating effect of professional commitment is moderated by gender, and female students with stronger perceptions of professional commitment exhibit higher levels of learning engagement. Gender does not significantly moderate the effects of psychological resilience.

Conclusions：To elevate the level of learning engagement among college students, it is essential to focus on their academic self-efficacy, professional commitment, and psychological resilience levels.

Keywords: Learning Engagement, Academic Self-Efficacy, Professional Commitment, Psychological Resilience

**Introduction**

The level of learner's learning engagement is a concern for educators[1]. In the post-pandemic era, how to take effective measures to enhance college students' learning engagement is a topic of concern for many countries. Increasingly, research indicates that effective learning depends on the learners themselves[2]. Effective learning requires students to actively participate in learning, internalize acquired knowledge, and form their own learning experiences[3]. Learning engagement is an important factor influencing students' academic performance[4]. More and more countries are associating the level of learning engagement with academic performance, reward and punishment systems, dropout rates, and graduation rates.

Learning engagement is a crucial predictor of learning quality[5]. During the outbreak of the pandemic, college students predominantly engaged in home-based learning through the internet, leading to a substantial shift in the learning mode from traditional face-to-face collective learning to non-contact individual learning compared to the pre-pandemic period. As the pandemic concludes, students return to classrooms for face-to-face learning, but influenced by the learning mode during the pandemic, they exhibit low learning initiative and a decreased level of learning engagement. Hence, it is essential to explore strategies for improving college students' learning engagement levels in the post-pandemic era.

**Learning engagement**

Learning engagement refers to the time, energy, persistent and vigorous emotional states, and cognitive states invested by students in the learning process[6,7]. Several theories can explain the process of learning engagement, such as social cognitive theory and self-determination theory. Relevant to this paper is social cognitive theory, which posits that individual behavior is influenced by both social environmental factors and personal factors. Self-efficacy is an important theory within social cognitive theory.Bandura proposed that self-efficacy is influenced by the environment, impacting cognitive processes. Higher self-efficacy promotes individual cognitive development and subsequently enhances academic behavior. On the other hand, it also influences individual behavior[8]. Individuals with high self-efficacy tend to select challenging academic tasks and invest more effort. Even when encountering significant setbacks, they can quickly recover and continue pursuing their goals. Previous research has confirmed the social cognitive theory, revealing a close relationship between learning engagement and psychological resilience[9-12], as well as perceived learning ineffectiveness[13].

**The relationship between academic self-efficacy and learning engagement**

Self-efficacy refers to an individual's confidence and feelings about organizing and executing a specific task[14,15]. Self-efficacy was initially proposed by Bandura and is a crucial psychological factor strongly influencing people's behavior and performance. Self-efficacy includes two components: efficacy expectations and outcome expectations[16]. General self-efficacy is a broad concept, and after its introduction, various fields have conducted extensive research, leading to the development of derivative concepts such as academic self-efficacy and organizational self-efficacy.

Academic self-efficacy, which refers to an individual's confidence in their learning abilities, is a crucial aspect of learning[15]. It involves learners' confidence and feelings about organizing and executing specific learning tasks, successfully comprehending study materials. As a significant predictive variable in learning, it strongly influences students' learning behavior and performance, significantly impacting their level of learning engagement. Numerous studies have indicated a positive correlation between academic self-efficacy and learning engagement among college students. Those with strong academic self-efficacy demonstrate greater confidence in completing learning tasks and exhibit higher levels of engagement in their studies. On the contrary, students with lower academic self-efficacy may experience more helplessness, encounter increased negative emotions, and demonstrate reduced participation in their studies[17]. Academic self-efficacy encourages learners to adopt methods that align with their goals, exerting a substantial influence on completing learning tasks. Individuals with strong academic self-efficacy possess a good cognitive understanding of the learning process, attributing lack of success more to insufficient effort rather than lack of ability. The relationship between students' academic self-efficacy and learning engagement is closely intertwined[18]. Based on this, we posit Hypothesis 1: Academic self-efficacy positively predicts learning engagement.

**The mediating role of psychological resilience between academic self-efficacy and learning engagement**

Psychological resilience is a vital capability for individuals to enhance their capacity to cope with difficulties and effectively respond to sources of stress when facing challenges[19,20]. It is defined as the ability of individuals to maintain a good adaptive state or "bounce back" to normal life when encountering adversity, trauma, misfortune, or significant stressors[21]. The Psychological Resilience Framework Theory posits that individuals generate three adaptive outcomes when dealing with stress: the first is an increase in resilience levels; the second is maintaining the original level of resilience; the third is a decrease in resilience levels after being shocked by stress. The emergence of different adaptive outcomes is influenced by the environment, individual factors, and the interaction between individuals and the environment[22].This theory suggests that psychological resilience is dynamic and malleable, serving as an important protective factor in the process of psychological development[23]. Psychological resilience is not an inherent trait of personality; rather, it continually develops throughout an individual's entire life course, influenced by the surrounding living environment[24,25]. Leontopoulou's[26] study found that even in adversity, both positive coping strategies and avoidance coping strategies significantly impact psychological resilience. Individuals with good psychological resilience have strong adaptive capabilities and a high ability to absorb and utilize coping strategies. Individuals with positive emotions in learning apply various effective strategies to enhance their learning enthusiasm and engagement. Alazemi et al.'s[27] survey of high school students revealed that the higher the students' academic psychological resilience, the stronger their self-efficacy, and vice versa. The study results also indicate a close relationship between academic self-efficacy and psychological resilience.

According to this theory, psychological resilience is dynamic and flexible, serving as a crucial protective factor in the process of psychological development[23]. It is not an inherent trait of personality; instead, it continuously evolves throughout an individual's entire life, influenced by the surrounding living environment[24,25]. Leontopoulou's[26] study suggests that even in adversity, both positive coping strategies and avoidance coping strategies significantly influence psychological resilience. Individuals with robust psychological resilience exhibit strong adaptive capabilities and a high capacity to absorb and utilize coping strategies. Those with positive emotions in their learning endeavors apply various effective strategies to enhance their learning enthusiasm and participation. A survey conducted by Alazemi et al.[27] on high school students found that the higher the students' academic psychological resilience, the stronger their self-efficacy, and conversely, lower psychological resilience corresponds to lower self-efficacy. The study results also indicate a close relationship between academic self-efficacy, as an expression of self-efficacy in learning, and psychological resilience.Social Cognitive Theory emphasizes that individuals with self-efficacy beliefs have stronger convictions in successfully completing tasks, set more challenging goals, and, when facing difficulties, invest more energy and perseverance in coping. Wicaksono et al.[28], in their study on second language learning, found a close correlation between self-efficacy, perseverance, academic resilience, and academic demotivation. Self-efficacy and perseverance enable learners to experience more positive expectations for learning outcomes in the process of second language acquisition, enhance academic resilience, and thereby sustain more efficient learning engagement in the long run.Research by Shao and Kang [29] and others indicates a close relationship between academic psychological resilience, self-efficacy, and learning engagement. Students with academic psychological resilience, despite facing challenges, often have strong confidence in completing learning tasks and believe in their ability to do so. Consequently, they exhibit higher levels of learning engagement. Rajan et al.[30], in a survey of 155 high school students in India, found significant gender differences in academic resilience, with a positive correlation between high school students' academic resilience and self-efficacy. Based on these studies, the relationship among psychological resilience, academic self-efficacy, and learning engagement is close. However, the underlying mechanisms among the three still need clarification. Therefore, this study proposes Hypothesis 2: Psychological resilience plays a mediating role between academic self-efficacy and learning engagement.

1. **The mediating role of professional commitment in the relationship between academic self-efficacy and learning engagement**

Professional commitment reflects an individual's attitude and behavior towards their chosen major, signifying their identification with the major and willingness to invest time and effort in the field of study[31]. It is an expression of an individual's love and loyalty to their chosen major. Professional commitment serves as a crucial indicator for understanding the degree of students' engagement in their major studies. Previous studies have already demonstrated a significant correlation between professional commitment and learning engagement. Chen[32] conducted a survey with 750 university students majoring in preschool education, using a questionnaire to investigate the relationship between their satisfaction with learning, professional commitment, and learning engagement. The results revealed that students majoring in preschool education showed a moderate level of professional commitment, while scoring high in learning engagement. There was a significant positive correlation between learning engagement and professional commitment.

Research suggests a close relationship between self-efficacy and professional commitment, especially with emotional commitment. Tsai et al.'s[33] study suggests that a high level of self-efficacy has a positive impact on emotional commitment. This positive effect arises because individuals with high self-efficacy are more inclined to accept the goals and values of the organization compared to those with low self-efficacy. Orgambídez et al.'s[34] research confirms the close relationship between job involvement, self-efficacy, and affective organizational commitment. Individuals with good self-efficacy are more emotionally receptive to their workplace and are more willing to invest more energy in their work. Based on this, Hypothesis 3 is proposed: Professional commitment plays a mediating role between academic self-efficacy and learning engagement.

**5.Gender as a moderating factor in the relationship between professional commitment and learning engagement**

Gender is an important demographic variable that influences learning engagement. Due to the different cognitive structures of the male and female brains, male and female students have different preferences in cognitive engagement strategies. Compared to females, males have stronger information processing abilities and better metacognitive monitoring and regulatory strategies. Females focus more on external learning aids and cognitive strategy learning[35]. Secondly, there are gender differences in factors influencing learning engagement. Gender differentiation theory suggests that, with the physiological differentiation of gender, individuals gradually form gender role concepts in the process of social construction. This process implies the continuous growth of individuals and the advancement of the socialization process. Individuals of different genders participate in professional learning in their own way, and adjust their expectations of the major based on their understanding of the major formed through learning.The level of professional commitment constructed from this is also different, leading to different levels of learning engagement[32]. For example, males are more adapted to majors that cultivate hands-on skills and problem-solving abilities, resulting in more proactive and interactive learning behaviors in these majors. Females, on the other hand, prefer majors that cultivate reading skills and critical thinking abilities, leading to higher levels of learning engagement in these majors. From this, it can be inferred that gender differences may exist in the impact of professional commitment on college students' learning engagement behaviors. Based on this, Hypothesis 4 is proposed: Gender can moderate the relationship between professional commitment and learning engagement.

In summary, this study integrates social cognitive theory and attempts to construct a moderated parallel mediation model. It explores how college students' academic self-efficacy influences learning engagement behavior through the parallel mediating effects of psychological resilience and professional commitment, with a focus on the moderating role of gender. The goal is to provide insights for enhancing college students' learning participation.

**Subject**

Recruited through a cluster random sampling method, participants included undergraduate students from a university in Henan, China, ranging from freshmen to seniors. We utilized the anonymous survey platform "Wenjuanxing" to collect data, receiving a total of 1187 responses. After excluding incomplete or insincere responses, we obtained 1032 valid questionnaires. Participants consisted of 376 freshmen (36.4%), 273 sophomores (26.5%), 263 juniors (25.5%), and 120 seniors (11.6%). Among the participants, there were 479 males (46.4%) and 553 females (53.6%). The sample included 220 student cadres (21.3%) and 812 non-cadres (78.7%). Additionally, there were 148 only children (14.3%) and 884 non-only children (85.7%). In terms of major selection, 732 participants (70.9%) autonomously chose their majors during the college entrance examination, 107 participants (10.4%) followed their parents' and others' wishes, and 193 participants (18.7%) adjusted their majors according to arrangements.

The study was conducted from March to September 2023. The research obtained approval from the Academic Committee of Huanghuai University and was tested through an online survey platform called "Wenjuanxing," with participants collectively tested by class. Informed consent was obtained from all participants before testing.

**Psychological Resilience**

In this study, the Chinese version of the Resilience Scale (CD-RISC) [36], translated and revised by Chinese scholars Yu and Zhang, was used. Developed by American psychologists Connor and Davidson in 2003, CD-RISC includes three dimensions: self-improvement, toughness, and optimism, with a total of 25 items. It adopts a Likert 5-point rating scale, with scores ranging from "never" to "almost always" recorded as 0-4 points. The total score is in the range of (0-100), and a higher score indicates a better level of psychological resilience. The Cronbach's α coefficient for the Chinese version of CD-RISC is 0.916. In this study, the Cronbach's α coefficient is 0.963.

**Academic Self-Efficacy**

In this study, the "Academic Self-Efficacy Scale" [37], developed by Liang Yusong in 2004, was used. The scale consists of 22 items, including two dimensions: self-efficacy for learning ability and self-efficacy for learning behavior. The scale adopts a 5-level rating standard, with scores ranging from "strongly disagree" to "strongly agree" recorded as 1-5 points. Reverse scoring is applied to questions 14, 16, 17, and 20, while other questions are scored positively. A higher questionnaire score indicates a higher level of academic self-efficacy in students. In this study, the Cronbach's α coefficient is 0.915.

**Learning Engagement**

The College Student Learning Engagement Questionnaire is used to assess the level of learning engagement[38]. The questionnaire consists of 20 items, including three dimensions: behavioral engagement, cognitive engagement, and emotional engagement. A five-point scoring system is used. A higher score indicates a higher level of learning engagement for the participants. The Cronbach's α coefficients for the College Student Learning Engagement Questionnaire and the three sub-scales (behavioral engagement, cognitive engagement, and emotional engagement) are 0.918, 0.825, 0.858, and 0.858, respectively. The Cronbach's α coefficient for the entire scale is 0.918, indicating good reliability. In this study, the Cronbach's α coefficient is 0.969.

**Professional Commitment**

The "College Students' Professional Commitment Scale"[39], developed by Lian Rong and others, is used in this study. The scale consists of 27 items, organized into dimensions such as affective commitment, continuance commitment, normative commitment, and ideal commitment. Scores are calculated using a Likert 5-point scoring method, ranging from "completely disagree" to "completely agree" (scored 1-5). Items 6, 8, and 12 are reverse-scored. A higher score indicates a higher level of professional commitment. According to Lian Rong et al.'s research, the internal consistency Cronbach's α coefficient of the scale is 0.92. In this study, the Cronbach's α coefficient is 0.955.

**Common Method Bias Test**

The data in this study are all from self-reported measures by the participants, which may lead to common method bias. Therefore, Harman's single-factor test was conducted to examine it. The results show that there are 11 factors with eigenvalues greater than 1, and the first factor explains 29.766% of the variance, which is below the critical standard of 40%[40]. Thus, there is no serious common method bias in this study.

The results in Table 1 indicate a positive correlation between academic self-efficacy and psychological resilience, professional commitment, and learning engagement. There is a positive correlation between psychological resilience and professional commitment as well as learning engagement. Professional commitment is positively correlated with learning engagement.

The study employed model 4 in the SPSS macro PROCESS developed by Hayes [41] to test the mediating effects of psychological resilience and professional commitment between academic self-efficacy and learning engagement. The results, as shown in Table 2 and Figure 2, indicate that, when controlling for variables such as gender, grade, leadership role, only child status, and hometown, academic self-efficacy positively predicts psychological resilience (β= 0.436, p＜0.001) and professional commitment (β= 0.640, p＜0.001). Both psychological resilience and professional commitment predict positive learning engagement (β= 0.312, p＜0.001; β= 0.263, p＜0.001). However, the direct predictive effect of academic self-efficacy on learning engagement becomes non-significant (β=0.001, p=0.976). This suggests that the direct effect of academic self-efficacy on learning engagement is not significant, and professional commitment and psychological resilience play a fully mediating role between academic self-efficacy and learning engagement.

The analysis results reveal that the mediating effect value for academic self-efficacy → psychological resilience → learning engagement is 0.136, and for academic self-efficacy → professional commitment → learning engagement is 0.170. The 95% confidence interval of the effect values does not include 0, indicating that psychological resilience and professional commitment have significant mediating effects between college students' academic self-efficacy and learning engagement. Psychological resilience and professional commitment play a complete mediating role in the impact of college students' academic self-efficacy on learning engagement.

In order to delve deeper into the reasons behind gender disparities in professional commitment, Model 14 was applied to test the moderating role of gender in the original parallel mediation model. The results, presented in Table 3, indicate that the significance of the original paths aligns with previous observations. Gender demonstrates a significant moderating effect on the latter segment of professional commitment mediation (β=-0.217, p＜0.01), whereas the moderating effects on the initial part of professional commitment and both segments of psychological resilience mediation are not statistically significant.

To further investigate the moderating effects of professional commitment and gender on learning engagement, we categorized professional commitment scores into high and low commitment groups based on one standard deviation above and below the mean. Simple slope analyses were conducted on the results, and the corresponding plots can be found in Figure 2.

Regarding female students, the impact of professional commitment on learning engagement shows an increasing trend, and the positive predictive effect of professional commitment is significant (β=0.445, t=8.107, p＜0.001). As for male students, the predictive effect of professional commitment on learning engagement remains significant (β= 0.228, t=3.843, p＜0.001).

**Discussion**

Combining social cognitive theory with the framework of psychological resilience, this study investigates the impact of academic self-efficacy on learning engagement behavior. It reveals the mechanisms through which academic self-efficacy influences learning engagement via psychological resilience and professional commitment, as well as gender differences. The findings have both theoretical and practical significance for enhancing students' levels of learning engagement.

Academic self-efficacy and learning engagement are positively correlated, confirming Hypothesis 1. Students with strong academic self-efficacy can effectively navigate academic challenges. Conversely, students with weak academic self-efficacy are more likely to harbor self-doubt and resist executing learning tasks, thereby avoiding academic failure [42]. Maslow's hierarchy of needs theory introduces seven hierarchical needs, ranging from physiological needs to safety needs, belongingness and love needs, esteem needs, cognitive needs, aesthetic needs, and self-actualization needs. Maslow argues that satisfying lower-level needs is a prerequisite for achieving self-actualization. This theory suggests that students may lack strong learning motivation when certain levels of needs are not met.When students anticipate positive learning outcomes and believe in their ability to complete learning tasks, the needs for respect and cognition become exceptionally strong. Once these needs are satisfied, higher-level knowledge-seeking needs emerge, and students continue to choose challenging tasks, willingly investing more resources into the learning process, demonstrating higher levels of engagement. Conversely, when students have adverse expectations about learning outcomes and doubt their own capabilities, they may worry about poor grades leading to rejection by teachers and peers. This can result in a reluctance to invest excessive energy in learning, leading to potential learning fatigue and even truancy. Additionally, self-doubt about their learning abilities may gradually lead to learned helplessness and feelings of inferiority. In general, when belongingness and love needs, as well as esteem needs, are not met, the motivation for knowledge-seeking tends to weaken.

The study finds that academic self-efficacy influences academic engagement through psychological resilience, confirming Hypothesis 2. Individual factors such as attention, cognition, emotion, and behavior can influence the development of psychological resilience. According to the theory of psychological resilience, the diverse adaptation outcomes of individuals are determined by the joint influence of environmental factors, internal individual factors, and the interaction between individuals and their environment. The personal factors of psychological resilience consist of cognitive, emotional, physical, mental, and behavioral aspects. Positive emotions can broaden the individual's attention and cognition, as well as continuously build personal positive resources, enhancing behavioral positivity [43].This study supports this theory, confirming that individuals with high levels of psychological resilience tend to have more positive emotions and a more optimistic attitude. When facing learning tasks, they are more likely to believe in their abilities and take more proactive actions. This proactive behavior leads individuals to invest more energy in the learning process. Psychological resilience originates from a specific belief system, which includes one's views on oneself, others, and the goodness and beauty of the world. The belief system is influenced by various factors related to the individual's life stage [44].

The results of this study indicate that professional commitment plays a mediating role between academic self-efficacy and learning engagement, confirming Hypothesis 3. This aligns with previous research findings. Zhou and Wu[45] demonstrated that professional commitment, academic self-efficacy, teacher support, and learning engagement are pairwise correlated. Professional commitment and academic self-efficacy significantly positively predict learning engagement. Lu et al.[46], in a survey of over 400 medical students on professional commitment, found that self-efficacy affects academic performance through professional commitment and learning engagement. In other words, students who have a good assessment of their learning abilities often express strong affection for their chosen profession, have high expectations for the development of their chosen field, are willing to adhere to the norms and requirements of their chosen profession, believe in their ability to overcome internal and external challenges in learning, continuously experience and validate their ideas in practical learning, and invest more and more energy into professional learning.

This study found that the mediating role of academic self-efficacy in the relationship between professional commitment and learning engagement is moderated by gender in the latter part, supporting hypothesis 4.This may be closely related, on the one hand, to the traditional positioning of gender roles or societal expectations.This may be closely related, on the one hand, to the traditional positioning of gender roles or societal expectations.During the process of socialization, individuals acquire gender cognitive schemas, and these schemas lead individuals to exhibit distinct gender tendencies [47].Females tend to be more emotional and compliant, emotionally endorsing their chosen major, unconsciously idealizing their academic pursuits, and willingly investing more energy into their studies.In contrast, males tend to be more rational, seek novelty and diversity in their thoughts, exhibit a stronger sense of control, and provide a more comprehensive and objective evaluation of their chosen major.Students are easily influenced by the ingrained cognitive schemas and implicit expectations of gender roles, leading to gender differences in the level of professional identification.

**Conclusion**

This study constructed a moderated mediation model to explain the relationship between academic self-efficacy and learning engagement.

Findings indicated that academic self-efficacy significantly and positively predicted college students' learning engagement. Both psychological resilience and professional commitment played parallel mediating roles between academic self-efficacy and learning engagement, with the mediating effect of professional commitment being greater than the positive mediating effect of psychological resilience.The predictive role of academic self-efficacy in college students' learning engagement is entirely mediated by psychological resilience and professional commitment.Additionally, the research identified gender moderation in the latter part of the pathway for professional commitment. Specifically, females demonstrated stronger professional commitment compared to males, resulting in higher levels of learning engagement.

**Research Value and Limitations**

****Research Value****

This study has theoretical and practical significance in exploring how to enhance learning engagement in the post-pandemic era.Firstly, this study attempts to construct a mediated model where academic self-efficacy influences learning engagement through psychological resilience and professional commitment. It supplements factors related to the impact of self-efficacy on learning engagement in the social cognitive theory, expanding the pathways through which academic self-efficacy affects learning engagement. This provides a theoretical foundation for a deeper understanding of the mechanisms by which academic self-efficacy influences learning engagement.Secondly, this study holds crucial practical significance in enhancing college students' learning engagement. In the post-pandemic era, blended learning has become a trend, and learning engagement is a key factor influencing the quality of online learning. Therefore, effectively elevating students' level of learning engagement becomes particularly important.According to the findings of this study, interventions can be initiated by improving both the levels of psychological resilience and professional commitment among college students.Based on the positive effects of professional commitment, one approach is to encourage students to consider their individual characteristics and career preferences when choosing their college majors. They should thoroughly understand the study content, future employment directions, and prospects of the chosen major to enhance emotional satisfaction with the field of study.On the other hand, after a certain period in the first year, students who cannot adapt to their chosen major should be allowed to make adjustments.School departments can support students by conducting career aptitude tests to assist them in selecting a more suitable major.Based on the positive effects of psychological resilience, to enhance college students' level of learning engagement, incorporate positive psychology content such as resilience education into classrooms and daily activities, aiming to elevate students' psychological resilience.Specifically, for students experiencing psychological trauma and learning difficulties due to COVID-19, conduct targeted activities such as psychological counseling, group counseling, and therapy to help them quickly regain their original level of psychological resilience.

**Limitations**

This study has some limitations. Firstly, the research data is based on self-reporting, introducing inherent reporting biases that are challenging to eliminate.Secondly, this study utilizes a cross-sectional design, hindering a thorough examination of causal relationships between variables. Future research could employ experimental designs and longitudinal studies to further substantiate the causal relationships among variables.Thirdly, this study exclusively explores the impact of professional commitment and psychological resilience on the relationship between academic self-efficacy and learning engagement. Future studies should consider additional variables with potential mediating or moderating effects, such as parental parenting styles, peer support, future orientation, and more.

**Reference**

1.Zheng C. Student Engagement and Academic Performance during the COVID-19 Pandemic: Does a Blended Learning Approach Matter?
International Journal for the Scholarship of Teaching and Learning*.*2023;17(1):1–9. https://doi.org/10.20429/ijsotl.2023.17107

2.Kumar S,Todd G.Effectiveness of online learning interventions on student engagement and academic performance amongst first-year students in allied health disciplines: A systematic review of the literature. Focus Health Prof Ed.2022;23(3);36–55. https://doi.org/10.11157/fohpe.v23i3.430

3.Rashid T, Asghar HM. Technology use, self-directed learning, student engagement and academic performance: Examining the interrelations. Comput Hum Behav*.*2016;63; 604–612. https://doi.org/10.1016/j.chb.2016.05.084

4.Sahni J. Is Learning Analytics the Future of Online Education?: Assessing Student Engagement and Academic Performance in the Online Learning Environment.
Int J Emerg Technol*.* 2023;18(2);33–49. https://doi.org/10.3991/ijet.v18i02.32167

5.Bayoumy HMM, Alsayed S. Investigating Relationship of Perceived Learning Engagement, Motivation, and Academic Performance Among Nursing Students: A Multisite Study. Adv Med Educ Pract.2021;12;351–369. https://doi.org/10.2147/AMEP.S272745

6.Fredricks JA, Blumenfeld PC, Paris AH. School Engagement: Potential of the Concept, State of the Evidence. Rev Educ Res*.* 2004;74; 59–109. https://doi.org/10.3102/00346543074001059

7.Schaufeli WB, Martínez IM, Pinto AM, Salanova M, Bakker AB.Burnout and Engagement in University Students: A Cross-National Study. J Cross Cult Psychol*.*2002; 33(5);464–481. https://doi.org/10.1177/0022022102033005003

8.Bandura A. On the Functional Properties of Perceived Self-Efficacy Revisited. J Manage. 2012;38(1);9–44. https://doi.org/10.1177/0149206311410606

9.Hartley MT.Examining the Relationships Between Resilience, Mental Health, and Academic Persistence in Undergraduate College Students. J Am Coll Health*.* 2011;59(7); 596–604. https://doi.org/10.1080/07448481.2010.515632

10.Smith BW, Dalen J, Wiggins K, Tooley E, Christopher P, Bernard J.The brief resilience scale: Assessing the ability to bounce back. *International Journal of Behavioral Medicine.* 2008 ; 15(3);194–200. https://doi.org/10.1080/10705500802222972

11..Zeng G, Hou H, Peng K.Effect of Growth Mindset on School Engagement and Psychological Well-Being of Chinese Primary and Middle School Students: The Mediating Role of Resilience.Front Psychol. 2016;7;1664-1078. https://doi.org/10.3389/fpsyg.2016.01873

12.Zhao H, Xiong J, Zhang Z, Qi C.Growth mindset and college Students' learning engagement during the COVID-19 pandemic: A serial mediation model. Front Psychol. 2021;12; 1664-1078.

https:// doi.org/10.3389/fpsyg.2021.621094

1. Ye JR, Wu YF, Nong W,Wu YT, Ye JN, Sun Y. The Association of Short-Video Problematic Use, Learning Engagement, and Perceived Learning Ineffectiveness among Chinese Vocational Students. Healthcare ;2023;11(2);161. https://doi.org/10.3390/healthcare11020161

14.Bandura A. Social Foundations of Thought and Action: A Social Cognitive Theory.Upper Saddle River;Prentice-Hall Press;1986 .

15.Bandura A.Self-Efficacy: The Exercise of Control.New York；W.H. Freeman & Company；1997.

16.You W.Research on the Relationship between Learning Engagement and Learning Completion of Online Learning Students. Int J Emerg Technol.2022; 17(1);102–117. https://doi.org/10.3991/ijet.v17i01.28545

17.Namaziandost E, Heydarnejad T, Saeedian S.Language Teacher Professional Identity: The Mediator Role of L2 Grit, Critical Thinking, Resilience, and Self-efficacy Beliefs . Iranian Journal of Applied Language Studies*.*2023; 14(2);107-130. https://doi.org/10.22111/IJALS.2022.7486

18.Xie D, Xie Z. Effects of Undergraduates’ Academic Self-Efficacy on Their Academic Help-Seeking Behaviors: The Mediating Effect of Professional Commitment and the Moderating Effect of Gender.J Coll Student Dev. 2019;60(3); 365–371.https://doi.org/10.1353/csd.2019.0035

19.Ahern NR,Norris AE .Examining factors that increase and decrease stress in adolescent community college students.J Pediatr Nurs.2011;.26(6);530-540.https://doi.org/530-540.10.1016/j.pedn.2010.07.011

20.Cooper AL, Brown JA, Rees CS, Leslie GD.Nurse resilience: A concept analysis. Int J Ment Health Nu , 2020; 29(4);553-575.https://doi.org/10.1111/inm.12721

21.Kumpfer KL.Factors and Processes Contributing to Resilience.2002 ; in: Glantz MD., Johnson JL. Resilience and Development, Longitudinal Research in the Social and Behavioral Sciences: An Interdisciplinary Series. Kluwer Academic Publishers, Boston, 179–224. https://doi.org/10.1007/0-306-47167-1_9

22.Luthar SS, Cicchetti D, Becker B. The construct of resilience: a critical evaluation and guidelines for future work. Child Dev,2000;71(3);543–562. <https://doi.org/10.1111/1467-8624.00164>

23.Cheung VHM, Chan CY, Au, RKC. The influence of resilience and coping strategies on suicidal ideation among Chinese undergraduate freshmen in Hong Kong. Asia-Pac Psychiat .2019;11(2);1758-5864. https://doi.org/10.1111/appy.12339

24.Gillespie BM, Chaboyer W, Wallis M. Development of a theoretically derived model of resilience through concept analysis*.*Contemp Nurse.2007;25(1-2);124-135https://doi.org/10.5172/conu.2007.25.1-2.124

25.Celik DA, Cetin F, Tutkun E. The role of proximal and distal resilience factors and locus of control in understanding hpe, self-esteem and academic achievement among Turkish pre-adolescents.Curr Psychol. 2015;34(2); 321- 345.

26.Leontopoulou.S. Resilience of Greek Youth at an Educational Transition Point: The Role of Locus of Control and Coping Strategies as Resources.Soc Indic Res .2006;76(1); 95–126. https://doi.org/10.1007/s11205-005-4858-3

27.Alazemi AFT, Jember B, Al-Rashidi AH. How to decrease Test Anxiety: a focus on Academic Emotion Regulation, L2 grit, resilience, and self-assessment. Lang Test Asia*.*2023;13(1); 1-17. https://doi.org/10.1186/s40468-023-00241-5

28.Wicaksono BH, Ismail SM, Sultanova SA, Abeba D.I like language assessment: EFL learners’ voices about self-assessment, self-efficacy, grit tendencies, academic resilience, and academic demotivation in online instruction. Lang Test Asia. 2023; 13(1);1-18. https://doi.org/10.1186/s40468-023-00252-2

29..Shao Y, Kang S. The association between peer relationship and learning engagement among adolescents: The chain mediating roles of self-efficacy and academic resilience. Front Psychol.2022; 13; 938756. https://doi.org/10.3389/fpsyg.2022.938756

30.Rajan SK, Harifa PR, Pienyu R. Academic resilience, locus of control, academic engagement and self-efficacy among the school children.Indian J Posit Psychol*.*2017; 8(4); 507–511.

31.Lian R, Yang LX, Wu LH. Relationship between professional commitment and learning burnout of undergraduates and scales developing. J Psychol*.*2005;37(05);632–636. https://kns.cnki.net/kcms/detail/detail.aspx?FileName= XLXB200505008&DbName=CJFQ2005

32.Chen M.Effect of Professional Satisfaction on Learning Engagement in Undergraduates Major in Preschool Education: Mediating Role of Professional Commitment. Psychol, 2018;9(8);2250–2260. https://doi.org/10.4236/psych.2018.98128

33.Tsai CW, Tsai SH, Chen YY, Lee WL. A study of nursing competency, career self-efficacy and professional commitment among nurses in Taiwan. Contemp Nurse*.*2014; 49(1);96–102. https://doi.org/10.1080/10376178.2014.11081959

34.Orgambídez A, BorregoY, Vázquez‐Aguado O.Self‐efficacy and organizational commitment among Spanish nurses: the role of work engagement. Int Nurs Rev.2019;66(3). 381–388. https://doi.org/10.1111/inr.12526

35.Liu R D. On the Essence of Learning Strategies .*Acta Psychologica Sinica.* 1997;179–181. https://doi.org/10.16719/j.cnki.1671-6981.1997.02.024

36.Yu XN,Zhang JX.A Comparison between the Chinese Version of Ego-Resiliency Scale and Connor-Davidson Resilience Scale[ J].Acta Psychol Sin.2007;169 (5): 1169-1171. https://doi.org/10.16719/j.cnki.1671-6981.2007.05.035

37.Liang SY. Study On Achievement Goals、Attribution Styles and Academic Self- efficacy of Collage Students.(master’s thesis). Central China Normal University;2000.

38.Ni KX.Study on the relationship between college students' learning engagement and subjective well-being -- a case study of six universities in chengdu. (master’s thesis).Chengdu University of Technology;2020. https://doi.org/10.26986/d.cnki.gcdlc.2020.001297

39.Lian R,Yang LX, Wu LH.Relationship between Professional Commitment andLesrning Burnout of Undergraduates and Scales Developing .Acta Psychol Sin.2005;37(5);632-636.

40.Zhou H, Long L R. Statistical test and control of common method deviation. Advances in Psychological Science,2004; 12(6);942-942.

41.Hayes AF. An Index and Test of Linear Moderated Mediation. Multivar Behav Res .2015;50(1); 1–22. https://doi.org/10.1080/00273171.2014.962683

42.Allari RS, Atout M.Hasan AAThe value of caring behavior and its impact on students’ self‐efficacy: Perceptions of undergraduate nursing students. Nurs Forum*.*2020; 55(2; 259–266. https://doi.org/10.1111/nuf.12424

43.Chmitorz A, Kunzler A, Helmreich I, Tüscher O, Kalisch R, Kubiak T, Wessa M, LiebK. Intervention studies to foster resilience – A systematic review and proposal for a resilience framework in future intervention studies. Clin Psychol Rev*,* 2018;59;78–100. https://doi.org/10.1016/j.cpr.2017.11.002

44.Jew C , Green K, Kroger J. Development and Validation of a Measure of Resiliency. Meas Eval Couns Dev.1999; 32; 75–89. https://doi.org/10.1080/07481756.1999.12068973

45.Parnikh H, Torabizadeh C, Kalyani MN , Soltanian M . A Study of the Relationship between Professional Communication and Professional Commitment in Operating Room Nurses. Nurs Res Pract.2022;2(6);57-68.https://doi.org/10.1155/2022/5871846

46.Lu Y,Tong K,Wen MG,Gong YY, Zhuang D,Zhu HY. Professional commitment of eight-year medical doctoral degree program students in China: the mediating role of self-efficacy, learning engagement, and academic performance. Bmc Med Educ.2023;(A [preprint](https://www.researchsquare.com/researchers/preprints)). https://doi.org/10.21203/rs.3.rs-3426236/v1

47.Skaar NR, Christ TJ, Jacobucci R. Measuring Adolescent Prosocial and Health Risk Behavior in Schools: Initial Development of a Screening Measure. Sch Ment Health*,*2014; 6(2); 137–149. https://doi.org/10.1007/s12310-014-9123-y
