# Supplementary material for: The relationship between college students’ learning engagement and academic self-efficacy: a moderated mediation model
Source: Front Psychol. 2024 Sep 3;15:1425172. doi: 10.3389/fpsyg.2024.1425172 (PMC11407112; doi:10.3389/fpsyg.2024.1425172)
Supplement: Supplementary file 1 [file Data_Sheet_1.zip › supplementary materials/manuscript/English article/2024.1.5Aricle.docx]

**Relationship between College Students' Learning Engagement and Academic Self-efficacy :** **A Moderated Mediation Model**

**Introduction：**In the post-pandemic era, despite the return of college students to campus, the profound impact of COVID-19 on their learning approaches persists. Drawing upon social cognitive theory, this study investigates the interconnections among academic self-efficacy, professional commitment, psychological resilience, and learning engagement among college students in the post-pandemic era. The study explores the influence of academic self-efficacy on learning engagement, considering gender as a moderating variable, and examining psychological resilience and professional commitment as mediating variables.

Methods：Conducted a survey involving 1032 college students in Henan Province, China, utilizing the Psychological Resilience Scale, Academic Self-Efficacy Scale, College Student Learning Engagement Questionnaire, and College Student Professional Commitment Scale.The data underwent analysis to assess mediating and moderating effects through SPSS, including its process plugin.

Results：The results unveil a noteworthy positive correlation between academic self-efficacy and learning engagement among college students. Academic self-efficacy's positive influence on learning engagement is fully mediated by the parallel effects of psychological resilience and professional commitment, with the mediating impact of professional commitment surpassing that of psychological resilience. Subsequent analysis indicates gender moderation in the mediating effect of professional commitment, with female students demonstrating stronger perceptions of professional commitment associated with elevated levels of learning engagement. Gender does not exhibit significant moderation effects on psychological resilience.

Conclusions：To enhance learning engagement among college students, it is imperative to address their levels of academic self-efficacy, professional commitment, and psychological resilience

**Keywords:** Learning Engagement, Academic Self-Efficacy, Professional Commitment, Psychological Resilience

**Introduction**

Educators are concerned about the level of learners' engagement[1]. In the post-pandemic era, the effective implementation of measures to enhance learning engagement among college students is a concern for many countries. Increasingly, research suggests that effective learning hinges on the learners themselves[2]. Effective learning necessitates students' active participation, internalization of acquired knowledge, and the formation of their own learning experiences[3]. Learning engagement constitutes a crucial factor influencing students' academic performance[4]. An increasing number of countries are linking the level of learning engagement with academic performance, reward and punishment systems, dropout rates, and graduation rates.

Learning engagement serves as a crucial predictor of the quality of learning[5]. Amid the pandemic outbreak, college students predominantly participated in home-based learning through the internet, resulting in a significant shift in the learning mode from traditional face-to-face collective learning to non-contact individual learning compared to the pre-pandemic period. With the conclusion of the pandemic, students return to classrooms for face-to-face learning. However, influenced by the learning mode during the pandemic, they demonstrate low learning initiative and a diminished level of learning engagement. Therefore, it is imperative to explore strategies for enhancing the levels of learning engagement among college students in the post-pandemic era.

**Learning engagement**

Learning engagement encompasses the time, energy, persistent and vigorous emotional states, and cognitive states invested by students in the learning process[6,7]. Various theories can elucidate the process of learning engagement, including social cognitive theory and self-determination theory. Pertinent to this paper is social cognitive theory, which posits that individual behavior is influenced by both social environmental factors and personal factors. According to Bandura, self-efficacy is influenced by the environment, impacting cognitive processes. Conversely, it also influences individual behavior[8]. Individuals with high self-efficacy are inclined to select challenging academic tasks and invest more effort. Moreover, when encountering significant setbacks, they can swiftly recover and persist in pursuing their goals. Prior research has substantiated social cognitive theory, disclosing a close relationship between learning engagement and psychological resilience[9-12], as well as perceived learning ineffectiveness[13].

**The relationship between academic self-efficacy and learning engagement**

Self-efficacy pertains to an individual's confidence and feelings regarding the organization and execution of a specific task[14,15]. Bandura initially proposed self-efficacy, and it stands as a crucial psychological factor significantly influencing individuals' behavior and performance. Self-efficacy comprises two components: efficacy expectations and outcome expectations[16]. General self-efficacy is a comprehensive concept, and subsequent to its introduction, diverse fields have undertaken extensive research, resulting in the development of derivative concepts like academic self-efficacy and organizational self-efficacy.

窗体顶端

窗体底端

Academic self-efficacy, denoting an individual's confidence in their learning abilities, stands as a crucial aspect of the learning process[15]. It encompasses learners' confidence and feelings regarding the organization and execution of specific learning tasks, as well as the successful comprehension of study materials. As a significant predictive factor in learning, it profoundly influences students' learning behavior and performance, thereby significantly impacting their level of learning engagement. Numerous studies have evidenced a positive correlation between academic self-efficacy and learning engagement among college students. Individuals with robust academic self-efficacy showcase heightened confidence in completing learning tasks and demonstrate elevated levels of engagement in their studies.Conversely, students with lower academic self-efficacy may experience heightened feelings of helplessness, encounter increased negative emotions, and exhibit reduced participation in their studies[17]. Academic self-efficacy motivates learners to adopt methods aligned with their goals, exerting a substantial influence on the completion of learning tasks. Individuals with robust academic self-efficacy possess a solid cognitive understanding of the learning process, attributing lack of success more to insufficient effort rather than lack of ability. The relationship between students' academic self-efficacy and learning engagement is intricately intertwined[18]. Based on this, we propose Hypothesis 1: Academic self-efficacy positively predicts learning engagement.

**The mediating role of psychological resilience between academic self-efficacy and learning engagement**

Psychological resilience is a crucial capability enabling individuals to enhance their capacity to cope with difficulties and respond effectively to sources of stress when confronting challenges[19,20]. It is defined as the ability of individuals to maintain a positive adaptive state or "bounce back" to normal life when facing adversity, trauma, misfortune, or significant stressors[21]. The Psychological Resilience Framework Theory posits that individuals generate three adaptive outcomes when dealing with stress: the first is an increase in resilience levels; the second is maintaining the original level of resilience; the third is a decrease in resilience levels after experiencing the shock of stress. The emergence of various adaptive outcomes is influenced by the environment, individual factors, and the interaction between individuals and the environment[22].This theory proposes that psychological resilience is dynamic and malleable, playing a crucial role as a protective factor in the process of psychological development[23]. Psychological resilience is not an inherent personality trait; instead, it continuously develops throughout an individual's entire life course, influenced by the surrounding living environment[24,25]. Leontopoulou's[26] study found that even in the face of adversity, both positive coping strategies and avoidance coping strategies significantly influence psychological resilience.Individuals with robust psychological resilience exhibit strong adaptive capabilities and a high capacity to absorb and utilize coping strategies. Individuals experiencing positive emotions in learning employ various effective strategies to augment their learning enthusiasm and engagement. Alazemi et al.'s[27] survey of high school students revealed that the higher the students' academic psychological resilience, the stronger their self-efficacy; conversely, a higher level of self-efficacy is associated with increased academic psychological resilience. The study results also signify a close relationship between academic self-efficacy and psychological resilience.

According to this theory, psychological resilience is dynamic and flexible, playing a pivotal role as a protective factor in the process of psychological development[23]. It is not an inherent personality trait; rather, it continuously evolves throughout an individual's entire life, influenced by the surrounding living environment[24,25]. Leontopoulou's[26] study suggests that even in the face of adversity, both positive coping strategies and avoidance coping strategies significantly influence psychological resilience. Individuals with robust psychological resilience exhibit strong adaptive capabilities and a high capacity to absorb and effectively utilize coping strategies.Individuals with positive emotions in their learning endeavors employ various effective strategies to augment their learning enthusiasm and participation. A survey conducted by Alazemi et al.[27] on high school students found that a higher level of academic psychological resilience among students corresponds to stronger self-efficacy, whereas lower psychological resilience corresponds to lower self-efficacy. The study results also signify a close relationship between academic self-efficacy, as an expression of self-efficacy in learning, and psychological resilience. Social Cognitive Theory emphasizes that individuals with self-efficacy beliefs possess stronger convictions in successfully completing tasks, set more challenging goals, and, when facing difficulties, invest more energy and perseverance in coping. Wicaksono et al.[28], in their study on second language learning, identified a close correlation between self-efficacy, perseverance, academic resilience, and academic demotivation. Self-efficacy and perseverance enable learners to cultivate more positive expectations for learning outcomes in the process of second language acquisition, enhance academic resilience, and thereby sustain more efficient learning engagement in the long run.Research by Shao and Kang [29] and other scholars indicates a close relationship among academic psychological resilience, self-efficacy, and learning engagement. Students with academic psychological resilience, despite encountering challenges, frequently possess strong confidence in successfully completing learning tasks and believe in their ability to do so. Consequently, they manifest elevated levels of learning engagement. Rajan et al.[30], in a survey of 155 high school students in India, identified significant gender differences in academic resilience, revealing a positive correlation between high school students' academic resilience and self-efficacy. Based on these studies, the relationship among psychological resilience, academic self-efficacy, and learning engagement is intimate. Therefore, this study posits Hypothesis 2: Psychological resilience plays a mediating role between academic self-efficacy and learning engagement.

1. **The mediating role of professional commitment in the relationship between academic self-efficacy and learning engagement**

Professional commitment reflects an individual's attitude and behavior toward their chosen major, indicating their identification with the major and willingness to invest time and effort in the field of study[31]. It is an expression of an individual's affection and loyalty to their chosen major. Professional commitment serves as a crucial indicator for comprehending the extent of students' engagement in their major studies. Previous studies have already shown a significant correlation between professional commitment and learning engagement. Chen[32] conducted a survey with 750 university students majoring in preschool education, employing a questionnaire to examine the relationship between their satisfaction with learning, professional commitment, and learning engagement. The results revealed that students majoring in preschool education demonstrated a moderate level of professional commitment, while achieving high scores in learning engagement. A significant positive correlation existed between learning engagement and professional commitment.Research indicates a close relationship between self-efficacy and professional commitment, particularly with emotional commitment. Tsai et al.'s[33] study suggests that a heightened level of self-efficacy positively influences emotional commitment. This positive effect arises because individuals with elevated self-efficacy are more predisposed to embrace the goals and values of the organization compared to those with lower self-efficacy. Orgambídez et al.'s[34] research confirms the intimate relationship between job involvement, self-efficacy, and affective organizational commitment. Individuals with strong self-efficacy are more emotionally receptive to their workplace and are more willing to invest additional energy in their work. Based on this, Hypothesis 3 posits that professional commitment plays a mediating role between academic self-efficacy and learning engagement.

**5.Gender as a moderating factor in the relationship between professional commitment and learning engagement**

Gender is a crucial demographic variable that impacts learning engagement. Owing to the distinct cognitive structures of male and female brains, male and female students exhibit different preferences in cognitive engagement strategies. In comparison to females, males possess stronger information processing abilities and more effective metacognitive monitoring and regulatory strategies. Females concentrate more on utilizing external learning aids and engaging in cognitive strategy learning[35]. Secondly, gender differences exist in factors influencing learning engagement. Gender differentiation theory suggests that, due to the physiological differentiation of gender, individuals gradually develop gender role concepts in the process of social construction.This process implies the ongoing development of individuals and the progression of the socialization process. Individuals of different genders engage in professional learning uniquely, adjusting their expectations of the major based on their understanding formed through learning. The level of professional commitment derived from this process is also diverse, resulting in varied levels of learning engagement[32]. For example, males are more suited to majors that cultivate hands-on skills and problem-solving abilities, leading to more proactive and interactive learning behaviors in these majors. Females, conversely, prefer majors that cultivate reading skills and critical thinking abilities, resulting in higher levels of learning engagement in these majors. From this, it can be inferred that gender differences may exist in how professional commitment impacts college students' learning engagement behaviors. Based on this, Hypothesis 4 posits that gender can moderate the relationship between professional commitment and learning engagement.

In summary, this study integrates social cognitive theory and endeavors to formulate a moderated parallel mediation model. It explores how college students' academic self-efficacy influences learning engagement behavior through the parallel mediating effects of psychological resilience and professional commitment, emphasizing the moderating role of gender. The objective is to offer insights for improving college students' learning participation.


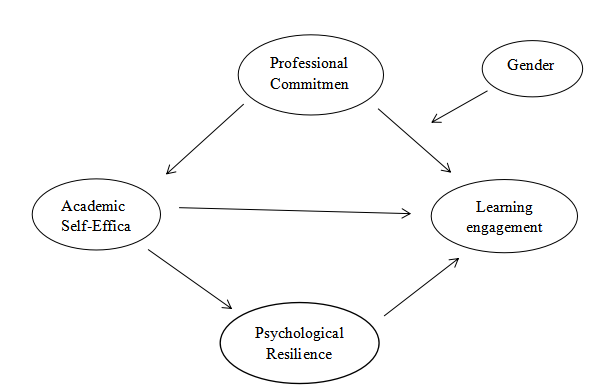


Figure 1: Diagram of the model

**Subject**

Recruited through a cluster random sampling method, participants comprised undergraduate students from a university in Henan, China, spanning from freshmen to seniors. We employed the anonymous survey platform "Wenjuanxing" to collect data, garnering a total of 1187 responses. Following the exclusion of incomplete or insincere responses, we obtained 1032 valid questionnaires. Participants included 376 freshmen (36.4%), 273 sophomores (26.5%), 263 juniors (25.5%), and 120 seniors (11.6%). Among the participants, there were 479 males (46.4%) and 553 females (53.6%). The sample comprised 220 student cadres (21.3%) and 812 non-cadres (78.7%). Additionally, there were 148 only children (14.3%) and 884 non-only children (85.7%). Regarding major selection, 732 participants (70.9%) autonomously chose their majors during the college entrance examination, 107 participants (10.4%) followed their parents' and others' wishes, and 193 participants (18.7%) adjusted their majors based on arrangements.

The study took place from March to September 2023. The research received approval from the Academic Committee of Huanghuai University and was administered using the online survey platform "Wenjuanxing," with participants collectively tested by class. Informed consent was secured from all participants before the testing.

**Psychological Resilience**

In this study, the Chinese version of the Resilience Scale (CD-RISC) [36], translated and revised by Chinese scholars Yu and Zhang, was utilized. Developed by American psychologists Connor and Davidson in 2003, CD-RISC comprises three dimensions: self-improvement, toughness, and optimism, encompassing a total of 25 items. It employs a Likert 5-point rating scale, where scores from "never" to "almost always" are recorded as 0-4 points. The total score ranges from 0 to 100, with a higher score indicating a better level of psychological resilience. The Cronbach's α coefficient for the Chinese version of CD-RISC is 0.916, while in this study, it is 0.963.

**Academic Self-Efficacy**

In this study, the "Academic Self-Efficacy Scale" [37], developed by Liang Yusong in 2004, was employed. The scale comprises 22 items, encompassing two dimensions: self-efficacy for learning ability and self-efficacy for learning behavior. The scale employs a 5-level rating standard, where scores from "strongly disagree" to "strongly agree" are recorded as 1-5 points. Reverse scoring is applied to questions 14, 16, 17, and 20, whereas other questions are scored positively. A higher questionnaire score signifies a higher level of academic self-efficacy in students. In this study, the Cronbach's α coefficient is 0.915.

**Learning Engagement**

The College Student Learning Engagement Questionnaire is utilized to assess the level of learning engagement[38]. The questionnaire comprises 20 items, encompassing three dimensions: behavioral engagement, cognitive engagement, and emotional engagement. A five-point scoring system is employed. A higher score signifies a higher level of learning engagement for the participants. The Cronbach's α coefficients for the College Student Learning Engagement Questionnaire and the three sub-scales (behavioral engagement, cognitive engagement, and emotional engagement) are 0.918, 0.825, 0.858, and 0.858, respectively. The Cronbach's α coefficient for the entire scale is 0.918, indicating good reliability. In this study, the Cronbach's α coefficient is 0.969.

**Professional Commitment**

The "College Students' Professional Commitment Scale"[39], developed by Lian Rong and others, is employed in this study. The scale comprises 27 items, organized into dimensions such as affective commitment, continuance commitment, normative commitment, and ideal commitment. Scores are calculated using a Likert 5-point scoring method, where responses range from "completely disagree" to "completely agree" (scored 1-5). Items 6, 8, and 12 are reverse-scored. A higher score signifies a higher level of professional commitment. According to Lian Rong et al.'s research, the internal consistency Cronbach's α coefficient of the scale is 0.92, while in this study, the Cronbach's α coefficient is 0.955.

**Common Method Bias Test**

The data in this study are all derived from self-reported measures by the participants, which may introduce common method bias. Therefore, Harman's single-factor test was conducted to examine potential bias. The results indicate that there are 11 factors with eigenvalues greater than 1, and the first factor explains 29.766% of the variance, falling below the critical standard of 40%[40]. Thus, there is no substantial evidence of common method bias in this study.

**Correlation analysis**

| Variable | 1 | 2 | 3 | 4 | 5 | 6 |
| --- | --- | --- | --- | --- | --- | --- |
| 1 Grade  2 Gender  3 AS  4 PR  5 PC  6.LE  M  SD | 1  -0.002  0.101^**^  0.073^*^  0.003  0.056  2.12  1.04 | 1  -0.039  0.015  -0.038  -0.065^*^  1.54  0.50 | 1  0.340^**^  0.227^**^  0.577^**^  3.42  0.50 | 1  0.370^**^  0.352^**^  3.45  0.64 | 1  0.320^**^  3.65  0.68 | 1  3.55  0.55 |

Table 1 :Descriptive statistics and correlation analysis of variables (n=1156)

M = mean; SD, standard deviation; N = 1072;AS,Academic Self-Efficacy ;PR,Psychological Resilience;PC,Professional Commitment;LE, Learning engagement; *p < 0.05. **p < 0.01. ***p < 0.001.

The results in Table 1 indicate a positive correlation between academic self-efficacy and psychological resilience, professional commitment, and learning engagement. There is a positive correlation between psychological resilience and professional commitment, as well as learning engagement. Professional commitment is positively correlated with learning engagement.


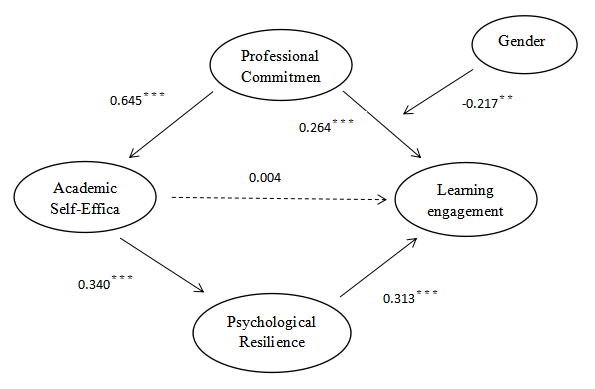


|  | Influence path | Effect | 95%CI | Relative mediating effect(%) |
| --- | --- | --- | --- | --- |
| Indirect effect | PR | 0.136 | [0.094 0.183] | 44.30% |
|  | PC | 0.170 | [0.104 0.234] | 55.37% |
| Total indirect effect |  | 0.307 | [0.232 0.379] | 99.67% |

Table 3: Results with Moderated Mediation Effects

The study applied model 4 in the SPSS macro PROCESS developed by Hayes [41] to examine the mediating effects of psychological resilience and professional commitment between academic self-efficacy and learning engagement. The results, as presented in Table 2 and Figure 2, indicate that, while controlling for variables such as gender, grade, leadership role, only child status, and hometown, academic self-efficacy positively predicts psychological resilience (β= 0.436, p＜0.001) and professional commitment (β= 0.640, p＜0.001). Both psychological resilience and professional commitment positively predict learning engagement (β= 0.312, p＜0.001; β= 0.263, p＜0.001). However, the direct predictive effect of academic self-efficacy on learning engagement becomes non-significant (β=0.001, p=0.976). This implies that the direct effect of academic self-efficacy on learning engagement is not significant, and professional commitment and psychological resilience fully mediate the relationship between academic self-efficacy and learning engagement.

The analysis results reveal that the mediating effect value for the path academic self-efficacy → psychological resilience → learning engagement is 0.136, and for the path academic self-efficacy → professional commitment → learning engagement is 0.170. The 95% confidence interval of the effect values does not include 0, indicating that psychological resilience and professional commitment exhibit significant mediating effects between college students' academic self-efficacy and learning engagement. Psychological resilience and professional commitment fully mediate the impact of college students' academic self-efficacy on learning engagement.

To further investigate the reasons behind gender disparities in professional commitment, Model 14 was employed to test the moderating role of gender in the original parallel mediation model. The results, as presented in Table 3, indicate that the significance of the original paths is consistent with previous observations. Gender exhibits a significant moderating effect on the latter segment of professional commitment mediation (β=-0.217, p＜0.01), while the moderating effects on the initial part of professional commitment and both segments of psychological resilience mediation are not statistically significant.

| Regression equation | Fit index | Significance of regression coefficient | | | | |
| --- | --- | --- | --- | --- | --- | --- |
| Outcome variable | Predictor  variable | R | R^2^ | F | β | t |
| Learning engagement |  | 0.341 | 0.117 | 16.877^＊＊＊^ |  |  |
|  | Gender |  |  |  | -0.035 | -0.859 |
|  | birthplace |  |  |  | 0.062 | 1.287 |
|  | Only child or not |  |  |  | 0.107 | 1.739 |
|  | Volunteer choice |  |  |  | 0.000 | 0.003 |
|  | Class post |  |  |  | -0.022 | -0.449 |
|  | Gender×Professional Commitment |  |  |  | -0.217 | -2.999^＊＊^ |

Table 3: Results with Moderated Mediation Effects

To further explore the moderating effects of professional commitment and gender on learning engagement, we classified professional commitment scores into high and low commitment groups based on one standard deviation above and below the mean. Simple slope analyses were performed on the results, and the corresponding plots are presented in Figure 2.

Figure 2: The mediating role of gender in learning engagement and professional commitment

For female students, the impact of professional commitment on learning engagement exhibits an increasing trend, and the positive predictive effect of professional commitment is significant (β=0.445, t=8.107, p＜0.001). For male students, the predictive effect of professional commitment on learning engagement remains significant (β= 0.228, t=3.843, p＜0.001).

窗体底端

**Discussion**

Integrating social cognitive theory with the framework of psychological resilience, this study explores the impact of academic self-efficacy on learning engagement behavior. The study unveils the mechanisms by which academic self-efficacy influences learning engagement through psychological resilience and professional commitment, along with gender differences. The findings bear both theoretical and practical significance for enhancing the levels of learning engagement among students.

There is a positive correlation between academic self-efficacy and learning engagement, confirming Hypothesis 1. Conversely, students with low academic self-efficacy are more prone to harboring self-doubt and resisting the execution of learning tasks, thereby avoiding academic failure [42]. Maslow's hierarchy of needs theory posits seven hierarchical needs, encompassing physiological needs, safety needs, belongingness and love needs, esteem needs, cognitive needs, aesthetic needs, and self-actualization needs.Maslow argues that the satisfaction of lower-level needs is a prerequisite for achieving self-actualization. This theory suggests that students may lack strong learning motivation when certain levels of needs are not met. When students anticipate positive learning outcomes and believe in their ability to complete learning tasks, the needs for esteem and cognition become exceptionally strong. Once these needs are satisfied, higher-level knowledge-seeking needs emerge, and students continue to choose challenging tasks, willingly investing more resources into the learning process, thus demonstrating higher levels of engagement.Conversely, when students have adverse expectations about learning outcomes and doubt their own capabilities, they may worry about poor grades leading to potential rejection by teachers and peers. This can result in a reluctance to invest excessive energy in learning, potentially leading to learning fatigue and even truancy. Additionally, self-doubt about their learning abilities may gradually lead to learned helplessness and the development of feelings of inferiority. In general, when belongingness and love needs, along with esteem needs, are not met, the motivation for knowledge-seeking tends to weaken.

The study finds that academic self-efficacy influences academic engagement through the mediating role of psychological resilience, confirming Hypothesis 2. Individual factors such as attention, cognition, emotion, and behavior can influence the cultivation of psychological resilience. According to the theory of psychological resilience, the diverse adaptation outcomes of individuals are shaped by the combined influence of environmental factors, internal individual factors, and the interaction between individuals and their environment. The personal factors contributing to psychological resilience consist of cognitive, emotional, physical, mental, and behavioral aspects.Positive emotions can broaden an individual's attention and cognition, as well as continuously build personal positive resources, enhancing behavioral positivity [43]. This study supports this theory, confirming that individuals with high levels of psychological resilience tend to experience more positive emotions and maintain a more optimistic attitude. When facing learning tasks, these individuals are more likely to believe in their abilities and take more proactive actions. Psychological resilience originates from a specific belief system, encompassing one's views on oneself, others, and the goodness and beauty of the world. The belief system is influenced by various factors associated with the individual's life stage [44].

The results of this study indicate that professional commitment acts as a mediator between academic self-efficacy and learning engagement, confirming Hypothesis 3. This aligns with previous research findings. Zhou and Wu[45] demonstrated that professional commitment, academic self-efficacy, teacher support, and learning engagement are pairwise correlated in their study. Professional commitment and academic self-efficacy significantly and positively predict learning engagement. Lu et al.[46], in a survey of over 400 medical students regarding professional commitment, found that self-efficacy affects academic performance through the mediation of professional commitment and learning engagement.In other words, students who assess their learning abilities positively often express strong affection for their chosen profession. They harbor high expectations for the development of their chosen field, willingly adhere to the norms and requirements of their chosen profession, believe in their ability to overcome internal and external challenges in learning, continuously experience and validate their ideas in practical learning, and invest more and more energy into professional learning.

This study found that the moderating effect of gender on the mediating role of academic self-efficacy in the relationship between professional commitment and learning engagement is supported, particularly in the latter part, confirming hypothesis 4. This may be closely related, on the one hand, to traditional gender role positioning or societal expectations. During the process of socialization, individuals acquire gender cognitive schemas, which lead to the manifestation of distinct gender tendencies [47].Females tend to display more emotional and compliant traits. They emotionally endorse their chosen major, unconsciously idealize their academic pursuits, and willingly invest more energy into their studies. In contrast, males tend to exhibit more rational traits. They seek novelty and diversity in their thoughts, exhibit a stronger sense of control, and provide a more comprehensive and objective evaluation of their chosen major. Students are easily influenced by ingrained cognitive schemas and implicit expectations of gender roles, resulting in gender differences in the level of professional identification.

**Conclusion**

This study developed a moderated mediation model to elucidate the relationship between academic self-efficacy and learning engagement. Findings indicated a significant and positive prediction of college students' learning engagement by academic self-efficacy. Both psychological resilience and professional commitment concurrently served as mediating factors between academic self-efficacy and learning engagement. The mediating effect of professional commitment was greater than that of psychological resilience. Academic self-efficacy's predictive role in college students' learning engagement is fully mediated by both psychological resilience and professional commitment. Furthermore, the study revealed gender moderation in the latter part of the pathway for professional commitment. Specifically, females exhibited stronger professional commitment compared to males, leading to elevated levels of learning engagement.

**Research Value and Limitations**

**Research Value**

This study holds both theoretical and practical significance in exploring methods to enhance learning engagement in the post-pandemic era. Firstly, the study aims to construct a mediated model wherein academic self-efficacy influences learning engagement through the mediating factors of psychological resilience and professional commitment. This supplements factors related to the impact of self-efficacy on learning engagement in social cognitive theory, thereby expanding the pathways through which academic self-efficacy affects learning engagement. This offers a theoretical foundation to deepen our understanding of the mechanisms through which academic self-efficacy influences learning engagement. Secondly, the study is of crucial practical significance in enhancing college students' learning engagement. In the post-pandemic era, blended learning has emerged as a trend, and learning engagement stands out as a key factor influencing the quality of online learning.Therefore, effectively enhancing students' learning engagement becomes particularly important. Based on the findings of this study, interventions can be initiated to enhance both psychological resilience and professional commitment levels among college students. Considering the positive effects of professional commitment, one approach is to encourage students to consider their individual characteristics and career preferences when choosing their college majors. Students should thoroughly understand the study content, future employment directions, and prospects of the chosen major to enhance emotional satisfaction with the field of study. Alternatively, after a certain period in the first year, students who cannot adapt to their chosen major should be allowed to make adjustments. School departments can support students by conducting career aptitude tests to help them choose a more suitable major.Leveraging the positive impact of psychological resilience, to enhance college students' learning engagement, integrate positive psychology content like resilience education into classrooms and daily activities, with the goal of elevating students' psychological resilience. Especially for students facing psychological trauma and learning challenges due to COVID-19, implement focused interventions like psychological counseling, group counseling, and therapy to facilitate their swift recovery to the initial level of psychological resilience.

**Limitations**

The study is subject to several limitations. Firstly, reliance on self-reported data introduces inherent reporting biases that are challenging to eliminate. Secondly, the cross-sectional design employed in this study limits a comprehensive examination of causal relationships between variables. Future research could benefit from experimental designs and longitudinal studies to further establish the causal relationships among variables. Thirdly, the study exclusively investigates the influence of professional commitment and psychological resilience on the association between academic self-efficacy and learning engagement. Subsequent studies should explore additional variables with potential mediating or moderating effects, including parental parenting styles, peer support, future orientation, and more.

**Reference**

1.Zheng C. Student Engagement and Academic Performance during the COVID-19 Pandemic: Does a Blended Learning Approach Matter?
International Journal for the Scholarship of Teaching and Learning*.*2023;17(1):1–9. https://doi.org/10.20429/ijsotl.2023.17107

2.Kumar S,Todd G.Effectiveness of online learning interventions on student engagement and academic performance amongst first-year students in allied health disciplines: A systematic review of the literature. Focus Health Prof Ed.2022;23(3);36–55. https://doi.org/10.11157/fohpe.v23i3.430

3.Rashid T, Asghar HM. Technology use, self-directed learning, student engagement and academic performance: Examining the interrelations. Comput Hum Behav*.*2016;63; 604–612. https://doi.org/10.1016/j.chb.2016.05.084

4.Sahni J. Is Learning Analytics the Future of Online Education?: Assessing Student Engagement and Academic Performance in the Online Learning Environment.
Int J Emerg Technol*.* 2023;18(2);33–49. https://doi.org/10.3991/ijet.v18i02.32167

5.Bayoumy HMM, Alsayed S. Investigating Relationship of Perceived Learning Engagement, Motivation, and Academic Performance Among Nursing Students: A Multisite Study. Adv Med Educ Pract.2021;12;351–369. https://doi.org/10.2147/AMEP.S272745

6.Fredricks JA, Blumenfeld PC, Paris AH. School Engagement: Potential of the Concept, State of the Evidence. Rev Educ Res*.* 2004;74; 59–109. https://doi.org/10.3102/00346543074001059

7.Schaufeli WB, Martínez IM, Pinto AM, Salanova M, Bakker AB.Burnout and Engagement in University Students: A Cross-National Study. J Cross Cult Psychol*.*2002; 33(5);464–481. https://doi.org/10.1177/0022022102033005003

8.Bandura A. On the Functional Properties of Perceived Self-Efficacy Revisited. J Manage. 2012;38(1);9–44. https://doi.org/10.1177/0149206311410606

9.Hartley MT.Examining the Relationships Between Resilience, Mental Health, and Academic Persistence in Undergraduate College Students. J Am Coll Health*.* 2011;59(7); 596–604. https://doi.org/10.1080/07448481.2010.515632

10.Smith BW, Dalen J, Wiggins K, Tooley E, Christopher P, Bernard J.The brief resilience scale: Assessing the ability to bounce back. *International Journal of Behavioral Medicine.* 2008 ; 15(3);194–200. https://doi.org/10.1080/10705500802222972

11..Zeng G, Hou H, Peng K.Effect of Growth Mindset on School Engagement and Psychological Well-Being of Chinese Primary and Middle School Students: The Mediating Role of Resilience.Front Psychol. 2016;7;1664-1078. https://doi.org/10.3389/fpsyg.2016.01873

12.Zhao H, Xiong J, Zhang Z, Qi C.Growth mindset and college Students' learning engagement during the COVID-19 pandemic: A serial mediation model. Front Psychol. 2021;12; 1664-1078.

https:// doi.org/10.3389/fpsyg.2021.621094

1. Ye JR, Wu YF, Nong W,Wu YT, Ye JN, Sun Y. The Association of Short-Video Problematic Use, Learning Engagement, and Perceived Learning Ineffectiveness among Chinese Vocational Students. Healthcare ;2023;11(2);161. https://doi.org/10.3390/healthcare11020161

14.Bandura A. Social Foundations of Thought and Action: A Social Cognitive Theory.Upper Saddle River;Prentice-Hall Press;1986 .

15.Bandura A.Self-Efficacy: The Exercise of Control.New York；W.H. Freeman & Company；1997.

16.You W.Research on the Relationship between Learning Engagement and Learning Completion of Online Learning Students. Int J Emerg Technol.2022; 17(1);102–117. https://doi.org/10.3991/ijet.v17i01.28545

17.Namaziandost E, Heydarnejad T, Saeedian S.Language Teacher Professional Identity: The Mediator Role of L2 Grit, Critical Thinking, Resilience, and Self-efficacy Beliefs . Iranian Journal of Applied Language Studies*.*2023; 14(2);107-130. https://doi.org/10.22111/IJALS.2022.7486

18.Xie D, Xie Z. Effects of Undergraduates’ Academic Self-Efficacy on Their Academic Help-Seeking Behaviors: The Mediating Effect of Professional Commitment and the Moderating Effect of Gender.J Coll Student Dev. 2019;60(3); 365–371.https://doi.org/10.1353/csd.2019.0035

19.Ahern NR,Norris AE .Examining factors that increase and decrease stress in adolescent community college students.J Pediatr Nurs.2011;.26(6);530-540.https://doi.org/530-540.10.1016/j.pedn.2010.07.011

20.Cooper AL, Brown JA, Rees CS, Leslie GD.Nurse resilience: A concept analysis. Int J Ment Health Nu , 2020; 29(4);553-575.https://doi.org/10.1111/inm.12721

21.Kumpfer KL.Factors and Processes Contributing to Resilience.2002 ; in: Glantz MD., Johnson JL. Resilience and Development, Longitudinal Research in the Social and Behavioral Sciences: An Interdisciplinary Series. Kluwer Academic Publishers, Boston, 179–224. https://doi.org/10.1007/0-306-47167-1_9

22.Luthar SS, Cicchetti D, Becker B. The construct of resilience: a critical evaluation and guidelines for future work. Child Dev,2000;71(3);543–562. <https://doi.org/10.1111/1467-8624.00164>

23.Cheung VHM, Chan CY, Au, RKC. The influence of resilience and coping strategies on suicidal ideation among Chinese undergraduate freshmen in Hong Kong. Asia-Pac Psychiat .2019;11(2);1758-5864. https://doi.org/10.1111/appy.12339

24.Gillespie BM, Chaboyer W, Wallis M. Development of a theoretically derived model of resilience through concept analysis*.*Contemp Nurse.2007;25(1-2);124-135https://doi.org/10.5172/conu.2007.25.1-2.124

25.Celik DA, Cetin F, Tutkun E. The role of proximal and distal resilience factors and locus of control in understanding hpe, self-esteem and academic achievement among Turkish pre-adolescents.Curr Psychol. 2015;34(2); 321- 345.

26.Leontopoulou.S. Resilience of Greek Youth at an Educational Transition Point: The Role of Locus of Control and Coping Strategies as Resources.Soc Indic Res .2006;76(1); 95–126. https://doi.org/10.1007/s11205-005-4858-3

27.Alazemi AFT, Jember B, Al-Rashidi AH. How to decrease Test Anxiety: a focus on Academic Emotion Regulation, L2 grit, resilience, and self-assessment. Lang Test Asia*.*2023;13(1); 1-17. https://doi.org/10.1186/s40468-023-00241-5

28.Wicaksono BH, Ismail SM, Sultanova SA, Abeba D.I like language assessment: EFL learners’ voices about self-assessment, self-efficacy, grit tendencies, academic resilience, and academic demotivation in online instruction. Lang Test Asia. 2023; 13(1);1-18. https://doi.org/10.1186/s40468-023-00252-2

29..Shao Y, Kang S. The association between peer relationship and learning engagement among adolescents: The chain mediating roles of self-efficacy and academic resilience. Front Psychol.2022; 13; 938756. https://doi.org/10.3389/fpsyg.2022.938756

30.Rajan SK, Harifa PR, Pienyu R. Academic resilience, locus of control, academic engagement and self-efficacy among the school children.Indian J Posit Psychol*.*2017; 8(4); 507–511.

31.Lian R, Yang LX, Wu LH. Relationship between professional commitment and learning burnout of undergraduates and scales developing. J Psychol*.*2005;37(05);632–636. https://kns.cnki.net/kcms/detail/detail.aspx?FileName= XLXB200505008&DbName=CJFQ2005

32.Chen M.Effect of Professional Satisfaction on Learning Engagement in Undergraduates Major in Preschool Education: Mediating Role of Professional Commitment. Psychol, 2018;9(8);2250–2260. https://doi.org/10.4236/psych.2018.98128

33.Tsai CW, Tsai SH, Chen YY, Lee WL. A study of nursing competency, career self-efficacy and professional commitment among nurses in Taiwan. Contemp Nurse*.*2014; 49(1);96–102. https://doi.org/10.1080/10376178.2014.11081959

34.Orgambídez A, BorregoY, Vázquez‐Aguado O.Self‐efficacy and organizational commitment among Spanish nurses: the role of work engagement. Int Nurs Rev.2019;66(3). 381–388. https://doi.org/10.1111/inr.12526

35.Liu R D. On the Essence of Learning Strategies .*Acta Psychologica Sinica.* 1997;179–181. https://doi.org/10.16719/j.cnki.1671-6981.1997.02.024

36.Yu XN,Zhang JX.A Comparison between the Chinese Version of Ego-Resiliency Scale and Connor-Davidson Resilience Scale[ J].Acta Psychol Sin.2007;169 (5): 1169-1171. https://doi.org/10.16719/j.cnki.1671-6981.2007.05.035

37.Liang SY. Study On Achievement Goals、Attribution Styles and Academic Self- efficacy of Collage Students.(master’s thesis). Central China Normal University;2000.

38.Ni KX.Study on the relationship between college students' learning engagement and subjective well-being -- a case study of six universities in chengdu. (master’s thesis).Chengdu University of Technology;2020. https://doi.org/10.26986/d.cnki.gcdlc.2020.001297

39.Lian R,Yang LX, Wu LH.Relationship between Professional Commitment andLesrning Burnout of Undergraduates and Scales Developing .Acta Psychol Sin.2005;37(5);632-636.

40.Zhou H, Long L R. Statistical test and control of common method deviation. Advances in Psychological Science,2004; 12(6);942-942.

41.Hayes AF. An Index and Test of Linear Moderated Mediation. Multivar Behav Res .2015;50(1); 1–22. https://doi.org/10.1080/00273171.2014.962683

42.Allari RS, Atout M.Hasan AAThe value of caring behavior and its impact on students’ self‐efficacy: Perceptions of undergraduate nursing students. Nurs Forum*.*2020; 55(2; 259–266. https://doi.org/10.1111/nuf.12424

43.Chmitorz A, Kunzler A, Helmreich I, Tüscher O, Kalisch R, Kubiak T, Wessa M, LiebK. Intervention studies to foster resilience – A systematic review and proposal for a resilience framework in future intervention studies. Clin Psychol Rev*,* 2018;59;78–100. https://doi.org/10.1016/j.cpr.2017.11.002

44.Jew C , Green K, Kroger J. Development and Validation of a Measure of Resiliency. Meas Eval Couns Dev.1999; 32; 75–89. https://doi.org/10.1080/07481756.1999.12068973

45.Parnikh H, Torabizadeh C, Kalyani MN , Soltanian M . A Study of the Relationship between Professional Communication and Professional Commitment in Operating Room Nurses. Nurs Res Pract.2022;2(6);57-68.https://doi.org/10.1155/2022/5871846

46.Lu Y,Tong K,Wen MG,Gong YY, Zhuang D,Zhu HY. Professional commitment of eight-year medical doctoral degree program students in China: the mediating role of self-efficacy, learning engagement, and academic performance. Bmc Med Educ.2023;(A [preprint](https://www.researchsquare.com/researchers/preprints)). https://doi.org/10.21203/rs.3.rs-3426236/v1

47.Skaar NR, Christ TJ, Jacobucci R. Measuring Adolescent Prosocial and Health Risk Behavior in Schools: Initial Development of a Screening Measure. Sch Ment Health*,*2014; 6(2); 137–149. https://doi.org/10.1007/s12310-014-9123-y
