# Supplementary material for: The relationship between college students’ learning engagement and academic self-efficacy: a moderated mediation model
Source: Front Psychol. 2024 Sep 3;15:1425172. doi: 10.3389/fpsyg.2024.1425172 (PMC11407112; doi:10.3389/fpsyg.2024.1425172)
Supplement: Supplementary file 1 [file Data_Sheet_1.zip › supplementary materials/manuscript/English article/2024.2.28Aricle.docx]

Relationship Between College Students’ Learning Engagement and Academic Self-Efficacy: A Moderated Mediation Model

First Author^1^, Second Author^2*^, Third Author^1,2^

^1^Laboratory X, Institute X, Department X, Organization X, City X, State XX (only USA, Canada and Australia), Country

^2^Laboratory X, Institute X, Department X, Organization X, City X, State XX (only USA, Canada and Australia), Country

*** Correspondence:**Corresponding Author
email@uni.edu

Keywords: learning engagement, academic self-efficacy, professional commitment, psychological resilience.

Abstract

**Introduction:** Despite the return of college students to campus in the post-pandemic era, the profound impact of coronavirus disease (COVID-19) on learning approaches persists. Drawing on the social cognitive theory, this study investigated the interconnections between academic self-efficacy, professional commitment, psychological resilience, and learning engagement among college students in the post-pandemic era. This study explored the influence of academic self-efficacy on learning engagement, with gender as a moderating variable and psychological resilience and professional commitment as mediating variables.

**Methods:** We conducted a survey with 1032 college students in Henan Province, China, utilizing the Psychological Resilience Scale, Academic Self-Efficacy Scale, College Student Learning Engagement Questionnaire, and College Student Professional Commitment Scale. SPSS and the Process plugin were used to assess mediating and moderating effects.

**Results:** The results revealed a significant positive correlation between academic self-efficacy and learning engagement. The positive influence of academic self-efficacy on learning engagement was fully mediated by the parallel effects of psychological resilience and professional commitment, with the mediating effect of professional commitment surpassing that of psychological resilience. Subsequent analyses indicated that gender moderated the mediating effect of professional commitment, with female students demonstrating stronger perceptions of professional commitment associated with elevated levels of learning engagement. Gender did not exhibit a significant moderating effect on psychological resilience.

**Conclusions:**  College students’ academic self-efficacy, professional commitment, and psychological resilience must be addressed to enhance their learning engagement.

# Introduction

Educators are concerned with the level of learners’ engagement (Zheng, 2023). In the post-pandemic era, effective implementation of measures to enhance learning engagement among college students is a concern for many countries. Research suggests that effective learning hinges on learners themselves (Kumar & Todd, 2022). Effective learning necessitates students’ active participation, the internalization of acquired knowledge, and the formation of their own learning experiences (Rashid & Asghar, 2016). Learning engagement is a crucial factor that influences students’ academic performance (Sahni, 2023). An increasing number of countries are linking the level of learning engagement with academic performance, reward and punishment systems, and dropout and graduation rates.

Learning engagement serves as a crucial predictor of the quality of learning (Bayoumy & Alsayed, 2021). During the pandemic, college students predominantly participated in home-based learning through the Internet, resulting in a significant shift in the learning mode from traditional face-to-face collective learning in the pre-pandemic period to non-contact individual learning. At the conclusion of the pandemic, the students returned to the classroom for face-to-face learning. However, influenced by the learning mode during the pandemic, they demonstrated low learning initiative and diminished levels of learning engagement. Therefore, exploring strategies to enhance learning engagement among college students in the post-pandemic era is imperative.

Learning engagement encompasses time, energy, persistent and vigorous emotional states, and cognitive states invested by students in the learning process (Fredricks et al., 2004; Schaufeli et al., 2002). Various theories elucidate the process of learning engagement, including social cognitive and self-determination theories. This study was based on the social cognitive theory, which posits that individual behavior is influenced by social environmental and personal factors. Self-efficacy is a pivotal concept in social cognitive theory, which posits that self-efficacy is shaped by the environment and influences cognitive processes. Heightened self-efficacy fosters individual cognitive development, thereby enhancing academic behavior; moreover, it influences individual behavior (Bandura, 2012). Individuals with high self-efficacy tend to select challenging academic tasks and invest effort in them. Moreover, when they encounter significant setbacks, they recover swiftly and pursue their goals. Prior research has substantiated the social cognitive theory and identified a close relationship between learning engagement, psychological resilience (Hartley, 2011; Smith et al., 2008; Zeng et al., 2016 ; Zhao et al., 2021), and perceived learning ineffectiveness (Ye, 2023).

窗体顶端

窗体底端

Self-efficacy pertains to an individual’s confidence and feelings regarding the organization and the execution of a specific task (Bandura, 1986; Bandura, 1997). Bandura initially proposed self-efficacy and defined it as a crucial psychological factor that significantly influences an individual’s behavior and performance. Self-efficacy comprises two components: efficacy and outcome expectations (You, 2022). General self-efficacy is a comprehensive concept. Since its introduction, diverse fields have undertaken extensive research, resulting in the development of derivative concepts, such as academic and organizational self-efficacy.

Academic self-efficacy involves learners’ self-assessment of their learning abilities. Learners exhibit confidence and a sense of competence in organizing and executing specific learning tasks, leading to a successful understanding of learning materials (Bandura, 1997). As a significant predictive factor in learning, it profoundly influences students’ learning behavior and performance, thereby significantly impacting their level of learning engagement. Numerous studies have demonstrated a positive correlation between academic self-efficacy and learning engagement among college students. Individuals with robust academic self-efficacy showed heightened confidence in completing learning tasks and demonstrated elevated levels of engagement in their studies. Conversely, students with lower academic self-efficacy may experience heightened feelings of helplessness, encounter increased negative emotions, and exhibit reduced participation in their studies (Namaziandost et al., 2023). Academic self-efficacy motivates learners to adopt methods that align with their goals, thereby exerting a substantial influence on the completion of learning tasks. Individuals with robust academic self-efficacy possess a solid cognitive understanding of the learning process and attribute a lack of success to insufficient effort rather than a lack of ability. Students’ academic self-efficacy and learning engagement are intricately intertwined (Xie & Xie, 2019). Therefore, we proposed the following hypothesis:

Hypothesis 1: Academic self-efficacy positively predicts learning engagement.

Psychological resilience is a crucial capability that enables individuals to enhance their capacity to cope with difficulties and respond effectively to sources of stress when confronted with challenges (Ahern & Norris, 2011; Cooper et al., 2020). It is the ability of an individual to maintain a positive adaptive state or “bounce back” to normal life when facing adversity, trauma, misfortune, or significant stressors (Kumpfer, 2002). The psychological resilience framework posits that individuals generate three adaptive outcomes when dealing with stress: an increase in resilience levels, maintaining the original level of resilience, and a decrease in resilience levels after experiencing the shock of stress. The emergence of various adaptive outcomes is influenced by the environment, individual factors, and interactions between individuals and their environment (Luthar et al., 2000).This theory proposes that psychological resilience is dynamic and malleable and plays a crucial protective role in psychological development (Cheung et al., 2019). Psychological resilience is not an inherent personality trait; rather, it continuously develops throughout an individual’s entire life course and is influenced by the surrounding living environment (Gillespie et al., 2007; Celik et al., 2015). Leontopoulou (2006) found that both positive and avoidance coping strategies significantly influenced psychological resilience even in the face of adversity. Individuals with robust psychological resilience exhibit strong adaptive capabilities and a high capacity to absorb and utilize coping strategies. Individuals who experience positive emotions during learning employ various effective strategies to augment their enthusiasm and engagement. Alazemi et al. (2023) discovered that high school students’ academic psychological resilience was positively associated with their self-efficacy.

The social cognitive theory emphasizes that individuals with strong self-efficacy possess strong convictions of successfully completing tasks, set challenging goals, and invest energy and perseverance in coping when facing difficulties. Wicaksono et al. (2023) identified a close correlation between self-efficacy, perseverance, academic resilience, and academic demotivation among second language learners. Self-efficacy and perseverance enable learners to cultivate positive expectations for learning outcomes in the process of second language acquisition, enhance academic resilience, and sustain efficient learning engagement in the long run. Shao and Kang (2022) revealed close relationships among academic psychological resilience, self-efficacy, and learning engagement. Despite encountering challenges, students with academic psychological resilience frequently possess strong confidence in successfully completing learning tasks and believe in their ability to do so. Consequently, they exhibit elevated levels of learning engagement. Rajan et al. (2017) identified significant gender differences in academic resilience among 155 high school students in India, revealing a positive correlation between academic resilience and self-efficacy. These studies indicate close relationships among psychological resilience, academic self-efficacy, and learning engagement. Therefore, this study proposed the following hypothesis:

Hypothesis 2: Psychological resilience mediates the relationship between academic self-efficacy and learning engagement.

Professional commitment reflects an individual’s attitude and behavior toward their chosen major, indicating their identification with the major and willingness to invest time and effort in the field of study (Lian et al., 2005). It is an expression of an individual’s affection for and loyalty toward their chosen major. Professional commitment serves as a crucial indicator for comprehending the extent of student engagement in their majors. Previous studies have demonstrated a significant correlation between professional commitment and learning engagement. Chen (2018) surveyed 750 university students majoring in preschool education to examine the relationship between their satisfaction with learning, professional commitment, and learning engagement and found that the participants demonstrated a moderate level of professional commitment while achieving high scores in learning engagement. A significant positive correlation was observed between learning engagement and professional commitment.

Research indicates a close relationship between self-efficacy and professional commitment, particularly emotional commitment. Tsai et al. (2014) suggested that a heightened level of self-efficacy positively influences emotional commitment. This positive effect arises because individuals with elevated self-efficacy are more predisposed to embrace the goals and values of an organization than those with lower self-efficacy. Orgambídez et al. (2019) confirmed the close relationships among job involvement, self-efficacy, and affective organizational commitment. Individuals with strong self-efficacy are more emotionally receptive to their workplace and more willing to invest additional energy in their work. Therefore, we proposed the following hypothesis:

Hypothesis 3: Professional commitment mediates the relationship between academic self-efficacy and learning engagement.

Gender is a crucial demographic variable affecting learning engagement. Male and female students exhibit different preferences in cognitive engagement strategies due to the distinct cognitive structures of their brains. Men possess stronger information processing abilities and more effective metacognitive monitoring and regulatory strategies than women. Women concentrate more on utilizing external learning aids and engaging in cognitive strategy learning than men (Liu, 1997). Furthermore, gender differences exist in the factors that influence learning engagement. Gender differentiation theory suggests that due to the physiological differentiation of gender, individuals gradually develop gender role concepts in the process of social construction. This process implies the ongoing development of individuals and progression of the socialization process. Individuals of different genders uniquely engage in professional learning, adjusting their expectations of their major based on the understanding formed through learning. The level of professional commitment derived from this process is also diverse, resulting in varying levels of learning engagement (Chen, 2018). For instance, men are more suited to majors that cultivate hands-on skills and problem-solving abilities, leading to more proactive and interactive learning behaviors. Conversely, women prefer majors that cultivate reading and critical thinking abilities, resulting in higher levels of learning engagement. Thus, gender differences may exist in how professional commitment affects college students’ learning engagement behaviors. Therefore, we proposed the following hypothesis:

Hypothesis 4: Gender moderates the relationship between professional commitment and learning engagement.

This study integrated social cognitive theory and formulated a moderated parallel mediation model (Figure 1). This study explored the effect of college students’ academic self-efficacy on learning engagement through the parallel mediating effects of psychological resilience and professional commitment, emphasizing the moderating role of gender. This study aimed to offer insights for improving college students’ participation in learning.

# Materials and Methods

## Participants

This study recruited undergraduate students from a university in Henan, China, ranging from freshmen to seniors, using random cluster sampling. We employed the anonymous survey platform Wenjuanxing to collect data, garnering a total of 1187 responses. After excluding incomplete or insincere responses, we obtained 1032 valid questionnaires. The participants included 376 freshmen (36.4%), 273 sophomores (26.5%), 263 juniors (25.5%), and 120 seniors (11.6%). Among the participants, there were 479 men (46.4%) and 553 women (53.6%). The sample consisted of 220 student cadres (21.3%) and 812 non-cadres (78.7%). Moreover, the sample included 148 only children (14.3%) and 884 non-only children (85.7%). Regarding college major selection, 732 participants (70.9%) autonomously chose their majors during the college entrance examination, 107 (10.4%) followed their parents’ and others’ wishes, and 193 (18.7%) adjusted their majors based on arrangements.

The study was conducted between March and September, 2023. The research was approved by the Academic Committee of Huanghuai University and was administered using the online survey platform Wenjuanxing, with participants collectively tested by class. Informed consent was obtained from all participants before testing.

## Psychological Resilience

We used the Chinese version of the Resilience Scale (CD-RISC) (Yu, 2007) translated and revised by Yu and Zhang. Developed by the American psychologists Connor and Davidson in 2003, the 25-item CD-RISC comprises three dimensions: self-improvement, toughness, and optimism. Responses are rated on a five-point Likert scale (0 = never; 4 = almost always). The total score ranged from 0 to 100, with a higher score indicating a higher level of psychological resilience. The Cronbach’s α coefficient for the Chinese version of CD-RISC was 0.916, and it was 0.963 in this study.

## Academic Self-Efficacy

We used the Academic Self-Efficacy Scale (Liang, 2000), developed by Liang Yusong in 2004. The scale comprises 22 items encompassing two dimensions: self-efficacy for learning ability and self-efficacy for learning behavior. Responses are rated on a five-point Likert scale (1 = strongly disagree; 5 = strongly agree). Reverse scoring was applied to Items 14, 16, 17, and 20, whereas the other items were scored positively. A higher questionnaire score signified a higher level of academic self-efficacy. In this study, the Cronbach’s α coefficient was 0.915.

## Learning Engagement

The College Student Learning Engagement Questionnaire was used to assess the students’ level of learning engagement (Ni, 2020). The questionnaire comprises 20 items encompassing three dimensions: behavioral, cognitive, and emotional engagement. Responses are rated on a five-point Likert scale. A higher score indicated a higher level of learning engagement. The Cronbach’s α coefficients for the College Student Learning Engagement Questionnaire and the three sub-scales (behavioral, cognitive, and emotional engagement) were 0.918, 0.825, 0.858, and 0.858, respectively. In this study, the Cronbach’s α coefficient was 0.969.

## Professional Commitment

The College Students’ Professional Commitment Scale (Lian et al. 2005), developed by Lian Rong et al., was used in this study. The scale comprises 27 items organized into dimensions, such as affective commitment, continuance commitment, normative commitment, and ideal commitment. Responses are rated on a five-point Likert scale (1 = completely disagree; 5 = completely agree). Items 6, 8, and 12 were reverse scored. Higher scores indicated a higher level of professional commitment. According to Lian Rong et al., the internal consistency Cronbach’s α coefficient of the was is 0.92; in this study, the Cronbach’s α coefficient was 0.955.

## Common Method Bias Test

The data in this study were all derived from self-reported measures by the participants, which may have introduced common method bias. Therefore, Harman’s single-factor test was conducted to examine potential bias. The results revealed 11 factors with eigenvalues greater than 1; the first factor explained 29.766% of the variance, falling below the critical standard of 40% (Zhou & Long, 2004). Thus, no substantial evidence of common method bias was found.

# Results

## Correlation Analysis

As shown in Table 1, positive correlations were observed among academic self-efficacy, psychological resilience, professional commitment, and learning engagement. Moreover, positive correlations were observed among psychological resilience, professional commitment, and learning engagement. Professional commitment was positively correlated with learning engagement.

## Parallel Mediation Tests

Model 4 was applied to the SPSS macro PROCESS developed by Hayes (2015) to examine the mediating effects of psychological resilience and professional commitment on the relationship between academic self-efficacy and learning engagement. As shown in Table 2 and Figure 2, after controlling for variables such as gender, grade, leadership role, only child status, and hometown, academic self-efficacy positively predicted psychological resilience (β = 0.436, p < 0.001) and professional commitment (β = 0.640, p < 0.001). Both psychological resilience and professional commitment positively predicted learning engagement (β = 0.312, p < 0.001; β = 0.263, p < 0.001). However, the direct predictive effect of academic self-efficacy on learning engagement was not significant (β = 0.001, p = 0.976). This implied that the direct effect of academic self-efficacy on learning engagement was not significant, and that professional commitment and psychological resilience fully mediated the relationship between academic self-efficacy and learning engagement.

The results revealed that the mediating effect value for the path academic self-efficacy → psychological resilience → learning engagement was 0.136, and for the path academic self-efficacy → professional commitment → learning engagement was 0.170. The 95% confidence interval of the effect values did not include zero, indicating that psychological resilience and professional commitment significantly mediated the relationship between college students’ academic self-efficacy and learning engagement. Psychological resilience and professional commitment fully mediated the impact of academic self-efficacy on learning engagement.

To further investigate the reasons for sex disparities in professional commitment, Model 14 was used to test the moderating role of gender in the original parallel mediation model. As shown in Table 3, the results indicated that the significance of the original paths was consistent with previous observations. Gender exhibited a significant moderating effect on the latter segment of professional commitment mediation (β = -0.217, p < 0.01), whereas the moderating effects on the initial part of professional commitment and both segments of psychological resilience mediation were not statistically significant.

To further explore the moderating effects of professional commitment and gender on learning engagement, we classified professional commitment scores into high- and low-commitment groups based on one standard deviation above and below the mean. Simple slope analyses were performed on the results, and the corresponding plots are shown in Figure 3.

For female students, the impact of professional commitment on learning engagement exhibited an increasing trend, and the positive predictive effect of professional commitment was significant (β = 0.445, t = 8.107, p < 0.001). For male students, the predictive effect of professional commitment on learning engagement remained significant (β = 0.228, t = 3.843, p < 0.001).

# Discussion

By integrating the social cognitive theory with the framework of psychological resilience, this study explored the impact of academic self-efficacy on learning engagement. This study revealed the mechanisms by which academic self-efficacy influences learning engagement through psychological resilience and professional commitment, along with gender differences. These findings have theoretical and practical significance for enhancing the level of learning engagement among students.

A positive correlation was found between academic self-efficacy and learning engagement, supporting Hypothesis 1. Students with low academic self-efficacy are prone to harbor self-doubt and resist the execution of learning tasks, thereby avoiding academic failure (Allari, 2020). Maslow’s hierarchy of needs theory posits seven hierarchical needs: physiological, safety, belongingness and love, esteem, cognitive, aesthetic, and self-actualization. Maslow argues that satisfaction of lower-level needs is a prerequisite for achieving self-actualization. This theory suggests that students may lack strong learning motivation when certain needs are not met. When students anticipate positive learning outcomes and believe in their ability to complete learning tasks, their need for esteem and cognition becomes exceptionally strong. Once these needs are satisfied, higher-level knowledge-seeking needs emerge, and students continue to choose challenging tasks, willingly investing more resources into the learning process, thus demonstrating higher levels of engagement. Conversely, when students have adverse expectations about learning outcomes and doubt their own capabilities, they may worry about poor grades, leading to potential rejection by teachers and peers. This can result in reluctance to invest excessive energy in learning, potentially leading to learning fatigue and even truancy. In addition, self-doubt regarding one’s learning abilities may gradually lead to learned helplessness and feelings of inferiority. When belongingness, love, and esteem needs are not met, motivation for knowledge seeking tends to weaken.

This study found that academic self-efficacy influenced learning engagement through the mediating role of psychological resilience, supporting Hypothesis 2. Individual factors, such as attention, cognition, emotion, and behavior, can influence the cultivation of psychological resilience. According to psychological resilience theory, the diverse adaptation outcomes of individuals are shaped by the combined influence of environmental factors, internal individual factors, and the interaction between individuals and their environment. The personal factors contributing to psychological resilience comprise cognitive, emotional, physical, mental, and behavioral aspects. Positive emotions can broaden an individual’s attention and cognition, as well as continuously build personal positive resources, enhancing behavioral positivity (Chmitorz et al., 2018). The present study supports this theory by confirming that individuals with high levels of psychological resilience tend to experience more positive emotions and maintain more optimistic attitudes. When faced with learning tasks, these individuals are more likely to believe in their abilities and take more proactive actions. Psychological resilience originates from a specific belief system that encompasses one’s views of oneself, others, and the goodness and beauty of the world. This belief system is influenced by various factors associated with an individual’s life stages (Jew et al., 1999).

The results of this study indicated that professional commitment mediated the relationship between academic self-efficacy and learning engagement, supporting Hypothesis 3. This aligns with the findings of previous research. Lu et al. (2023), in a survey of over 400 medical students regarding professional commitment, found that self-efficacy affects academic performance through the mediation of professional commitment and learning engagement. In other words, students who assess their learning abilities positively often express strong affection for their chosen profession. They have high expectations for development in their chosen field, willingly adhere to the norms and requirements of their chosen profession, believe in their ability to overcome internal and external challenges in learning, continuously experience and validate their ideas in practical learning, and invest energy into professional learning.

This study revealed the moderating effect of gender on the mediating role of academic self-efficacy in the relationship between professional commitment and learning engagement, particularly in the latter, supporting Hypothesis 4. This may be closely related to traditional gender role positioning or societal expectations. During the process of socialization, individuals acquire gender-cognitive schemas, which lead to the manifestation of distinct gender tendencies (Skaar et al., 2014). Women tend to display emotional and compliant traits. They emotionally endorse their chosen majors, unconsciously idealize their academic pursuits, and willingly invest more energy in their studies. In contrast, men tend to exhibit rational traits. They seek novelty and diversity in their thoughts, exhibit a strong sense of control, and provide comprehensive and objective evaluations of their chosen majors. Students are easily influenced by their ingrained cognitive schemas and implicit expectations of gender roles, resulting in gender differences in their levels of professional identification.

# Conclusion

This study developed a moderated mediation model to examine the relationship between academic self-efficacy and learning engagement. Our findings indicated that academic self-efficacy was a significant and positive predictor of college students’ learning engagement. Psychological resilience and professional commitment concurrently served as mediating factors in the relationship between academic self-efficacy and learning engagement. The mediating effect of professional commitment was stronger than that of psychological resilience. Academic self-efficacy’s predictive role in college students’ learning engagement was fully mediated by both psychological resilience and professional commitment. Furthermore, the study revealed a gender moderation in the latter part of the pathway for professional commitment. Specifically, women exhibited stronger professional commitment than men, leading to elevated levels of learning engagement.

# Implications and Limitations

## Implications

This study has both theoretical and practical significance, as it explored methods for enhancing learning engagement in the post-pandemic era. First, it aimed to construct a mediated model in which academic self-efficacy influences learning engagement through the mediating factors of psychological resilience and professional commitment. This supplemented the factors related to the impact of self-efficacy on learning engagement in social cognitive theory, thereby expanding the pathways through which academic self-efficacy affects learning engagement. This study offers a theoretical foundation for deepening our understanding of the mechanisms by which academic self-efficacy influences learning engagement. Second, this study is of crucial practical significance for enhancing college students’ learning engagement. In the post-pandemic era, blended learning has emerged as a trend, and learning engagement stands out as a key factor influencing the quality of online learning. Therefore, effectively enhancing students’ learning engagement has become particularly important. Based on these findings, interventions should be initiated to enhance psychological resilience and professional commitment among college students. Considering the positive effects of professional commitment, one approach is to encourage students to consider their individual characteristics and career preferences while choosing a college major. Students should thoroughly understand the study content, future employment directions, and prospects of the chosen major to enhance their emotional satisfaction with their field of study. Alternatively, students who cannot adapt to their chosen majors after a certain period during the first year should be allowed to make adjustments. School departments could support students by conducting career aptitude tests to help them choose a more suitable major. Leveraging the positive impact of psychological resilience, teachers could integrate positive psychology content, such as resilience education, into classrooms and daily activities to enhance college students’ learning engagement and increased their psychological resilience. Especially for students facing psychological trauma and learning challenges due to COVID-19, focused interventions, such as psychological counseling, group counseling, and therapy, are required to facilitate their swift recovery to the initial level of psychological resilience.

## Limitations

This study had several limitations. First, the reliance on self-reported data introduces inherent reporting biases that are challenging to eliminate. Second, the cross-sectional design employed in this study limited the comprehensive examination of the causal relationships between the variables. Future research could benefit from experimental designs and longitudinal studies to further establish causal relationships between variables. Third, this study investigated the influence of professional commitment and psychological resilience on the association between academic self-efficacy and learning engagement. Subsequent studies should explore additional variables with potential mediating or moderating effects, including parenting style, peer support, and future orientation.

# Conflict of Interest

The authors declare that the research was conducted in the absence of any commercial or financial relationships that could be construed as a potential conflict of interest.

# Author Contributions

# Funding

# Acknowledgments

# References

Ahern NR.,&Norris AE .(2011).Examining factors that increase and decrease stress in adolescent community college students.*Journal of pediatric nursing,* 26(6),530-540.https://doi.org/530-540.10.1016/j.pedn.2010.07.011

Alazemi, A.F.T., Jember, B., &Al-Rashidi, A.H.(2023). How to decrease Test Anxiety: a focus on Academic Emotion Regulation, L2 grit, resilience, and self-assessment. *Language Testing in Asia,* 13(1), 1-17. https://doi.org/10.1186/s40468-023-00241-5

Allari, R.S., Atout, M., &Hasan, A.A.(2020) . The value of caring behavior and its impact on students’ self‐efficacy: Perceptions of undergraduate nursing students. *Nursing Forum,* 55(2), 259–266. https://doi.org/10.1111/nuf.12424

Bayoumy,H.M.M.,& Alsayed, S., 2021. Investigating Relationship of Perceived Learning Engagement, Motivation, and Academic Performance Among Nursing Students: A Multisite Study. *Advances in Medical Education and Practice*,12,351–369. https://doi.org/10.2147/AMEP.S272745

Bandura, A., (2012). On the Functional Properties of Perceived Self-Efficacy Revisited. *Journal of Management*, 38(1), 9–44. https://doi.org/10.1177/0149206311410606

Bandura, A. (1986) .Social Foundations ofThought and Action: A Social Cognitive Theory; Prentice-Hall: Englewood Cliffs, NJ, USA

Bandura, A. (1997).Self-Efficacy: The Exercise ofControl; Worth Publishers, Incorporated.New York, NY, USA .

Celik, D. A., Cetin , F., & Tutkun, E. (2015). The role of proximal and distal resilience factors and locus of control in understanding hpe, self-esteem and academic achievement among Turkish pre-adolescents. *Current Psychology,* 34(2), 321- 345

Chen, M., (2018). Effect of Professional Satisfaction on Learning Engagement in Undergraduates Major in Preschool Education: Mediating Role of Professional Commitment. *Psychology*, 9(8), 2250–2260. https://doi.org/10.4236/psych.2018.98128

Chmitorz, A., Kunzler, A., Helmreich, I., Tüscher, O., Kalisch, R., Kubiak, T., Wessa, M.,& Lieb, K., (2018). Intervention studies to foster resilience – A systematic review and proposal for a resilience framework in future intervention studies. *Clinical Psychology Review*, 59, 78–100. https://doi.org/10.1016/j.cpr.2017.11.002

Cheung, V.H.M., Chan, C.Y.,& Au, R.K.C.,(2019) . The influence of resilience and coping strategies on suicidal ideation among Chinese undergraduate freshmen in Hong Kong. *Asia-Pacific Psychiatry* ,11(2), 1758-5864. https://doi.org/10.1111/appy.12339

Cooper, A.L., Brown, J.A., Rees, C.S., & Leslie,G.D.(2020). Nurse resilience: A concept analysis. [*International Journal of Mental Health Nursing*](https://ss.zhizhen.com/nav/mag/info?mags=5f114fc19e3078ecfa78a02f861c384f) *,* 29(4),553-575.https://doi.org/10.1111/inm.12721

Fredricks, J.A., Blumenfeld, P.C., &Paris, A.H., (2004). School Engagement: Potential of the Concept, State of the Evidence. *Review of Educational Research ,*74, 59–109. https://doi.org/10.3102/00346543074001059

Gillespie, B. M., Chaboyer, W., & Wallis, M. (2007).Development of a theoretically derived model of resilience through concept analysis.*Contemporary Nurse*,25(1-2),124-135https://doi.org/10.5172/conu.2007.25.1-2.124

Hartley, M.T.(2011). Examining the Relationships Between Resilience, Mental Health, and Academic Persistence in Undergraduate College Students. *Journal of American College Health,* 59(7), 596–604. https://doi.org/10.1080/07448481.2010.515632

Hayes, A.F.（2015）. An Index and Test of Linear Moderated Mediation. *Multivariate Behavioral Research ,*50(1), 1–22. https://doi.org/10.1080/00273171.2014.962683

Jew, C., Green, K., &Kroger, J. (1999). Development and Validation of a Measure of Resiliency. *Measurement and Evaluation in Counseling and Development,* 32, 75–89. https://doi.org/10.1080/07481756.1999.12068973

Kumpfer, K.L.(2002) . Factors and Processes Contributing to Resilience, in: Glantz, M.D., Johnson, J.L. (Eds.), Resilience and Development, Longitudinal Research in the Social and Behavioral Sciences: An Interdisciplinary Series. Kluwer Academic Publishers, Boston, pp. 179–224. https://doi.org/10.1007/0-306-47167-1_9

Kumar, S., &Todd, G.（2022）. Effectiveness of online learning interventions on student engagement and academic performance amongst first-year students in allied health disciplines: A systematic review of the literature. *Focus on health professional education,*23(3),36–55. https://doi.org/10.11157/fohpe.v23i3.430

Luthar, S.S., Cicchetti, D., &Becker, B.(2000). The construct of resilience: a critical evaluation and guidelines for future work. *Child Development,*71(3), 543–562. https://doi.org/10.1111/1467-8624.00164

Leontopoulou, S.(2006). Resilience of Greek Youth at an Educational Transition Point: The Role of Locus of Control and Coping Strategies as Resources.*Social Indicators Research ,*76(1), 95–126. https://doi.org/10.1007/s11205-005-4858-3

Lu,Y.,Tong,K.,Wen,M.G.,Gong,Y.Y.,Zhuang,D.&Zhu,H.Y.(2023). Professional commitment of eight-year medical doctoral degree program students in China: the mediating role of self-efficacy, learning engagement, and academic performance. *BMC Medical Education*, 2023. https://doi.org/10.21203/rs.3.rs-3426236/v1

Lian, R, .Yang ,L.X., &Wu ,L.H. (2005). Relationship between professional commitment and learning burnout of undergraduates and scales developing. *Acta Psychologica Sinica,*37(05):632–636.

Liu, R. D.(1997) .On the Essence of Learning Strategies .*Journal of Psychological Science.* 179–181. doi:10.16719/j.cnki.1671-6981.1997.02.024

Liang, S.Y.(2000). Study On Achievement Goals、Attribution Styles and Academic Self- efficacy of Collage Students.[master’s thesis]. [Wuhan]: Central China Normal University.

Namaziandost, E., Heydarnejad, T., &Saeedian, S.(2023). Language Teacher Professional Identity: The Mediator Role of L2 Grit, Critical Thinking, Resilience, and Self-efficacy Beliefs .*Iranian Journal of Applied Language Studies,* 14(2), 107-130. https://doi.org/10.22111/IJALS.2022.7486

Ni ,K.X.(2020).Study on the relationship between college students' learning engagement and subjective well-being -- a case study of six universities in chengdu. [master’s thesis].[Chengdu ]: Chengdu University of Technology; doi:10.26986/d.cnki.gcdlc.2020.001297

Orgambídez, A., Borrego, Y., &Vázquez‐Aguado, O.(2019). Self‐efficacy and organizational commitment among Spanish nurses: the role of work engagement. *International Nursing Review ,*66(3), 381–388. https://doi.org/10.1111/inr.12526

Rajan, S.K., Harifa, P.R.,& Pienyu, R.(2017) . Academic resilience, locus of control, academic engagement and self-efficacy among the school children. *Indian Journal of Positive Psychology,* 8(4), 507–511.

Rashid, T., &Asghar, H.M.(2016). Technology use, self-directed learning, student engagement and academic performance: Examining the interrelations. *Computers in Human Behavior,* 63, 604–612. https://doi.org/10.1016/j.chb.2016.05.084

Sahni, J.(2023). Is Learning Analytics the Future of Online Education?: Assessing Student Engagement and Academic Performance in the Online Learning Environment.
*International Journal of Emerging Technologies in Learning,* 18(2), 33–49. https://doi.org/10.3991/ijet.v18i02.32167

Schaufeli, W.B., Martínez, I.M., Pinto, A.M., Salanova, M., &Bakker, A.B.(2002). Burnout and Engagement in University Students: A Cross-National Study. *Journal of Cross-Cultural Psychology* ,33(5), 464–481. https://doi.org/10.1177/0022022102033005003

Smith, B.W., Dalen, J., Wiggins, K., Tooley, E., Christopher, P., &Bernard, J.(2008) . The brief resilience scale: Assessing the ability to bounce back. International *Journal of Behavioral Medicine,*15(3), 194–200. https://doi.org/10.1080/10705500802222972

Shao ,Y.,& Kang, S. (2022). The association between peer relationship and learning engagement among adolescents: The chain mediating roles of self-efficacy and academic resilience. [*Frontiers in Psychology*](https://ss.zhizhen.com/nav/mag/info?mags=a17ad88903f1b8b77fe6480a64eb7efd)*,*13,938756. doi:10.3389/fpsyg.2022.938756

Skaar, N.R., Christ, T.J., &Jacobucci, R.(2014). Measuring Adolescent Prosocial and Health Risk Behavior in Schools: Initial Development of a Screening Measure. *School Mental Health,* 6(2), 137–149. https://doi.org/10.1007/s12310-014-9123-y

Tsai, C.W., Tsai, S.H., Chen, Y.Y., &Lee, W.L.(2014) . A study of nursing competency, career self-efficacy and professional commitment among nurses in Taiwan. *Contemporary Nurse,* 49(1), 96–102. https://doi.org/10.1080/10376178.2014.11081959

Wicaksono, B.H., Ismail, S.M., Sultanova, S.A.,& Abeba, D.(2023). I like language assessment: EFL learners’ voices about self-assessment, self-efficacy, grit tendencies, academic resilience, and academic demotivation in online instruction. *Language Testing in Asia*, 13(1), 1-18.. https://doi.org/10.1186/s40468-023-00252-2

Xie, D., &Xie, Z.(2019). Effects of Undergraduates’ Academic Self-Efficacy on Their Academic Help-Seeking Behaviors: The Mediating Effect of Professional Commitment and the Moderating Effect of Gender. *Journal of College Student Development,* 60(3), 365–371.https://doi.org/10.1353/csd.2019.0035

Yu ,X.N.,& Zhang, J.X.A .(2007).Comparison between the Chinese Version of Ego-Resiliency Scale and Connor-Davidson Resilience Scale.J*ournal of Psychological Science,*169 ,1169-1171. doi:10.16719/j.cnki.1671-6981.2007.05.035

Ye, J.R., Wu ,Y.F, Nong, W.,Wu, Y,T., Ye, J.N., &Sun, Y.(2023).The Association of Short-Video Problematic Use, Learning Engagement, and Perceived Learning Ineffectiveness among Chinese Vocational Students. *Healthcare*.11,161. doi:10.3390/healthcare11020161

You, W.(2022). Research on the Relationship between Learning Engagement and Learning Completion of Online Learning Students. *International Journal of Emerging Technologies in Learning ,*17(1), 102–117. https://doi.org/10.3991/ijet.v17i01.28545

Zeng, G., Hou, H., &Peng, K.(2016) . Effect of Growth Mindset on School Engagement and Psychological Well-Being of Chinese Primary and Middle School Students: The Mediating Role of Resilience.*Frontiers in Psychology,* 7,1664-1078. https://doi.org/10.3389/fpsyg.2016.01873

Zhao, H., Xiong, J., Zhang, Z., & Qi, C. (2021). Growth mindset and college Students' learning engagement during the COVID-19 pandemic: A serial mediation model. *Frontiers in Psychology,* 12, 1664-1078.

https:// doi.org/10.3389/fpsyg.2021.621094

Zheng, C.(2023). Student Engagement and Academic Performance during the COVID-19 Pandemic: Does a Blended Learning Approach Matter?
*International Journal for the Scholarship of Teaching and Learning,* 17(1), 1–9. https://doi.org/10.20429/ijsotl.2023.17107

Zhou, H., & Long, L. R. (2004). Statistical test and control of common method deviation. *Progress in Psychological Science,* 12(6), 942-942.

# Figure Legends

Figure 1. Diagram of the model.

Figure 2. A moderated mediation model

Figure 3. The mediating role of gender in learning engagement and professional commitment

# Tables

Table 1. Descriptive statistics and correlation analysis of variables (n=1156)

| Variable | 1 | 2 | 3 | 4 | 5 | 6 |
| --- | --- | --- | --- | --- | --- | --- |
| 1 Grade  2 Gender  3 AS  4 PR  5 PC  6.LE  M  SD | 1  -0.002  0.101^**^  0.073^*^  0.003  0.056  2.12  1.04 | 1  -0.039  0.015  -0.038  -0.065^*^  1.54  0.50 | 1  0.340^**^  0.227^**^  0.577^**^  3.42  0.50 | 1  0.370^**^  0.352^**^  3.45  0.64 | 1  0.320^**^  3.65  0.68 | 1  3.55  0.55 |

M, mean; SD, standard deviation; AS, academic self-efficacy; PR, psychological resilience; PC, professional commitment; LE, learning engagement. *p < 0.05. **p < 0.01. ***p < 0.001.

Table 2. Results with moderated mediation effects

|  | Influence path | Effect | 95%CI | Relative mediating effect (%) |
| --- | --- | --- | --- | --- |
| Indirect effect | PR | 0.136 | [0.094 0.183] | 44.30% |
|  | PC | 0.170 | [0.104 0.234] | 55.37% |
| Total indirect effect |  | 0.307 | [0.232 0.379] | 99.67% |

CI, confidence interval; PR, psychological resilience; PC, professional commitment.

Table 3. Results with moderated mediation effects

| Regression equation | Fit index | Significance of regression coefficient | | | | |
| --- | --- | --- | --- | --- | --- | --- |
| Outcome variable | Predictor  variable | R | R^2^ | F | β | t |
| Learning engagement |  | 0.341 | 0.117 | 16.877^＊＊＊^ |  |  |
|  | Gender |  |  |  | -0.035 | -0.859 |
|  | birthplace |  |  |  | 0.062 | 1.287 |
|  | Only child or not |  |  |  | 0.107 | 1.739 |
|  | Volunteer choice |  |  |  | 0.000 | 0.003 |
|  | Class post |  |  |  | -0.022 | -0.449 |
|  | Gender × Professional Commitment |  |  |  | -0.217 | -2.999^＊＊^ |

# Data Availability Statement

The datasets [GENERATED/ANALYZED] for this study can be found in the [NAME OF REPOSITORY] [LINK]. Please see the “Availability of data” section of [Materials and data policies in the Author guidelines](https://www.frontiersin.org/guidelines/policies-and-publication-ethics#materials-and-data-policies) for more details.
