# Supplementary material for: The relationship between college students’ learning engagement and academic self-efficacy: a moderated mediation model
Source: Front Psychol. 2024 Sep 3;15:1425172. doi: 10.3389/fpsyg.2024.1425172 (PMC11407112; doi:10.3389/fpsyg.2024.1425172)
Supplement: Supplementary file 1 [file Data_Sheet_1.zip › supplementary materials/manuscript/English article/20240310_Article_Text.docx]

Relationship Between College Students’ Learning Engagement and Academic Self-Efficacy: A Moderated Mediation Model

Yaxing Wang ^1,2^*, Wen Zhang^3^

^1^School of Psychology, Northwest Normal University, Lanzhou, China

^2^Mental Health Service Center, Huanghuai University, Zhumadian, China

^3^Gong Cheng Xun Lian Zhong Xin, Huanghuai University, Zhumadian, China

*** Correspondence:**Yaxing Wang
[wangyxxg@163.c](mailto:email@uni.edu)om

Keywords: learning engagement, academic self-efficacy, professional commitment, psychological resilience.

Abstract

**Introduction:** Despite the return of college students to campus in the post-pandemic era, the profound impact of coronavirus disease (COVID-19) on learning approaches persists. Existing research has explored fewer mechanisms underlying academic self-efficacy and learning engagement. Drawing on the social cognitive theory, this study investigated the interconnections between academic self-efficacy, professional commitment, psychological resilience, and learning engagement among college students in the post-pandemic era. The study explored how academic self-efficacy affects learning engagement with gender as a moderating variable and psychological resilience and professional commitment as mediating variables.

**Methods:** We conducted a survey with 1032 college students in Henan Province, China, utilizing the Psychological Resilience Scale, Academic Self-Efficacy Scale, College Student Learning Engagement Questionnaire, and College Student Professional Commitment Scale. SPSS and the Process plugin were used to assess mediating and moderating effects.

**Results:** The results indicated that academic self-efficacy was significantly and positively related to college students' learning engagement. The positive predictive impact of academic self-efficacy on learning engagement operated through the fully parallel mediation of psychological resilience and professional commitment. Notably, the mediation effect of professional commitment was greater than that of psychological resilience. Further research found that the mediation of professional commitment was moderated by gender, with female students demonstrating stronger perceptions of professional commitment associated with elevated levels of learning engagement. Gender did not exhibit a significant moderating effect on psychological resilience.

**Conclusions:** College students’ academic self-efficacy, professional commitment, and psychological resilience must be addressed to enhance their learning engagement.

# Introduction

Educators are concerned with the level of learners’ engagement (Zheng, 2023). In the post-pandemic era, effective implementation of measures to enhance learning engagement among college students is a concern for many countries. Research suggests that effective learning hinges on learners themselves (Kumar and Todd, 2022). Effective learning necessitates students’ active participation, the internalization of acquired knowledge, and the formation of their own learning experiences (Rashid and Asghar, 2016). Learning engagement is a crucial factor that influences students’ academic performance (Sahni, 2023). An increasing number of countries are linking the level of learning engagement with academic performance, reward and punishment systems, and dropout and graduation rates.

Learning engagement serves as a crucial predictor of the quality of learning (Bayoumy and Alsayed, 2021). During the pandemic, college students predominantly engaged in home-based learning through the Internet. The learning mode changed significantly from the pre-pandemic period, transitioning from traditional face-to-face group learning to non-contact solo learning. At the conclusion of the pandemic, the students returned to the classroom for face-to-face learning. However, influenced by the learning mode during the pandemic, they demonstrated low learning initiative and diminished levels of learning engagement. Most existing research on learning engagement focuses on its current situation, characteristics, and influencing factors. However, the relationship between academic self-efficacy and learning engagement has been less explored. This study aims to explore the intrinsic mechanism of academic self-efficacy and learning engagement through investigation. It provides new ideas for improving college students' learning engagement in the post-pandemic era..

Learning engagement refers to the amount of time and energy students invest in the learning process and the resulting sustained and abundant affective and cognitive states (Fredricks et al., 2004; Schaufeli et al., 2002). Various theories elucidate the learning engagement process, such as social cognitive and self-determination theories. This study was based on the social cognitive theory, which posits that individual behavior can be influenced by social environmental and personal factors. Self-efficacy is an important concept in social cognitive theory. Bandura believed that self-efficacy is affected by the environment in which it is situated. On the one hand, it affects cognitive processes, with high self-efficacy fostering individual cognitive development, thereby enhancing academic behavior. Onn the other hand, it influences individual behavior (Bandura, 2012). Individuals with high self-efficacy choose challenging academic tasks and invest effort in them. Moreover, when they encounter significant setbacks, they recover swiftly and pursue their goals. Prior research has substantiated the social cognitive theory and identified a close relationship between learning engagement, psychological resilience (Hartley, 2011; Smith et al., 2008; Zeng et al., 2016 ; Zhao et al., 2021), and perceived learning ineffectiveness (Ye, 2023).

Self-efficacy pertains to an individual’s confidence and feelings regarding the organization and the execution of a specific task (Bandura, 1986; Bandura, 1997). First introduced by Bandura, self-efficacy is an important psychological factor that strongly influences behavior and performance. Self-efficacy comprises two components: efficacy and outcome expectations (You, 2022). General self-efficacy is a comprehensive concept. Since its introduction, diverse fields have undertaken extensive research, resulting in the development of derivative concepts, such as academic and organizational self-efficacy.

Academic self-efficacy involves learners’ self-assessment of their learning abilities. Learners exhibit confidence and a sense of competence in organizing and executing specific learning tasks, leading to a successful understanding of learning materials (Bandura, 1997). As a significant predictive factor in learning, it profoundly influences students’ learning behavior and performance, thereby significantly impacting their level of learning engagement. Numerous studies have been conducted to show the positive correlation between college students' academic self-efficacy and learning engagement. Individuals with robust academic self-efficacy showed heightened confidence in completing learning tasks and demonstrated elevated levels of engagement in their studies. Conversely, students with lower academic self-efficacy may experience heightened feelings of helplessness, encounter increased negative emotions, and exhibit reduced participation in their studies (Namaziandost et al., 2023). Academic self-efficacy motivates learners to adopt methods that align with their goals, thereby exerting a substantial influence on the completion of learning tasks. Individuals with robust academic self-efficacy possess a solid cognitive understanding of the learning process and attribute a lack of success to insufficient effort rather than a lack of ability. There is a strong relationship between students' academic self-efficacy and learning engagement (Xie and Xie, 2019). Therefore, we proposed the following hypothesis:

Hypothesis 1: Academic self-efficacy positively predicts learning engagement.

Psychological resilience is an essential ability that enables individuals to enhance their capacity to cope with difficulties and respond effectively to sources of stress when confronted with challenges (Ahern and Norris, 2011; Cooper et al., 2020). Psychological resilience is defined as an individual's ability to maintain a positive adaptive state or “bounce back” to normal life when facing adversity, trauma, misfortune, or significant stressors (Kumpfer, 2002). The psychological resilience framework posits that individuals generate three adaptive outcomes when dealing with stress: an increase in resilience levels, maintaining the original level of resilience, and a decrease in resilience levels after experiencing the shock of stress. The emergence of various adaptive outcomes are influenced by the environment, individual factors, and individual-environment interactions (Luthar et al., 2000).This theory proposes that psychological resilience is dynamic and malleable and plays a crucial protective role in psychological development (Cheung et al., 2019). Psychological resilience is not an inherent personality trait; rather, it continuously develops throughout an individual’s entire life course and is influenced by the surrounding living environment (Gillespie et al., 2007; Celik et al., 2015). Leontopoulou (2006) found that both positive and avoidance coping strategies significantly influenced psychological resilience even in the face of adversity. Individuals with robust psychological resilience exhibit strong adaptive capabilities and a high capacity to absorb and utilize coping strategies. Individuals who experience positive emotions during learning employ various effective strategies to augment their enthusiasm and engagement. Alazemi et al. (2023) discovered that high school students’ academic psychological resilience was positively associated with their self-efficacy.

Social cognitive theory emphasizes that individuals with high self-efficacy possess strong convictions of successfully completing tasks, set challenging goals, and invest energy and perseverance in coping when facing difficulties. In their study of second language learning, Wicaksono et al. (2023) identified a close correlation between self-efficacy, perseverance, academic demotivation, and academic resilience. Self-efficacy and perseverance enable learners to cultivate positive expectations for learning outcomes in the process of second language acquisition, enhance academic resilience, and sustain efficient learning engagement in the long run. Shao and Kang (2022) revealed close relationships among academic psychological resilience, self-efficacy, and learning engagement. Despite encountering challenges, students with academic psychological resilience frequently possess strong confidence in successfully completing learning tasks and believe in their ability to do so. Consequently, they exhibit elevated levels of learning engagement. Rajan et al. (2017) conducted a survey of 155 high school students in India and found that academic resilience differed significantly between male and female students and that there was a significant correlation between academic resilience and self-efficacy among high school students. These studies suggest close relationships among psychological resilience, learning engagement , and academic self-efficacy. However, the underlying mechanisms between these three factors still need to be clarified. Therefore, this study proposed the following hypothesis:

Hypothesis 2: Psychological resilience plays a mediating role between academic self-efficacy and learning engagement..

Professional commitment reflects an individual’s attitude and behavior toward their chosen major, indicating their identification with the major and willingness to invest time and effort in the field of study (Lian et al., 2005), and is a manifestation of individuals' love for and fidelity to their majors. Professional commitment serves as a crucial indicator for comprehending the extent of student engagement in their majors. Previous studies have confirmed that there is a significant correlation between professional commitment and learning engagement. With 750 preschool education college students as participants, Chen (2018) investigated the relationship between learning satisfaction, professional commitment, and learning engagement using a questionnaire method, and found that the participants demonstrated a moderate level of professional commitment while achieving high scores in learning engagement. Learning engagement and professional commitment have a significant positive correlation with each other.

It has been shown that self-efficacy has a strong relationship with professional commitment, particularly emotional commitment. Tsai et al. (2014) suggested that a heightened level of self-efficacy positively influences emotional commitment. This positive effect arises because individuals with elevated self-efficacy are more predisposed to embrace the goals and values of an organization than those with lower self-efficacy. Orgambídez et al. (2019) confirmed the close relationships among job involvement, affective organizational commitment, and self-efficacy. Individuals with high self-efficacy are more emotionally receptive to their workplace and more willing to invest additional energy in their work. Therefore, we proposed the following hypothesis:

Hypothesis 3: Professional commitment mediates academic self-efficacy and learning engagement.

Gender is a crucial demographic variable affecting learning engagement. Male and female students exhibit different preferences in cognitive engagement strategies due to the distinct cognitive structures of their brains. Men possess stronger information processing abilities and more effective metacognitive monitoring and regulatory strategies than women. Women concentrate more on utilizing external learning aids and engaging in cognitive strategy learning than men (Liu, 1997). Furthermore, gender differences exist in the factors that influence learning engagement. Gender differentiation theory suggests that due to the physiological differentiation of gender, individuals gradually develop gender role concepts in the process of social construction. This process implies the ongoing development of individuals and progression of the socialization process. Individuals of different genders uniquely engage in professional learning, adjusting their expectations of their major based on the understanding formed through learning. The level of professional commitment derived from this process is also diverse, resulting in varying levels of learning engagement (Chen, 2018). For instance, men are more suited to majors that cultivate hands-on skills and problem-solving abilities, leading to more proactive and interactive learning behaviors. Conversely, women prefer majors that cultivate reading and critical thinking abilities, resulting in higher levels of learning engagement. Thus, gender differences may exist in how professional commitment affects college students’ learning engagement behaviors. Therefore, we proposed the following hypothesis:

Hypothesis 4: Gender moderates the relationship between professional commitment and learning engagement.

This study integrated social cognitive theory and formulated a moderated parallel mediation model (Figure 1). It explored how college students' academic self-efficacy affects learning engagement behaviors through the parallel mediating roles of psychological resilience and professional commitment, emphasizing the moderating role of gender. This study aimed to offer insights for improving college students’ participation in learning.

# Materials and Methods

## Participants

Undergraduate students, ranging from freshmen to seniors, at a university in Henan, China were recruited to participate in the study using whole cluster random sampling. We employed the anonymous survey platform Wenjuanxing to collect data, garnering a total of 1187 responses. After excluding incomplete or insincere responses, we obtained 1032 valid questionnaires. The participants included 376 freshmen (36.4%), 273 sophomores (26.5%), 263 juniors (25.5%), and 120 seniors (11.6%). There were 479 male participants (46.4%) and 553 female participants (53.6%). The sample consisted of 220 student cadres (21.3%) and 812 non-cadres (78.7%). Moreover, the sample included 148 only children (14.3%) and 884 non-only children (85.7%). Regarding college major selection, 732 participants (70.9%) autonomously chose their majors during the college entrance examination, 107 (10.4%) followed their parents’ and others’ wishes, and 193 (18.7%) adjusted their majors based on arrangements.

The study was conducted from March to September 2023. With the approval of the Academic Committee of Huanghuai University, the study was conducted through the online survey platform Wenjuanxing, with participants collectively tested by class. Before administering the test, the primary examiner described the instructions to the participating students, explained confidentiality, and obtained informed consent from all participants. Participation was anonymous and voluntary, with the option to withdraw, and at the end of the test, a small gift was provided to the participants as a token of appreciation for their consent.

## Psychological Resilience

This study utilized the Chinese version of the Chinese Psychological Resilience Scale (CD-RISC) (Yu, 2007), which was translated and revised by Chinese scholars Yu and Zhang. Developed by the American psychologists Connor and Davidson in 2003, the 25-item CD-RISC comprises three dimensions: self-improvement, toughness, and optimism. Items are measures on a 5-point Likert scale, ranging from 0 (never) to 4 (almost always), for a total score of (0–100). The higher the score, the better the level of psychological resilience. The Cronbach’s α coefficient for CD-RISC was 0.916, and it was 0.963 in this study.

## Academic Self-Efficacy

We used the Academic Self-Efficacy Scale (Liang, 2000), developed by Liang Yusong in 2004. The scale comprises 22 items encompassing two dimensions: self-efficacy for learning ability and self-efficacy for learning behavior. Items are measured on a 5-point scale, ranging from 1 (strongly disagree) to 5 (strongly agree). Questions 14, 16, 17, and 20 were reverse scored, whereas the other items were scored positively. Higher scores on the questionnaire indicate higher academic self-efficacy. Cronbach's alpha coefficient in this study was 0.915.

## Learning Engagement

The learning engagement questionnaire for college students which is used to assess the level of learning engagement [38] consists of 20 questions including three dimensions: behavioral, emotional, cognitive, and engagement. Items are measured on a 5-point scale. The higher the score, the higher the individual's engagement in learning. The Cronbach’s α coefficients for the College Student Learning Engagement Questionnaire and the three sub-scales (behavioral, cognitive, and emotional engagement) were 0.918, 0.825, 0.858, and 0.858, respectively. The Cronbach's α in this study was 0.969.

## Professional Commitment

The College Student Professional Commitment Scale developed by Lian Rong and others was used (Lian et al. 2005). The scale consists of 27 questions and consists of the dimensions of affective commitment, continuance commitment, normative commitment, and ideal commitment. A 5-point Likert scale was used to calculate the scores, ranging from "not at all" to "completely", with scores from 1 to 5 respectively. 6, 8, and 12 are reverse scoring questions. The higher the score, the higher the level of professional commitment. The Cronbach's α was 0.955 in this study.

## Common Method Bias Test

The data collected in this study was solely derived from participants' self-reports, which may introduce common method bias. To assess this potential bias, Harman's one-way test was conducted. The results showed that there were 11 factors with eigenvalues greater than 1. The first factor explained 29.766% of the variance, which was less than the critical criterion of 40% (Zhou & Long, 2004). Therefore, it can be inferred that there is no significant common method bias in this study.

# Results

## Correlation Analysis

As Table 1 shows, academic self-efficacy was positively correlated with psychological resilience, professional commitment, and learning engagement (r=0.340, 0.227, 0.577, respectively; P<0.01). Moreover, psychological resilience was positively correlated with professional commitment and learning engagement (r=0.370, 0.352, respectively; P<0.01), while professional commitment was positively correlated with learning engagement (r=0.320; P<0.01).

## Parallel Mediation Tests

The study tested the mediating effects of psychological resilience, professional commitment between academic self-efficacy and learning engagement using model 4 in the SPSS macro program process developed by Hayes (2015). Results are shown in Table 2 and Figure 2. After controlling for variables such as gender, grade, leadership role, only child status, and hometown, academic self-efficacy positively predicted psychological resilience (β = 0.436, p < 0.001) and professional commitment (β = 0.640, p < 0.001). Both psychological resilience and professional commitment positively predicted learning engagement (β = 0.312, p < 0.001; β = 0.263, p < 0.001). Contrastingly, the predictive effect of academic self-efficacy on learning engagement became insignificant (β=0.001, p=0.976). This suggests that academic self-efficacy does not have a significant direct effect on learning engagement and that professional commitment and psychological resilience fully mediate the relationship between academic self-efficacy and learning engagement.

The results revealed that the mediating effect value for the path academic self-efficacy → psychological resilience → learning engagement was 0.136, and for the path academic self-efficacy → professional commitment → learning engagement was 0.170. The effect values of the 95% confidence intervals did not include 0, indicating that psychological resilience and professional commitment significantly mediated the relationship between college students’ academic self-efficacy and learning engagement. This implies a fully mediating role for psychological resilience and professional commitment in the effect of college students' academic self-efficacy on learning engagement.

To further investigate the reasons for sex disparities in professional commitment, the moderating role of gender between the original parallel mediation models was examined using Model 14. The results are shown in Table 3, where the significance of the original paths is revealed to be consistent with previous observations. Gender exhibited a significant moderating effect on the latter segment of professional commitment mediation (β = -0.217, p < 0.01), whereas the moderating effects on the initial part of professional commitment and both segments of psychological resilience mediation were not statistically significant.

The results were analyzed through simple slope analysis, considering one standard deviation above and below the professional commitment score. Participants were divided into high and low professional commitment groups, to further explore that professional commitment and gender moderated learning engagement. The corresponding plots are shown in Figure 3.

The effect of professional commitment on learning engagement tended to increase for female students, and professional commitment was a significant positive predictor of learning engagement (β = 0.445, t = 8.107, p < 0.001). In contrast, for males, professional commitment remained significant as a predictor of learning engagement. (β= 0.228, t=3.843, p<0.001).

# Discussion

Combining social cognitive theory and the framework of psychological resilience theory, this study explored how academic self-efficacy affects learning engagement. This study revealed the mechanisms by which academic self-efficacy influences learning engagement through psychological resilience and professional commitment, along with gender differences. These findings have theoretical and practical significance for improving students' learning engagement.

Academic self-efficacy and learning engagement were positively correlated, and Hypothesis 1 was supported. Students with high academic self-efficacy are able to deal more effectively with academic difficulties encountered in their educational life. Conversely, those with low academic self-efficacy are prone to harbor self-doubt and resist the execution of learning tasks, thereby avoiding academic failure (Allari, 2020). Maslow’s hierarchy of needs theory posits seven hierarchical needs: physiological, safety, belongingness and love, esteem, cognitive, aesthetic, and self-actualization (Maslow,1987). Maslow argues that satisfaction of lower-level needs is a prerequisite for achieving self-actualization. This theory suggests that students may lack strong learning motivation when certain needs are not met. When students anticipate positive learning outcomes and believe in their ability to complete learning tasks, their need for esteem and cognition becomes exceptionally strong. Once these needs are satisfied, higher-level knowledge-seeking needs emerge, and students continue to choose challenging tasks, willingly investing more resources into the learning process, thus demonstrating higher levels of engagement. Conversely, when students have adverse expectations about learning outcomes and doubt their own capabilities, they may worry about poor grades, leading to potential rejection by teachers and peers. This can result in reluctance to invest excessive energy in learning, potentially leading to learning fatigue and even truancy. In addition, self-doubt regarding one’s learning abilities may gradually lead to learned helplessness and feelings of inferiority. When belongingness, love, and esteem needs are not met, motivation for knowledge seeking tends to weaken.

The influence of academic self-efficacy on learning engagement was observed to be mediated by psychological resilience, confirming Hypothesis 2. Individual factors, such as attention, cognition, emotion, and behavior, can influence the cultivation of psychological resilience. The psychological resilience framework theory suggests that the reasons why individuals experience different adaptation outcomes are determined by a combination of three factors: the environment, intra-individual factors, and individual-environment interactions. The personal factors contributing to psychological resilience comprise cognitive, emotional, physical, mental, and behavioral aspects. Positive emotions can broaden an individual’s attention and cognition, as well as continuously build personal positive resources, enhancing behavioral positivity (Chmitorz et al., 2018). The present study supports this theory by demonstrating that individuals with a high level of psychological resilience have a more positive mood, an optimistic attitude, a greater belief in their abilities, and engage in more positive actions when faced with learning tasks. This proactive behavior, in turn, motivates individuals to invest more effort in the learning process. Psychological resilience originates from a specific belief system that encompasses one’s views of oneself, others, and the goodness and beauty of the world. This belief system is influenced by various factors associated with an individual’s life stages (Jew et al., 1999).

Hypothesis 3 was also supported by the results of the study, showing that professional commitment plays a mediating role between academic self-efficacy and learning engagement, which is consistent with previous studies' findings. In a study conducted by Lu et al. (2023), which involved a professional commitment survey of over 400 medical students, it was found that self-efficacy affects academic performance through learning engagement and professional commitment. In other words, students who assess their learning abilities positively often express strong affection for their chosen profession. They have high expectations for development in their chosen field, willingly adhere to the norms and requirements of their chosen profession, believe in their ability to overcome internal and external challenges in learning, continuously experience and validate their ideas in practical learning, and invest energy into professional learning.

This study found that the role of professional commitment on learning engagement was moderated by gender, thereby confirming and Hypothesis 4. This may be closely related to traditional gender role positioning or societal expectations. During the process of socialization, individuals acquire gender-cognitive schemas, which lead to the manifestation of distinct gender tendencies (Skaar et al., 2014). Women tend to display emotional and compliant traits. They emotionally endorse their chosen majors, unconsciously idealize their academic pursuits, and willingly invest more energy in their studies. In contrast, men tend to exhibit rational traits. They seek novelty and diversity in their thoughts, exhibit a strong sense of control, and provide comprehensive and objective evaluations of their chosen majors. Students are easily influenced by their ingrained cognitive schemas and implicit expectations of gender roles, resulting in gender differences in their levels of professional identification.

# Conclusion

In this study, a moderated mediation model was constructed to explore how academic self-efficacy and learning engagement are related. The results found that academic self-efficacy significantly and positively predicted college students' learning engagement and that psychological resilience and professional commitment played parallel mediating roles between academic self-efficacy and learning engagement, with the mediating role of professional commitment being greater than that of psychological resilience. The predictive role of academic self-efficacy on college students' learning engagement was fully mediated by psychological resilience and professional commitment. Furthermore, the study revealed a gender moderation in the latter part of the pathway for professional commitment. Specifically, women exhibited stronger professional commitment than men, leading to elevated levels of learning engagement.

# Implications and Limitations

## Implications

This study has both theoretical and practical significance, as it explored methods for enhancing learning engagement in the post-pandemic era. First, this study attempts to construct a mediation model wherein academic self-efficacy impacts learning engagement through psychological resilience and professional commitment. This model complements the existing understanding of the influences of self-efficacy on learning engagement within social cognitive theory. It expands the pathway through which academic self-efficacy impacts learning engagement, providing a theoretical basis for further in-depth understanding of the mechanism of academic self-efficacy impacting learning engagement. Second, this study has important practical implications for improving college students' engagement in learning. In the post-pandemic era, blended learning has emerged as a trend, and learning engagement stands out as a key factor influencing the quality of online learning. Therefore, effectively enhancing students’ learning engagement has become particularly important. Based on the results of this study, interventions can be developed to improve both the psychological resilience and the level of professional commitment of college students. Considering the positive effects of professional commitment, one approach is to encourage students to consider their individual characteristics and career preferences while choosing a college major. Students should thoroughly understand the study content, future employment directions, and prospects of the chosen major to enhance their emotional satisfaction with their field of study. Alternatively, students who cannot adapt to their chosen majors after a certain period during the first year should be allowed to make adjustments. School departments could support students by conducting career aptitude tests to help them choose a more suitable major. Leveraging the positive impact of psychological resilience, teachers could integrate positive psychology content, such as resilience education, into classrooms and daily activities to enhance college students’ learning engagement and increased their psychological resilience. Especially for students facing psychological trauma and learning challenges due to COVID-19, focused interventions, such as psychological counseling, group counseling, and therapy, are required to facilitate their swift recovery to the initial level of psychological resilience.

## Limitations

There are a number of shortcomings in this study. First, the reliance on self-reported data introduces inherent reporting biases that are challenging to eliminate. Second, the cross-sectional design of this study does not allow for an in-depth consideration of causal relationships between variables. Future research could benefit from experimental designs and longitudinal studies to further establish causal relationships between variables. Third, this study only examined the role of professional commitment and psychological resilience in the relationship between academic self-efficacy and learning engagement. Subsequent studies should explore additional variables with potential mediating or moderating effects, including parenting style, peer support, and future orientation.

# Conflict of Interest

The authors declare that the research was conducted in the absence of any commercial or financial relationships that could be construed as a potential conflict of interest.

# Author Contributions

YXW conceived the study, drafted the manuscript, and took responsibility for the manuscript as a whole. YXW provided advice on study design and supervised the data collection. WZ participated in data collection and data analysis. All authors contributed to the article and approved the submitted version.

# Funding

Not applicable.

# Acknowledgments

# We want to thank all the participants in this study for their cooperation.

# References

Ahern, N.R., and Norris, A.E. (2011). Examining factors that increase and decrease stress in adolescent community college students. J. Pediatr. Nurs. 26, 530–540.https://doi.org/530-540.10.1016/j.pedn.2010.07.011

Alazemi, A.F.T., Jember, B., and Al-Rashidi, A.H. (2023). How to decrease test anxiety: a focus on Academic Emotion Regulation, L2 grit, resilience, and self-assessment. Lang. Test. Asia*.* 13, 1–17. https://doi.org/10.1186/s40468-023-00241-5

Allari, R.S., Atout, M., and Hasan, A.A. (2020). The value of caring behavior and its impact on students’ self‐efficacy: Perceptions of undergraduate nursing students. Nurs. Forum. 55, 259–266. https://doi.org/10.1111/nuf.12424

Bayoumy, H.M.M., and Alsayed, S. (2021). Investigating relationship of perceived learning engagement, motivation, and academic performance among nursing students: a multisite study. Adv. Med. Educ. Pract. 12, 351–369. https://doi.org/10.2147/AMEP.S272745

Bandura, A. (2012). On the functional properties of perceived self-efficacy revisited. J. Manag. 38, 9–44. https://doi.org/10.1177/0149206311410606

Bandura, A. (1986). Social Foundations of Thought and Action: A Social Cognitive Theory. Englewood Cliffs, NJ: Prentice-Hall.

Bandura, A. (1997). Self-Efficacy: The Exercise of Control. New York, NY: Worth Publishers, Incorporated.

Celik, D.A., Cetin, F., and Tutkun, E. (2015). The role of proximal and distal resilience factors and locus of control in understanding hope, self-esteem and academic achievement among Turkish pre-adolescents. Curr. Psychol. 34, 321–345.

Chen, M. (2018). Effect of professional satisfaction on learning engagement in undergraduates major in preschool education: mediating role of professional commitment. Psychol. 9, 2250–2260. https://doi.org/10.4236/psych.2018.98128

Chmitorz, A., Kunzler, A., Helmreich, I., Tüscher, O., Kalisch, R., Kubiak, T., et al. (2018). Intervention studies to foster resilience – A systematic review and proposal for a resilience framework in future intervention studies. Clin. Psychol. Rev. 59, 78–100. https://doi.org/10.1016/j.cpr.2017.11.002

Cheung, V.H.M., Chan, C.Y., and Au, R.K.C. (2019). The influence of resilience and coping strategies on suicidal ideation among Chinese undergraduate freshmen in Hong Kong. Asia-Pac. Psychiatry. 11, 1758–5864. https://doi.org/10.1111/appy.12339

Cooper, A.L., Brown, J.A., Rees, C.S., and Leslie, G.D. (2020). Nurse resilience: A concept analysis. *Int*. *J*. *Ment*. *Health Nurs*. 29, 553–575.https://doi.org/10.1111/inm.12721

Fredricks, J.A., Blumenfeld, P.C., and Paris, A.H. (2004). School engagement: potential of the concept, state of the evidence. Rev. Educ. Res. 74, 59–109. https://doi.org/10.3102/00346543074001059

Gillespie, B.M., Chaboyer, W., and Wallis, M. (2007). Development of a theoretically derived model of resilience through concept analysis. Contemp. Nurse, 25,124–135. https://doi.org/10.5172/conu.2007.25.1-2.124

Hartley, M.T. (2011). Examining the relationships between resilience, mental health, and academic persistence in undergraduate college students. J. Am. College Health. 59, 596–604. https://doi.org/10.1080/07448481.2010.515632

Hayes, A.F. (2015). An index and test of linear moderated mediation. Mult. Behav. Res. 50, 1–22. https://doi.org/10.1080/00273171.2014.962683

Jew, C., Green, K., and Kroger, J. (1999). Development and validation of a neasure of resiliency. Meas. Eval. Couns. Dev. 32, 75–89. https://doi.org/10.1080/07481756.1999.12068973

Kumpfer, K.L. (2002). “Factors and Processes Contributing to Resilience,” in Resilience and Development, Longitudinal Research in the Social and Behavioral Sciences: An Interdisciplinary Series, ed. M.D. Glantz and J.L. Johnson, J.L. (Boston: Kluwer Academic Publishers), 179–224. https://doi.org/10.1007/0-306-47167-1_9

Kumar, S., and Todd, G. (2022). Effectiveness of online learning interventions on student engagement and academic performance amongst first-year students in allied health disciplines: A systematic review of the literature. Focus Health Prof. Educ. 23, 36–55. https://doi.org/10.11157/fohpe.v23i3.430

Luthar, S.S., Cicchetti, D., and Becker, B. (2000). The construct of resilience: a critical evaluation and guidelines for future work. Child Dev. 71, 543–562. https://doi.org/10.1111/1467-8624.00164

Leontopoulou, S. (2006). Resilience of Greek youth at an educational transition point: the role of locus of control and coping strategies as resources. Soc. Indic. Res. 76, 95–126. https://doi.org/10.1007/s11205-005-4858-3

Lu, Y., Tong, K., Wen, M.G., Gong, Y.Y., Zhuang, D. & Zhu, H.Y. (2023). Professional commitment of eight-year medical doctoral degree program students in China: the mediating role of self-efficacy, learning engagement, and academic performance. BMC Med. Educ. 2023. https://doi.org/10.21203/rs.3.rs-3426236/v1

Lian, R., Yang, L.X., and Wu, L.H. (2005). Relationship between professional commitment and learning burnout of undergraduates and scales developing. Acta Psychol. Sin. 37, 632–636.

Liu, R.D. (1997). On the essence of learning strategies. J. Psychol. Sci. 179–181. doi:10.16719/j.cnki.1671-6981.1997.02.024

Liang, S.Y. (2000). Study on achievement goals.Attribution styles and Academic Self- efficacy of Collage Students. [master’s thesis]. [Wuhan]: Central China Normal University.

Maslow, AH. (1987). Motivation and Personality (Third Edition). New York: Harper and Row.

Namaziandost, E., Heydarnejad, T., and Saeedian, S. (2023). Language teacher professional identity: the mediator role of l2 grit, critical thinking, resilience, and self-efficacy beliefs. Iran. J. Appl. Lang. Stud. 14, 107–130. https://doi.org/10.22111/IJALS.2022.7486

Ni, K.X. (2020). Study on the relationship between college students' learning engagement and subjective well-being -- a case study of six universities in chengdu. [master’s thesis]. [Chengdu ]: Chengdu University of Technology. doi:10.26986/d.cnki.gcdlc.2020.001297

Orgambídez, A., Borrego, Y., and Vázquez‐Aguado, O. (2019). Self‐efficacy and organizational commitment among Spanish nurses: the role of work engagement. Int. Nurs. Rev. 66, 381–388. https://doi.org/10.1111/inr.12526

Rajan, S.K., Harifa, P.R., and Pienyu, R. (2017). Academic resilience, locus of control, academic engagement and self-efficacy among the school children. Indian J. Posit. Psychol. 8, 507–511.

Rashid, T., and Asghar, H.M. (2016). Technology use, self-directed learning, student engagement and academic performance: Examining the interrelations. Comput. Hum. Behav. 63, 604–612. https://doi.org/10.1016/j.chb.2016.05.084

Sahni, J. (2023). Is learning analytics the future of online education?: assessing student engagement and academic performance in the online learning environment.
Int. J. Emerg. Technol. Learn. 18, 33–49. https://doi.org/10.3991/ijet.v18i02.32167

Schaufeli, W.B., Martínez, I.M., Pinto, A.M., Salanova, M., & Bakker, A.B. (2002). Burnout and engagement in university students: a cross-national study. J. Cross-Cult. Psychol. 33, 464–481. https://doi.org/10.1177/0022022102033005003

Smith, B.W., Dalen, J., Wiggins, K., Tooley, E., Christopher, P., and Bernard, J. (2008). The brief resilience scale: Assessing the ability to bounce back. Int. J. Behav. Med. 15, 194–200. https://doi.org/10.1080/10705500802222972

Shao,Y., & Kang, S. (2022). The association between peer relationship and learning engagement among adolescents: The chain mediating roles of self-efficacy and academic resilience. *Frontiers in Psychol*. 13, 938756. doi:10.3389/fpsyg.2022.938756

Skaar, N.R., Christ, T.J., and Jacobucci, R. (2014). Measuring adolescent prosocial and health risk behavior in schools: initial development of a screening measure. Sch. Ment. Health. 6, 137–149. https://doi.org/10.1007/s12310-014-9123-y

Tsai, C.W., Tsai, S.H., Chen, Y.Y., & Lee, W.L. (2014). A study of nursing competency, career self-efficacy and professional commitment among nurses in Taiwan. Contemp. Nurse. 49, 96–102. https://doi.org/10.1080/10376178.2014.11081959

Wicaksono, B.H., Ismail, S.M., Sultanova, S.A., and Abeba, D. (2023). I like language assessment: EFL learners’ voices about self-assessment, self-efficacy, grit tendencies, academic resilience, and academic demotivation in online instruction. Lang. Test. Asia. 13, 1–18. https://doi.org/10.1186/s40468-023-00252-2

Xie, D., and Xie, Z. (2019). Effects of undergraduates’ academic self-efficacy on their academic help-seeking behaviors: the mediating effect of professional commitment and the moderating effect of gender. J. Coll. Stud. Dev. 60, 365–371.https://doi.org/10.1353/csd.2019.0035

Ye, J.R., Wu, Y.F, Nong, W., Wu, Y,T., Ye, J.N., and Sun, Y. (2023).The association of short-video problematic use, learning engagement, and perceived learning ineffectiveness among Chinese vocational students. Healthcare. 11, 161. doi:10.3390/healthcare11020161

You, W. (2022). Research on the relationship between learning engagement and learning completion of online learning students. Int. J. Emerg. Technol. Learn. 17, 102–117. https://doi.org/10.3991/ijet.v17i01.28545

Yu, X.N., and Zhang, J.X.A. (2007). Comparison between the Chinese Version of Ego-Resiliency Scale and Connor-Davidson Resilience Scale. J. Psychol. Sci. 169, 1169–1171. doi:10.16719/j.cnki.1671-6981.2007.05.035

Zeng, G., Hou, H., and Peng, K. (2016). Effect of growth mindset on school engagement and psychological well-being of Chinese primary and middle school students: the mediating role of resilience. Front. Psychol. 7, 1664–1078. https://doi.org/10.3389/fpsyg.2016.01873

Zhao, H., Xiong, J., Zhang, Z., & Qi, C. (2021). Growth mindset and college students' learning engagement during the COVID-19 pandemic: A serial mediation model. Front. Psychol., 12, 1664–1078.

https:// doi.org/10.3389/fpsyg.2021.621094

Zheng, C. (2023). Student engagement and academic performance during the COVID-19 pandemic: does a blended learning approach matter?
Int. J. Scholarsh. Teach. Learn. 17, 1–9. https://doi.org/10.20429/ijsotl.2023.17107

Zhou, H., and Long, L.R. (2004). Statistical test and control of common method deviation. Progress in Psychological Science, 12, 942–942.

# Figure Legends

Figure 1. Diagram of the model.

Figure 2. A moderated mediation model

Figure 3. The mediating role of gender in learning engagement and professional commitment

# Tables

Table 1. Descriptive statistics and correlation analysis of variables (n=1032)

| Variable | 1 | 2 | 3 | 4 | 5 | 6 |
| --- | --- | --- | --- | --- | --- | --- |
| 1 Grade  2 Gender  3 AS  4 PR  5 PC  6.LE  M  SD | 1  -0.002  0.101^**^  0.073^*^  0.003  0.056  2.12  1.04 | 1  -0.039  0.015  -0.038  -0.065^*^  1.54  0.50 | 1  0.340^**^  0.227^**^  0.577^**^  3.42  0.50 | 1  0.370^**^  0.352^**^  3.45  0.64 | 1  0.320^**^  3.65  0.68 | 1  3.55  0.55 |

M, mean; SD, standard deviation; AS, academic self-efficacy; PR, psychological resilience; PC, professional commitment; LE, learning engagement. *p < 0.05. **p < 0.01. ***p < 0.001.

Table 2. Results with moderated mediation effects

|  | Influence path | Effect | 95%CI | Relative mediating effect (%) |
| --- | --- | --- | --- | --- |
| Indirect effect | PR | 0.136 | [0.094 0.183] | 44.30% |
|  | PC | 0.170 | [0.104 0.234] | 55.37% |
| Total indirect effect |  | 0.307 | [0.232 0.379] | 99.67% |

CI, confidence interval; PR, psychological resilience; PC, professional commitment.

Table 3. Results with moderated mediation effects

| Regression equation | Fit index | Significance of regression coefficient | | | | |
| --- | --- | --- | --- | --- | --- | --- |
| Outcome variable | Predictor  variable | R | R^2^ | F | β | t |
| Learning engagement |  | 0.341 | 0.117 | 16.877^＊＊＊^ |  |  |
|  | Gender |  |  |  | -0.035 | -0.859 |
|  | birthplace |  |  |  | 0.062 | 1.287 |
|  | Only child or not |  |  |  | 0.107 | 1.739 |
|  | Volunteer choice |  |  |  | 0.000 | 0.003 |
|  | Class post |  |  |  | -0.022 | -0.449 |
|  | Gender × Professional Commitment |  |  |  | -0.217 | -2.999^＊＊^ |

# Data Availability Statement

The datasets [GENERATED/ANALYZED] for this study can be found in the [NAME OF REPOSITORY] [LINK]. Please see the “Availability of data” section of [Materials and data policies in the Author guidelines](https://www.frontiersin.org/guidelines/policies-and-publication-ethics#materials-and-data-policies) for more details.
