# Supplementary material for: The relationship between college students’ learning engagement and academic self-efficacy: a moderated mediation model
Source: Front Psychol. 2024 Sep 3;15:1425172. doi: 10.3389/fpsyg.2024.1425172 (PMC11407112; doi:10.3389/fpsyg.2024.1425172)
Supplement: Supplementary file 1 [file Data_Sheet_1.zip › supplementary materials/manuscript/English article/20240402Article_Text.docx]

The Relationship Between College Students’ Learning Engagement and Academic Self-Efficacy: A Moderated Mediation Model

Yaxing Wang ^1,2^*, Wen Zhang^3^

^1^School of Psychology, Northwest Normal University, Lanzhou, China

^2^Mental Health Service Center, Huanghuai University, Zhumadian, China

^3^Engineering Training Center, Huanghuai University, Zhumadian, China

*** Correspondence:**Yaxing Wang
[wangyxxg@163.c](mailto:email@uni.edu)om

Keywords: learning engagement, academic self-efficacy, professional commitment, psychological resilience.

Abstract

**Introduction:** Despite the return of college students to campus in the post-pandemic era, the deep influence of COVID-19 on learning approaches persists. Existing research has explored fewer mechanisms underlying academic self-efficacy and learning engagement. In line with social cognitive theory, this research investigated academic self-efficacy, professional commitment, psychological resilience, and academic engagement among college students in the post-pandemic era. In this research, the focus was on understanding the impact of academic self-efficacy on learning engagement, taking into account gender as a moderator, and psychological resilience and professional commitment as mediators.

**Methods:** We conducted a survey with 1032 college students in Henan Province, China, utilizing the Psychological Resilience Scale, Academic Self-Efficacy Scale, College Student Learning Engagement Questionnaire, and College Student Professional Commitment Scale. SPSS and the Process plugin were used to assess mediating and moderating effects.

**Results:** Academic self-efficacy significantly and positively correlates with college students' commitment to learning. The positive anticipation of learning engagement facilitated by academic self-efficacy exerts its effect through the fully parallel mediation of psychological resilience and professional commitment. Notably, the mediation effect of professional commitment was greater than that of psychological resilience. Further research found that the mediation of professional commitment was moderated by gender, with female students demonstrating stronger perceptions of professional commitment associated with elevated levels of learning engagement. Gender did not exhibit a significant moderating effect on psychological resilience.

**Conclusions:** College students’ academic self-efficacy, professional commitment, and psychological resilience must be addressed to enhance their learning engagement.

# 1 Introduction

Educators are concerned with the level of learners’ engagement (Zheng, 2023). In the post-pandemic era, effective implementation of measures to enhance learning engagement among college students is a concern for many countries. Research suggests that effective learning hinges on learners themselves (Kumar and Todd, 2022). Effective learning necessitates students’ active participation, the internalization of acquired knowledge, and the formation of their own learning experiences (Rashid and Asghar, 2016). Learning engagement is a crucial factor that influences students’ academic performance (Sahni, 2023). Increasingly, countries are associating the extent of learning engagement with academic performance, reward and punishment systems, as well as dropout and graduation rates.

Learning engagement serves as a crucial predictor of the quality of learning (Bayoumy and Alsayed, 2021). During the pandemic, college students predominantly engaged in home-based learning through the Internet. The learning mode changed significantly from the pre-pandemic period, transitioning from traditional face-to-face group learning to non-contact solo learning. At the conclusion of the pandemic, the students returned to the classroom for face-to-face learning. However, influenced by the learning mode during the pandemic, they demonstrated low learning initiative and diminished levels of learning engagement. Most existing studies on learning engagement have focused on its current situation, characteristics, and influencing factors. However, limited research has been conducted on the association between academic self-efficacy and learning engagement. The purpose of this study is to investigate the internal process that links academic self-efficacy with learning engagement through exploration. It provides new ideas on ways to improve college students' learning engagement level in the post-pandemic era.

Learning engagement refers to the degree of time and effort students invest in the learning process, resulting in sustained and enriched affective and cognitive states(Fredricks et al., 2004; Schaufeli et al., 2002). Various theories elucidate the learning engagement process, such as social cognitive and self-determination theories. Related to this paper is the Social Cognitive Theory, which posits that individual behavior can be influenced by social environmental and personal factors. Self-efficacy is an important concept introduced by social cognitive theory. Bandura believed that self-efficacy is affected by the environment in which it is situated. On the one hand, it affects cognitive processes, with high self-efficacy fostering individual cognitive development, thereby enhancing academic behavior. On the other hand, it influences individual behavior (Bandura, 2012). People with strong self-efficacy select difficult academic projects and work hard to complete them. Moreover, when they encounter significant setbacks, they recover swiftly and pursue their goals. Prior research has substantiated the social cognitive theory and identified a close relationship between learning engagement, psychological resilience (Hartley, 2011; Smith et al., 2008; Zeng et al., 2016; Zhao et al., 2021), and perceived learning ineffectiveness (Ye, 2023).

Self-efficacy pertains to an individual’s confidence and feelings regarding the organization and the execution of a specific task (Bandura, 1986; Bandura, 1997). First introduced by Bandura, self-efficacy is an important predictor variable of learning, which strongly influences behavior and performance. Self-efficacy comprises two components: efficacy and outcome expectations (You, 2022). General self-efficacy is a comprehensive concept. Since its introduction, diverse fields have undertaken extensive research, resulting in the development of derivative concepts, such as academic and organizational self-efficacy.

Academic self-efficacy involves learners’ self-assessment of their learning abilities. Learners exhibit confidence and a sense of competence in organizing and executing specific learning tasks, leading to a successful understanding of learning materials (Bandura, 1997). As a significant predictive factor in learning, it profoundly influences students’ learning behavior and performance, thereby significantly impacting their level of learning engagement. Numerous investigations have shown a positive association between college students' academic self-efficacy and learning engagement. Individuals with robust academic self-efficacy showed heightened confidence in completing learning tasks and demonstrated elevated levels of engagement in their studies. Conversely, students with lower academic self-efficacy may experience heightened feelings of helplessness, encounter increased negative emotions, and exhibit reduced participation in their studies (Namaziandost et al., 2023). Academic self-efficacy motivates learners to adopt methods that align with their goals, thereby exerting a substantial influence on the completion of learning tasks. Individuals with robust academic self-efficacy possess a solid cognitive understanding of the learning process and attribute a lack of success to insufficient effort rather than a lack of ability. Significant associations have been observed between students' academic self-efficacy and their levels of learning engagement (Xie and Xie, 2019). Based on this, we hypothesized the following:

Hypothesis 1: Academic self-efficacy positively predicts learning engagement.

Psychological resilience denotes an individual's capacity to bolster their ability to cope with adversities and respond effectively to sources of stress when confronted with challenges (Ahern and Norris, 2011; Cooper et al., 2020). It refers to the capacity to maintain a positive adaptive state or “bounce back” to normal life when facing adversity, trauma, misfortune, or significant stressors (Kumpfer, 2002). The psychological resilience framework theory posits that individuals generate three adaptive outcomes when dealing with stress: an increase in resilience levels, maintaining the original level of resilience, and a decrease in resilience levels after experiencing the shock of stress. The emergence of various adaptive outcomes are influenced by the environment, individual factors, and individual-environment interactions (Luthar et al., 2000). This theory proposes that psychological resilience is dynamic and malleable, playing a crucial role in safeguarding psychological growth (Cheung et al., 2019). Psychological resilience is not an inherent personality trait; rather, it continuously develops throughout an individual’s entire life course and is influenced by the surrounding living environment (Gillespie et al., 2007; Celik et al., 2015). Leontopoulou (2006) found that both positive and avoidance coping strategies significantly influenced psychological resilience even in adversity. Individuals with robust psychological resilience exhibit strong adaptive capabilities and a high capacity to absorb and utilize coping strategies. Individuals who experience positive emotions during learning employ various effective strategies to augment their enthusiasm and engagement. Alazemi et al. (2023) discovered that high school students’ academic psychological resilience was positively associated with their self-efficacy.

Social cognitive theory highlights that self-efficacious people possess strong convictions of successfully completing tasks, set challenging goals, and invest energy and perseverance in coping when facing difficulties. In their study of second language learning, Wicaksono et al. (2023) discovered a robust association between self-efficacy, perseverance, academic demotivation, and academic resilience. Self-efficacy and perseverance enable learners to cultivate positive expectations for learning outcomes in the process of second language acquisition, enhance academic resilience, and sustain efficient learning engagement in the long run. Shao and Kang (2022) revealed close relationships among academic psychological resilience, self-efficacy, and learning engagement. Despite encountering challenges, students with academic psychological resilience frequently possess strong confidence in successfully completing learning tasks and believe in their ability to do so. Consequently, they exhibit elevated levels of learning engagement. Through a survey of 155 high school students in India, Rajan et al. (2017) found a significant gender difference in academic resilience, and academic resilience and self-efficacy were positively correlated among high school students. These studies suggest close relationships among psychological resilience, learning engagement, and academic self-efficacy. However, the underlying mechanisms between these three factors still need to be clarified. Based on this, we hypothesized the following:

Hypothesis 2: Psychological resilience plays a mediating role between academic self-efficacy and learning engagement.

Professional commitment refers to an individual’s attitude and behavior toward their chosen major, indicating their identification with the major and willingness to invest time and effort in the field of study (Lian et al., 2005), and is a manifestation of individuals' love for and fidelity to their majors. Professional commitment serves as a crucial indicator for comprehending the extent of student engagement in their majors. Previous research has substantiated a significant association between professional commitment and learning engagement. With 750 preschool education college students as participants, Chen (2018) investigated their learning satisfaction, professional commitment and learning engagement using a questionnaire method, and found that the participants demonstrated a moderate level of professional commitment while achieving high scores in learning engagement. Learning engagement and professional commitment are significantly and positively correlated.

It has been shown that self-efficacy is closely related to professional commitment, particularly emotional commitment. The study of Tsai et al. (2014) concluded that a heightened level of self-efficacy positively influences emotional commitment. This beneficial impact arises from individuals with higher self-efficacy being more inclined to align with an organization's objectives and principles compared to those with lower self-efficacy. Orgambídez et al. (2019) confirmed the close relationships among job involvement, affective organizational commitment, and self-efficacy. Individuals with higher levels of self-efficacy exhibit greater emotional acceptance of their workplace and demonstrate a greater willingness to invest additional energy toward their professional endeavors. Based on this, we hypothesized the following:

Hypothesis 3: Professional commitment mediates academic self-efficacy and learning engagement.

Gender is a crucial demographic variable affecting learning engagement. Male and female students exhibit different preferences in cognitive engagement strategies due to the distinct cognitive structures of their brains. Men possess stronger information processing abilities and more effective metacognitive monitoring and regulatory strategies than women. Women concentrate more on utilizing external learning aids and engaging in cognitive strategy learning than men (Liu, 1997). Furthermore, gender differences exist in the factors that influence learning engagement. Gender differentiation theory suggests that due to the physiological differentiation of gender, individuals gradually develop gender role concepts in the process of social construction. This process implies the ongoing development of individuals and progression of the socialization process. Individuals of different genders uniquely engage in professional learning, adjusting their expectations of their major based on the understanding formed through learning. The level of professional commitment derived from this process is also diverse, resulting in varying levels of learning engagement (Chen, 2018). For instance, men are more suited to majors that cultivate hands-on skills and problem-solving abilities, leading to more proactive and interactive learning behaviors. Conversely, women prefer majors that cultivate reading and critical thinking abilities, resulting in higher levels of learning engagement. Thus, gender differences may exist in how professional commitment influences the learning engagement of college students. Based on this, we hypothesized the following:

Hypothesis 4: Gender moderates the relationship between professional commitment and learning engagement.

This study integrated social cognitive theory and formulated a moderated parallel mediation model (Figure 1). The study investigated the impact of college students' academic self-efficacy on their learning engagement, examining the mediating roles of psychological resilience and professional commitment, and the moderating effect of gender in the model..This study aimed to offer insights for improving college students’ participation in learning.

# 2 Materials and Methods

## 2.1 Participants

This study was approved by the Research Ethics Committee of the Academic Committee of Huanghuai University.Undergraduate students from freshman to senior year at a university in Henan, China were recruited as participants in the study using whole-cluster random sampling. We employed the anonymous survey platform Wenjuanxing to collect data, garnering a total of 1187 responses. After excluding incomplete or insincere responses, we obtained 1032 valid questionnaires. The participants included 376 freshmen (36.4%), 273 sophomores (26.5%), 263 juniors (25.5%), and 120 seniors (11.6%). There were 479 male participants (46.4%) and 553 female participants (53.6%). The sample consisted of 220 student cadres (21.3%) and 812 non-cadres (78.7%). Moreover, the sample included 148 only children (14.3%) and 884 non-only children (85.7%). Regarding college major selection, 732 participants (70.9%) autonomously chose their majors during the college entrance examination, 107 (10.4%) followed their parents’ and others’ wishes, and 193 (18.7%) adjusted their majors based on arrangements.Of the total, 767 individuals, making up 74.3%, hail from rural regions, while 265, or 25.7%, originate from urban locales.

The study was conducted from March to September 2023. With the endorsement of the Academic Committee of Huanghuai University, the study was conducted through the online survey platform Wenjuanxing, with participants collectively tested by class. Before administering the test, the primary examiner described the instructions to the participating students, explained confidentiality, and obtained informed consent from all participants. Participants were assured that participation was anonymous and voluntary, with withdrawal possible in the middle of the test. A small gift was provided to the participants as a token of appreciation for their consent.

## 2.2 Psychological Resilience

This research utilized the Chinese adaptation of the Connor-Davidson Resilience Scale (CD-RISC), modified by academics Yu and Zhang, to evaluate psychological resilience. Developed by American psychologists Connor and Davidson in 2003, the 25-item CD-RISC comprises three dimensions: self-improvement, toughness, and optimism. Utilizing a 5-point scale, participants rate responses from "1 = never" to "5 = almost always." As scores rise, so does the level of psychological resilience. The Cronbach’s α for CD-RISC was 0.916, and it was 0.963 in this study.

## 2.3 Academic Self-Efficacy

The Academic Self-Efficacy Scale developed by Yusong Liang (2000) was employed to evaluate academic self-efficacy in the current study. The scale comprises 22 items encompassing two dimensions: self-efficacy for learning ability and self-efficacy for learning behavior. A 5-point scale ranging from "1 = strongly disagree" to "5 = strongly agree" was employed. Questions 14, 16, 17, and 20 were reverse scored, whereas the other items were scored positively. Higher scores on the questionnaire indicate stronger academic self-efficacy. The Cronbach's α in this study was 0.915.

## 2.4 Learning Engagement

The learning engagement questionnaire for college students was employed to assess participants level of learning engagement (Ni, 2020). The questionnaire comprises 20 questions including three dimensions: engagement in behavior, cognition, and emotion. A 5-point scale is used, ranging from "1 = not at all compliant" to "5 = fully compliant." Elevated scores signify increased levels of learning engagement. The Cronbach's α in this study was 0.969.

## 2.5 Professional Commitment

The College Student Professional Commitment Scale developed by Lian Rong and others was used to measure professional commitment (Lian et al. 2005). The scale comprises 27 questions organized into dimensions such as affective commitment, continuance commitment, normative commitment, and ideal commitment. A 5-point Likert scale is used to calculate the score, ranging from "1=not at all" to "5=completely". Scores for questions 6, 8, and 12 are reversed-scored. Higher scores indicate a heightened level of professional commitment. The Cronbach's α in this study was 0.955.

## 2.6 Common Method Bias Test

In this research, the gathered data originated from participants' own accounts, potentially leading to common method bias. hence, Harman's one-way test was employed for analysis. The findings revealed 11 factors, each with eigenvalues exceeding 1, accounting for 29.766% of the variance and falling short of the essential threshold of 40% (Zhou and Long, 2004). Consequently, this research does not exhibit a significant common method bias.

# 3 Results

## 3.1 Correlation Analysis

The findings in Table 1 reveal a positive link between academic self-efficacy and factors like psychological resilience,professional commitment and learning engagement(r=0.340, 0.227, 0.577, respectively; P<0.01),There is a positive link between psychological resilience and both professional commitment and learning engagement(r=0.370, 0.352, respectively; P<0.01), as well as between professional commitment and learning engagement(r=0.320; P<0.01).

## 3.2 Parallel Mediation Tests

The present study aimed to investigate the potential mediating roles of psychological resilience and professional commitment in the association between academic self-efficacy and learning engagement. For this analysis, Model 4 of the SPSS macro process, developed by Hayes (2005), was utilized. The findings are displayed in Table 2 and Figure 2. After adjusting for factors like gender, academic grade, leadership position, sole child status, and place of birth, academic self-efficacy positively predicted psychological resilience (β = 0.433, p < 0.001) and professional commitment (β = 0.642, p < 0.001). Both psychological resilience and professional commitment can predict positive learning engagement (β = 0.315, p < 0.001; β = 0.264, p < 0.001). In contrast, academic self-efficacy became insignificant in predicting learning engagement (β= 0.007, p=0.8892). This suggests that academic self-efficacy does not directly influence learning engagement, and that professional commitment and psychological resilience fully mediate the relationship between academic self-efficacy and learning engagement.

Findings from the analysis of mediating effects revealed that the mediating effect value of academic self-efficacy → psychological resilience → learning engagement stood at 0.136, while that of academic self-efficacy → professional commitment → learning engagement was 0.170. The 95% confidence interval for this effect value excluded 0, reflecting the significance of both psychological resilience and professional commitment in the link between academic self-efficacy and learning engagement among college students. Put differently, the link between academic self-efficacy and learning engagement is completely mediated by psychological resilience and professional commitment.

To further explore the reasons for gender differences in professional commitment, Model 14 was used to test gender's moderating role between the original parallel mediator models. The results are shown in Table 3, with the significance of the original paths is revealed to be consistent with previous observations. Gender exhibited a significant moderating effect on the latter segment of professional commitment mediation (β = -0.217, p < 0.01), whereas the moderating effects on the initial part of professional commitment and both segments of psychological resilience mediation were not statistically significant.

To advance our understanding of how professional commitment and gender moderate learning engagement, the results were analyzed by simple slope analysis using one standard deviation above and below the professional commitment scores and dividing professional commitment into high and low groups. The corresponding plots are shown in Figure 3.

Regarding female students, the growing influence of professional commitment on learning engagement showed a notable positive predictive impact (β=0.445, t=8.107, p<0.001). In the case of males, professional commitment continued to be a key determinant of learning engagement (β= 0.228, t=3.843, p<0.001).

# 4 Discussion

Integrating social cognitive theory with the framework of psychological resilience theory, this study investigated the impact of academic self-efficacy on learning engagement. The findings elucidated the pathway through which academic self-efficacy influences learning engagement through psychological resilience and professional commitment, along with gender differences. The findings have theoretical and practical value for improving students' learning engagement.

The existence of a direct link between academic self-efficacy and learning engagement was verified, corroborating Hypothesis 1. Pupils possessing strong self-belief in their academic abilities are better equipped to handle academic challenges. Conversely, students with weak academic self-efficacy tend to experience self-doubt and resist the execution of learning tasks, thereby avoiding academic failure (Allari, 2020). Maslow’s hierarchy of needs theory posits seven hierarchical needs: physiological, safety, belongingness and love, esteem, cognitive, aesthetic, and self-actualization (Maslow, 1987). Maslow argues that satisfaction of lower-level needs is a prerequisite for achieving self-actualization. This theory suggests that students may lack strong learning motivation when certain needs are not met. When students anticipate positive learning outcomes and believe in their ability to complete learning tasks, their need for esteem and cognition becomes exceptionally strong. Once these needs are satisfied, higher-level knowledge-seeking needs emerge, and students continue to choose challenging tasks, willingly investing more resources into the learning process, thus demonstrating higher levels of engagement. Conversely, when students have adverse expectations about learning outcomes and doubt their own capabilities, they may worry about poor grades, leading to potential rejection by teachers and peers. This can result in reluctance to invest excessive energy in learning, potentially leading to learning fatigue and even truancy. In addition, self-doubt regarding one’s learning abilities may gradually lead to learned helplessness and feelings of inferiority. When belongingness, love, and esteem needs are not met, motivation for knowledge seeking tends to weaken.

It was discovered that academic self-efficacy influences learning engagement via psychological resilience, corroborating Hypothesis 2. Individual factors, such as attention, cognition, emotion, and behavior, can influence the cultivation of psychological resilience. The psychological resilience framework theory suggests that the reason why individuals experience different adaptation outcomes are determined by a combination of three factors: the environment, intra-individual factors, and individual-environment interactions. The personal factors contributing to psychological resilience comprise cognitive, emotional, physical, mental, and behavioral aspects. Positive emotions can broaden an individual’s attention and cognition, as well as continuously build personal positive resources, enhancing behavioral positivity (Chmitorz et al., 2018). This study supports this theory and proves that individuals who possess high psychological resilience levels will have a more positive mood, a more optimistic attitude, a greater belief in their abilities, and engage in more positive actions when faced with learning tasks. This proactive behavior, in turn, motivates individuals to invest more effort in the learning process. Psychological resilience originates from a specific belief system that encompasses one’s views of oneself, others, and the goodness and beauty of the world. This belief system is influenced by various factors associated with an individual’s life stages (Jew et al., 1999).

This study's results indicate that professional commitment plays a mediating role between academic self-efficacy and learning engagement, supporting Hypothesis 3, which aligns with the findings of previous studies. Through a professional commitment survey of over 400 medical students, Lu et al. (2023) confirmed the impact of self-efficacy on academic performance was validated by professional commitment and learning engagement. In other words, students who assess their learning abilities positively often express strong affection for their chosen profession. They have high expectations for development in their chosen field, willingly adhere to the norms and requirements of their chosen profession, believe in their ability to overcome internal and external challenges in learning, continuously experience and validate their ideas in practical learning, and invest energy into professional learning.

Ultimately, the findings suggest that gender plays a moderating role in how professional commitment impacts learning engagement, supporting Hypothesis 4. This may be closely related to traditional gender role positioning or societal expectations. During the process of socialization, individuals acquire gender-cognitive schemas, which lead to the manifestation of distinct gender tendencies (Skaar et al., 2014). Women tend to display emotional and compliant traits. They emotionally endorse their chosen majors, unconsciously idealize their academic pursuits, and willingly invest more energy in their studies. In contrast, men tend to exhibit rational traits. They seek novelty and diversity in their thoughts, exhibit a strong sense of control, and provide comprehensive and objective evaluations of their chosen majors. Students are easily influenced by their ingrained cognitive schemas and implicit expectations of gender roles, resulting in gender differences in their levels of professional identification.

# 5 Conclusion

This research developed a moderated mediation model to elucidate the interconnection between academic self-efficacy and learning engagement. As a result, it was discovered that academic self-efficacy significantly forecasts college students' learning engagement, and psychological resilience and professional commitment were discovered to mediate the link between academic self-efficacy and learning engagement in tandem, with the mediation of professional commitment being greater than that of psychological resilience. Academic self-efficacy's role in predicting college students' learning engagement was fully mediated by psychological resilience and professional commitment. Furthermore, the study revealed a gender moderation in the latter part of the pathway for professional commitment. Specifically, women exhibited stronger professional commitment than men, leading to elevated levels of learning engagement.

# 6 Implications and Limitations

## 6.1 Implications

This work has both theoretical and practical implications, as it explored methods for enhancing learning engagement in the post-pandemic era. First, this study attempts to construct a mediation model wherein academic self-efficacy impacts learning engagement via psychological resilience and professional commitment. Expanding the scope of how academic self-efficacy influences learning involvement enhances the understanding of self-efficacy’s role in fostering engagement. Furthermore, this model supplements the insights provided by social cognitive theory, offering a theoretical basis for a deeper comprehension of the interplay between academic self-efficacy and learning engagement. Second, this study is of practical importance for improving college students' learning engagement. In the post-pandemic era, blended online and offline teaching has become a trend, and learning engagement is a key factor affecting online learning quality. Therefore, effectively enhancing students’ learning engagement has become particularly important. Drawing from this study's findings, interventions can be developed to enhance the psychological resilience and professional commitment of college students. Given the beneficial impacts of professional commitment, one approach is to encourage students to consider their individual characteristics and career preferences while choosing a college major. Students should thoroughly understand the study content, future employment directions, and prospects of the chosen major to enhance their emotional satisfaction with their field of study. Alternatively, students who cannot adapt to their chosen majors after a certain period during the first year should be allowed to make adjustments. School departments could support students by conducting career aptitude tests to help them choose a more suitable major. Leveraging the positive impact of psychological resilience, teachers could integrate positive psychology content, such as resilience education, into classrooms and daily activities to enhance college students’ learning engagement and increased their psychological resilience. Especially for students facing psychological trauma and learning challenges due to COVID-19, focused interventions, such as psychological counseling, group counseling, and therapy, are required to facilitate their swift recovery to the initial level of psychological resilience.

## 6.2 Limitations

This research has several limitations. First, the reliance on self-reported data introduces inherent reporting biases that are challenging to eliminate. Second, the employment of a cross-sectional approach in this research precluded a thorough analysis of the causal links among the variables. Future research could benefit from experimental designs and longitudinal studies to further establish causal relationships between variables. Third, the focus of this research was solely on how professional commitment and psychological resilience influence the link between academic self-efficacy and learning engagement. Subsequent studies should explore additional variables with potential mediating or moderating effects, including parenting style, peer support, and future orientation.

# 7 Conflict of Interest

The authors declare that the research was conducted in the absence of any commercial or financial relationships that could be construed as a potential conflict of interest.

# 8 Author Contributions

YXW conceived the study, drafted the manuscript, and took responsibility for the manuscript as a whole. YXW provided advice on study design and supervised the data collection. WZ participated in data collection and data analysis. All authors contributed to the article and approved the submitted version.

# 9 Acknowledgments

We want to thank all the participants in this study for their cooperation.

# 10 References

Ahern, N.R., and Norris, A.E. (2011). Examining factors that increase and decrease stress in adolescent community college students. J. Pediatr. Nurs. 26, 530–540. https://doi.org/530-540.10.1016/j.pedn.2010.07.011

Alazemi, A.F.T., Jember, B., and Al-Rashidi, A.H. (2023). How to decrease test anxiety: a focus on Academic Emotion Regulation, L2 grit, resilience, and self-assessment. Lang. Test. Asia*.* 13, 1–17. https://doi.org/10.1186/s40468-023-00241-5

Allari, R.S., Atout, M., and Hasan, A.A. (2020). The value of caring behavior and its impact on students’ self‐efficacy: Perceptions of undergraduate nursing students. Nurs. Forum. 55, 259–266. https://doi.org/10.1111/nuf.12424

Bayoumy, H.M.M., and Alsayed, S. (2021). Investigating relationship of perceived learning engagement, motivation, and academic performance among nursing students: a multisite study. Adv. Med. Educ. Pract. 12, 351–369. https://doi.org/10.2147/AMEP.S272745

Bandura, A. (2012). On the functional properties of perceived self-efficacy revisited. J. Manag. 38, 9–44. https://doi.org/10.1177/0149206311410606

Bandura, A. (1986). Social Foundations of Thought and Action: A Social Cognitive Theory. Englewood Cliffs, NJ: Prentice-Hall.

Bandura, A. (1997). Self-Efficacy: The Exercise of Control. New York, NY: Worth Publishers, Incorporated.

Celik, D.A., Cetin, F., and Tutkun, E. (2015). The role of proximal and distal resilience factors and locus of control in understanding hope, self-esteem and academic achievement among Turkish pre-adolescents. Curr. Psychol. 34, 321–345.

Chen, M. (2018). Effect of professional satisfaction on learning engagement in undergraduates major in preschool education: mediating role of professional commitment. Psychology 9, 2250–2260. https://doi.org/10.4236/psych.2018.98128

Chmitorz, A., Kunzler, A., Helmreich, I., Tüscher, O., Kalisch, R., Kubiak, T., et al. (2018). Intervention studies to foster resilience – A systematic review and proposal for a resilience framework in future intervention studies. Clin. Psychol. Rev. 59, 78–100. https://doi.org/10.1016/j.cpr.2017.11.002

Cheung, V.H.M., Chan, C.Y., and Au, R.K.C. (2019). The influence of resilience and coping strategies on suicidal ideation among Chinese undergraduate freshmen in Hong Kong. Asia-Pac. Psychiatr. 11, 1758–5864. https://doi.org/10.1111/appy.12339

Cooper, A.L., Brown, J.A., Rees, C.S., and Leslie, G.D. (2020). Nurse resilience: A concept analysis. Int. J. Ment. Health Nurs. 29, 553–575. https://doi.org/10.1111/inm.12721

Fredricks, J.A., Blumenfeld, P.C., and Paris, A.H. (2004). School engagement: potential of the concept, state of the evidence. Rev. Educ. Res. 74, 59–109. https://doi.org/10.3102/00346543074001059

Gillespie, B.M., Chaboyer, W., and Wallis, M. (2007). Development of a theoretically derived model of resilience through concept analysis. Contemp. Nurse 25, 124–135. https://doi.org/10.5172/conu.2007.25.1-2.124

Hartley, M.T. (2011). Examining the relationships between resilience, mental health, and academic persistence in undergraduate college students. J. Am. College Health 59, 596–604. https://doi.org/10.1080/07448481.2010.515632

Hayes, A.F. (2015). An index and test of linear moderated mediation. Mult. Behav. Res. 50, 1–22. https://doi.org/10.1080/00273171.2014.962683

Jew, C., Green, K., and Kroger, J. (1999). Development and validation of a neasure of resiliency. Meas. Eval. Couns. Dev. 32, 75–89. https://doi.org/10.1080/07481756.1999.12068973

Kumpfer, K.L. (2002). “Factors and Processes Contributing to Resilience,” in Resilience and Development, Longitudinal Research in the Social and Behavioral Sciences: An Interdisciplinary Series, ed. M.D. Glantz and J.L. Johnson, J.L. (Boston: Kluwer Academic Publishers), 179–224.

Kumar, S., and Todd, G. (2022). Effectiveness of online learning interventions on student engagement and academic performance amongst first-year students in allied health disciplines: A systematic review of the literature. Focus Health Prof. Educ. 23, 36–55. https://doi.org/10.11157/fohpe.v23i3.430

Luthar, S.S., Cicchetti, D., and Becker, B. (2000). The construct of resilience: a critical evaluation and guidelines for future work. Child Dev. 71, 543–562. https://doi.org/10.1111/1467-8624.00164

Leontopoulou, S. (2006). Resilience of Greek youth at an educational transition point: the role of locus of control and coping strategies as resources. Soc. Indic. Res. 76, 95–126. https://doi.org/10.1007/s11205-005-4858-3

Lu, Y., Tong, K., Wen, M.G., Gong, Y.Y., Zhuang, D., and Zhu, H.Y. (2023). Professional commitment of eight-year medical doctoral degree program students in China: the mediating role of self-efficacy, learning engagement, and academic performance. BMC Med. Educ. 2023. https://doi.org/10.21203/rs.3.rs-3426236/v1

Lian, R., Yang, L.X., and Wu, L.H. (2005). Relationship between professional commitment and learning burnout of undergraduates and scales developing. Acta Psychol. Sin. 37, 632–636.

Liu, R.D. (1997). On the essence of learning strategies. J. Psychol. Sci. 179–181. doi:10.16719/j.cnki.1671-6981.1997.02.024

Liang, S.Y. (2000). Study on achievement goals.Attribution styles and Academic Self- efficacy of Collage Students. [master’s thesis]. [Wuhan]: Central China Normal University.

Maslow, AH. (1987). Motivation and Personality (Third Edition). New York: Harper and Row.

Namaziandost, E., Heydarnejad, T., and Saeedian, S. (2023). Language teacher professional identity: the mediator role of l2 grit, critical thinking, resilience, and self-efficacy beliefs. Iran. J. Appl. Lang. Stud. 14, 107–130. https://doi.org/10.22111/IJALS.2022.7486

Ni, K.X. (2020). Study on the relationship between college students' learning engagement and subjective well-being -- a case study of six universities in chengdu. [master’s thesis]. [Chengdu]: Chengdu University of Technology. doi:10.26986/d.cnki.gcdlc.2020.001297

Orgambídez, A., Borrego, Y., and Vázquez‐Aguado, O. (2019). Self‐efficacy and organizational commitment among Spanish nurses: the role of work engagement. Int. Nurs. Rev. 66, 381–388. https://doi.org/10.1111/inr.12526

Rajan, S.K., Harifa, P.R., and Pienyu, R. (2017). Academic resilience, locus of control, academic engagement and self-efficacy among the school children. Indian J. Posit. Psychol. 8, 507–511.

Rashid, T., and Asghar, H.M. (2016). Technology use, self-directed learning, student engagement and academic performance: Examining the interrelations. Comput. Hum. Behav. 63, 604–612. https://doi.org/10.1016/j.chb.2016.05.084

Sahni, J. (2023). Is learning analytics the future of online education?: assessing student engagement and academic performance in the online learning environment.
Int. J. Emerg. Technol. Learn. 18, 33–49. https://doi.org/10.3991/ijet.v18i02.32167

Schaufeli, W.B., Martínez, I.M., Pinto, A.M., Salanova, M., & Bakker, A.B. (2002). Burnout and engagement in university students: a cross-national study. J. Cross-Cult. Psychol. 33, 464–481. https://doi.org/10.1177/0022022102033005003

Smith, B.W., Dalen, J., Wiggins, K., Tooley, E., Christopher, P., and Bernard, J. (2008). The brief resilience scale: Assessing the ability to bounce back. Int. J. Behav. Med. 15, 194–200. https://doi.org/10.1080/10705500802222972

Shao,Y., and Kang, S. (2022). The association between peer relationship and learning engagement among adolescents: The chain mediating roles of self-efficacy and academic resilience. Front. Psychol. 13, 938756. doi:10.3389/fpsyg.2022.938756

Skaar, N.R., Christ, T.J., and Jacobucci, R. (2014). Measuring adolescent prosocial and health risk behavior in schools: initial development of a screening measure. Sch. Ment. Health. 6, 137–149. https://doi.org/10.1007/s12310-014-9123-y

Tsai, C.W., Tsai, S.H., Chen, Y.Y., and Lee, W.L. (2014). A study of nursing competency, career self-efficacy and professional commitment among nurses in Taiwan. Contemp. Nurs. 49, 96–102. https://doi.org/10.1080/10376178.2014.11081959

Wicaksono, B.H., Ismail, S.M., Sultanova, S.A., and Abeba, D. (2023). I like language assessment: EFL learners’ voices about self-assessment, self-efficacy, grit tendencies, academic resilience, and academic demotivation in online instruction. Lang. Test. Asia. 13, 1–18. https://doi.org/10.1186/s40468-023-00252-2

Xie, D., and Xie, Z. (2019). Effects of undergraduates’ academic self-efficacy on their academic help-seeking behaviors: the mediating effect of professional commitment and the moderating effect of gender. J. Coll. Stud. Dev. 60, 365–371. https://doi.org/10.1353/csd.2019.0035

Ye, J.R., Wu, Y.F., Nong, W., Wu, Y.T., Ye, J.N., and Sun, Y. (2023). The association of short-video problematic use, learning engagement, and perceived learning ineffectiveness among Chinese vocational students. Healthcare 11, 161. doi:10.3390/healthcare11020161

You, W. (2022). Research on the relationship between learning engagement and learning completion of online learning students. Int. J. Emerg. Technol. Learn. 17, 102–117. https://doi.org/10.3991/ijet.v17i01.28545

Yu, X.N., and Zhang, J.X.A. (2007). Comparison between the Chinese Version of Ego-Resiliency Scale and Connor-Davidson Resilience Scale. J. Psychol. Sci. 169, 1169–1171. doi:10.16719/j.cnki.1671-6981.2007.05.035

Zeng, G., Hou, H., and Peng, K. (2016). Effect of growth mindset on school engagement and psychological well-being of Chinese primary and middle school students: the mediating role of resilience. Front. Psychol. 7, 1664–1078. https://doi.org/10.3389/fpsyg.2016.01873

Zhao, H., Xiong, J., Zhang, Z., and Qi, C. (2021). Growth mindset and college students' learning engagement during the COVID-19 pandemic: A serial mediation model. Front. Psychol. 12, 1664–1078. https:// doi.org/10.3389/fpsyg.2021.621094

Zheng, C. (2023). Student engagement and academic performance during the COVID-19 pandemic: does a blended learning approach matter? Int. J. Scholarsh. Teach. Learn. 17, 1–9. https://doi.org/10.20429/ijsotl.2023.17107

Zhou, H., and Long, L.R. (2004). Statistical test and control of common method deviation. Prog. Psychol. Sci. 12, 942–942.

# 11 Data availability statement

The raw data supporting the conclusions of this article will be made available by the authors, without undue reservation.

# 12 Figure Legends

Figure 1. Diagram of the model.

Figure 2. A moderated mediation model.

Figure 3. The moderating role of gender in learning engagement and professional commitment.

# 13 Tables

Table 1. Descriptive statistics and variable correlation analysis(n=1032)

| Variable | 1 | 2 | 3 | 4 | 5 | 6 |
| --- | --- | --- | --- | --- | --- | --- |
| 1 Grade  2 Gender  3 AS  4 PR  5 PC  6.LE  M  SD | 1  -0.002  0.101^**^  0.073^*^  0.003  0.056  2.12  1.04 | 1  -0.039  0.015  -0.038  -0.065^*^  1.54  0.50 | 1  0.340^**^  0.227^**^  0.577^**^  3.42  0.50 | 1  0.370^**^  0.352^**^  3.45  0.64 | 1  0.320^**^  3.65  0.68 | 1  3.55  0.55 |

M, mean; SD, standard deviation; AS, academic self-efficacy; PR, psychological resilience; PC, professional commitment; LE, learning engagement. *p < 0.05. **p < 0.01. ***p < 0.001.

Table 2. Results with moderated mediation effects

|  | Influence path | Effect | 95%CI | Relative mediating effect (%) |
| --- | --- | --- | --- | --- |
| Indirect effect | PR | 0.136 | [0.094 0.182] | 44.30% |
|  | PC | 0.170 | [0.104 0.234] | 55.37% |
| Total indirect effect |  | 0.307 | [0.232 0.379] | 99.67% |

CI, confidence interval; PR, psychological resilience; PC, professional commitment.

Table 3. Results with moderated mediation effects

| Regression equation | Fit index | Significance of regression coefficient | | | | |
| --- | --- | --- | --- | --- | --- | --- |
| Outcome variable | Predictor  variable | R | R^2^ | F | β | t |
| Learning engagement |  | 0.341 | 0.117 | 16.877^＊＊＊^ |  |  |
|  | Gender |  |  |  | -0.035 | -0.859 |
|  | birthplace |  |  |  | 0.063 | 1.287 |
|  | Only child or not |  |  |  | 0.107 | 1.739 |
|  | Volunteer choice |  |  |  | 0.000 | 0.003 |
|  | Class post |  |  |  | -0.022 | -0.449 |
|  | Gender × Professional Commitment |  |  |  | -0.217 | -3.000^＊＊^ |
