# Supplementary figures and images for: The relationship between college students’ learning engagement and academic self-efficacy: a moderated mediation model
Source: Front Psychol. 2024 Sep 3;15:1425172. doi: 10.3389/fpsyg.2024.1425172 (PMC11407112; doi:10.3389/fpsyg.2024.1425172)

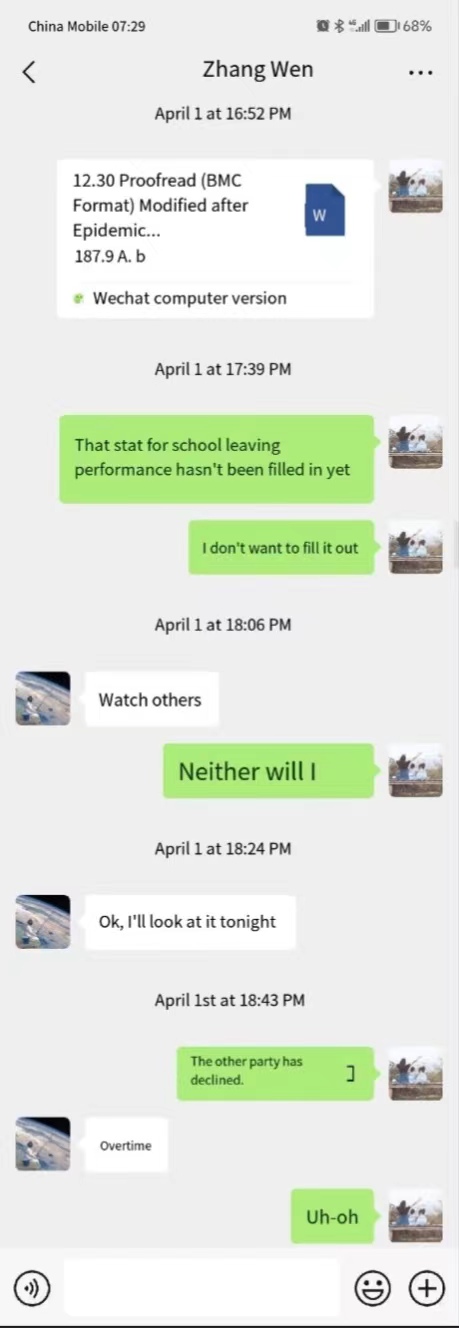

Supplement: Supplementary file 1 [file Data_Sheet_1.zip › supplementary materials/WeChat Chat Records Pictures/April.1.jpg]

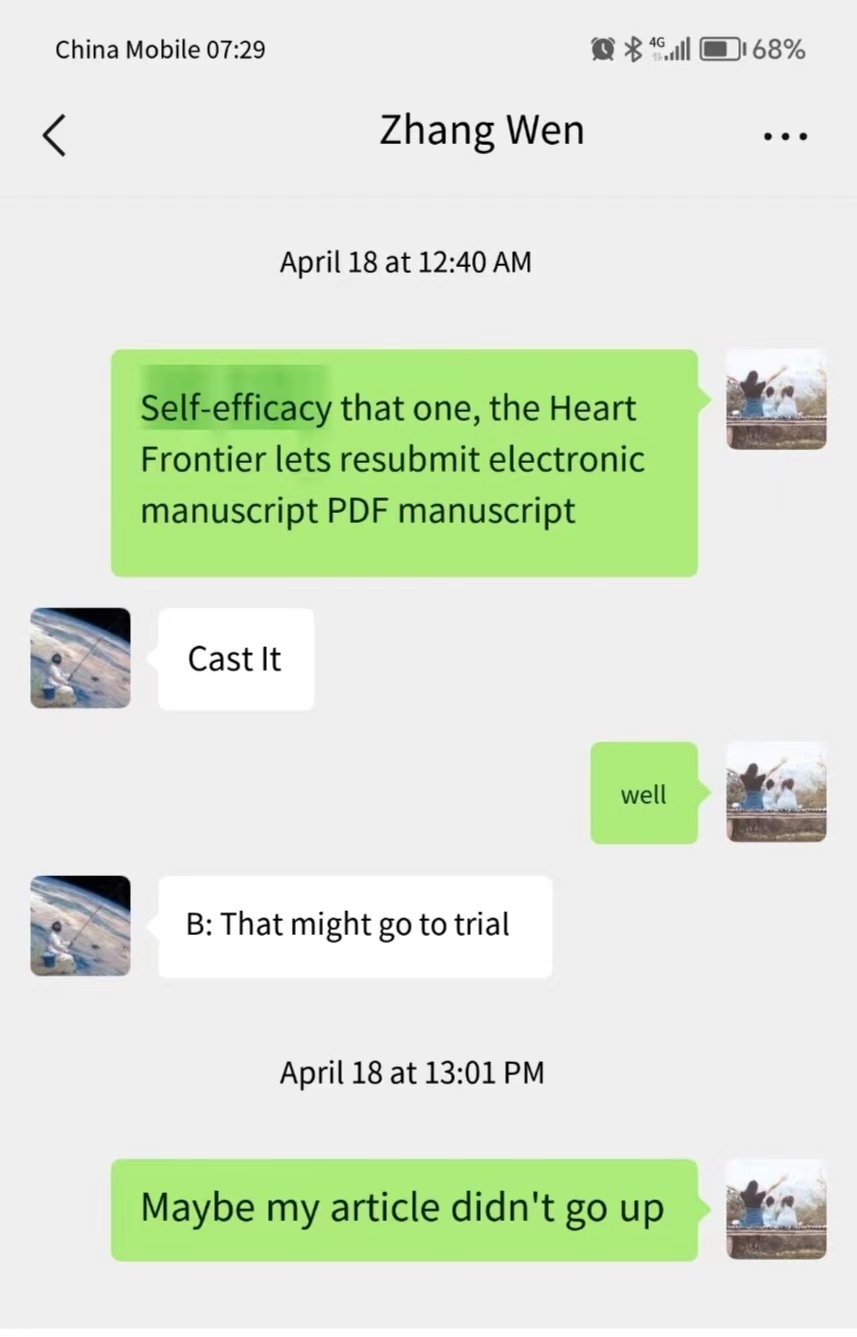

Supplement: Supplementary file 1 [file Data_Sheet_1.zip › supplementary materials/WeChat Chat Records Pictures/April.18.jpg]

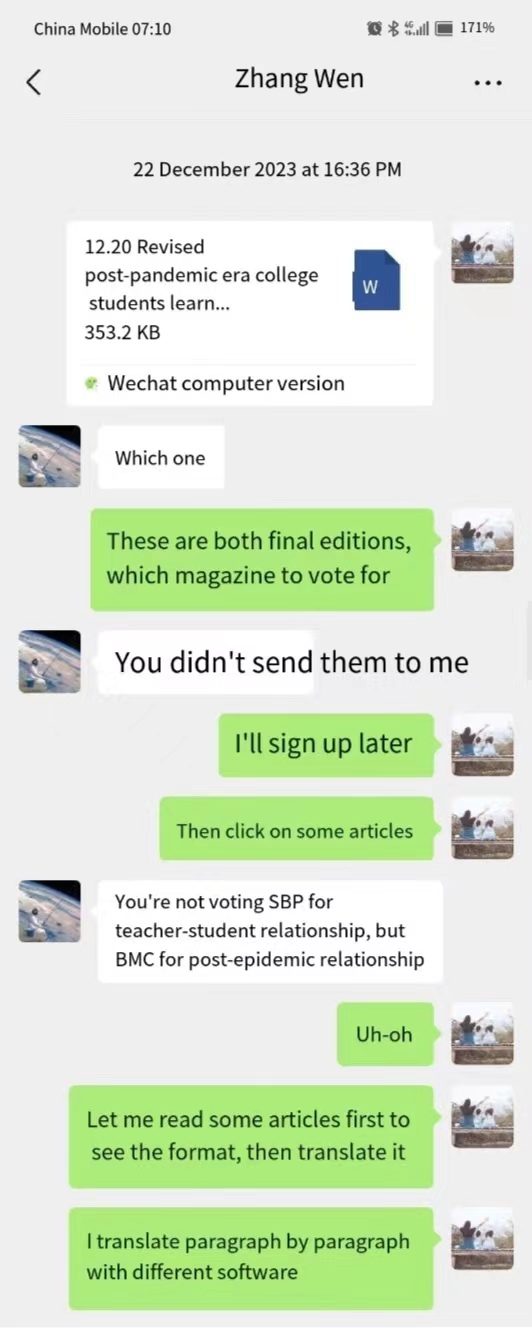

Supplement: Supplementary file 1 [file Data_Sheet_1.zip › supplementary materials/WeChat Chat Records Pictures/Dec.22.jpg]

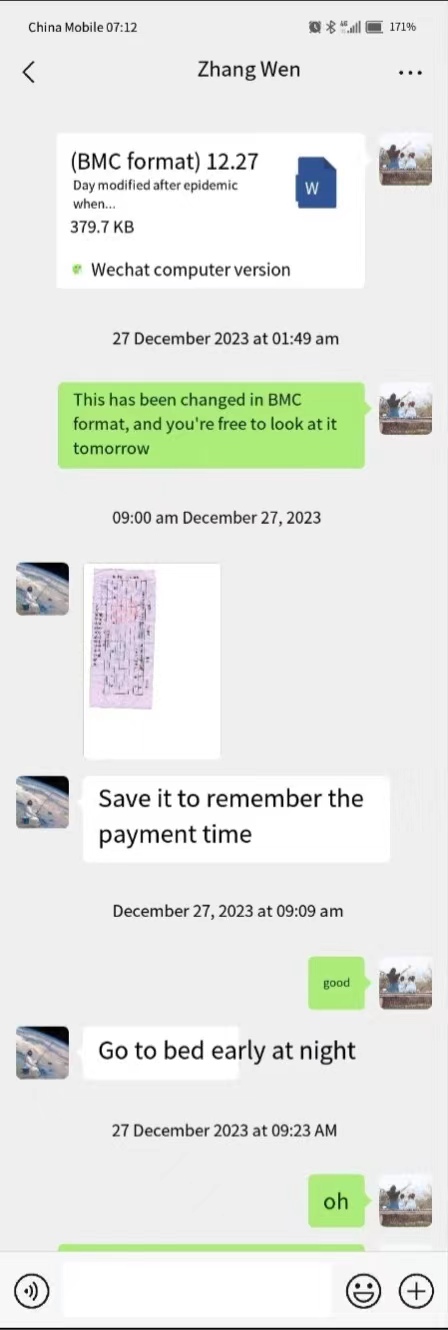

Supplement: Supplementary file 1 [file Data_Sheet_1.zip › supplementary materials/WeChat Chat Records Pictures/Dec.27.jpg]

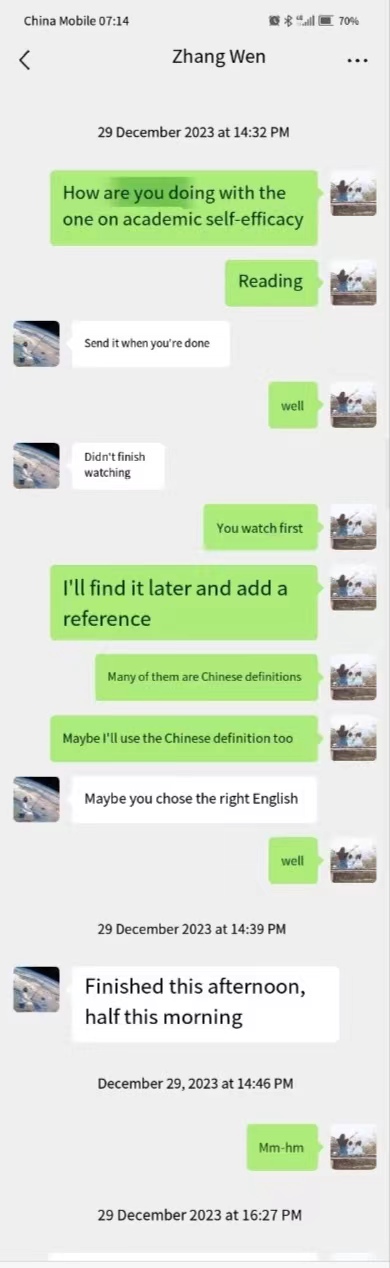

Supplement: Supplementary file 1 [file Data_Sheet_1.zip › supplementary materials/WeChat Chat Records Pictures/Dec.29.jpg]

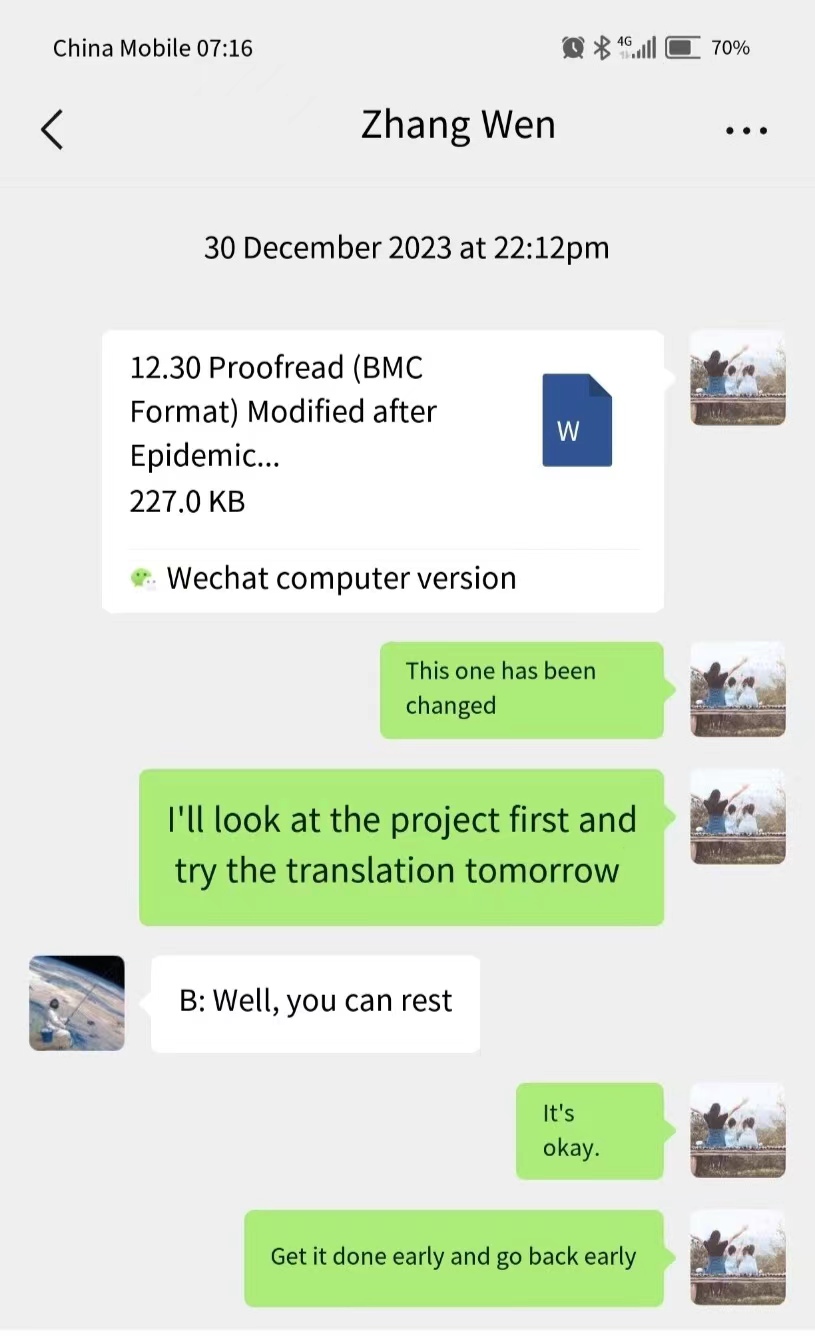

Supplement: Supplementary file 1 [file Data_Sheet_1.zip › supplementary materials/WeChat Chat Records Pictures/Dec.30.jpg]

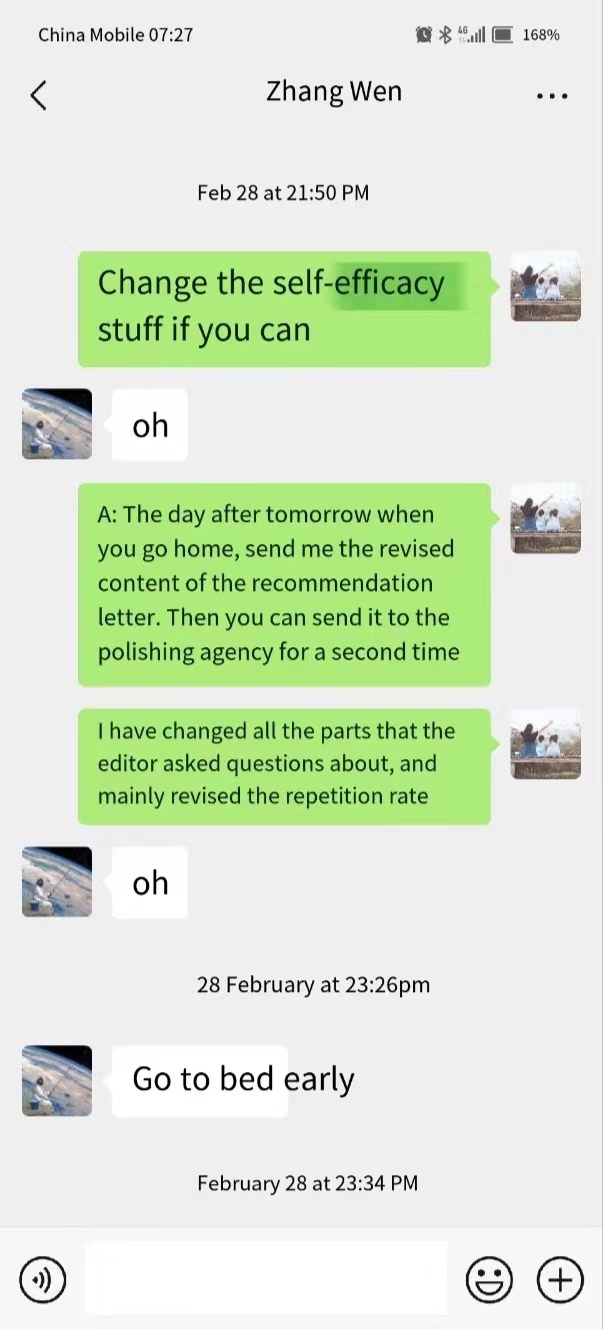

Supplement: Supplementary file 1 [file Data_Sheet_1.zip › supplementary materials/WeChat Chat Records Pictures/Fab.28.jpg]

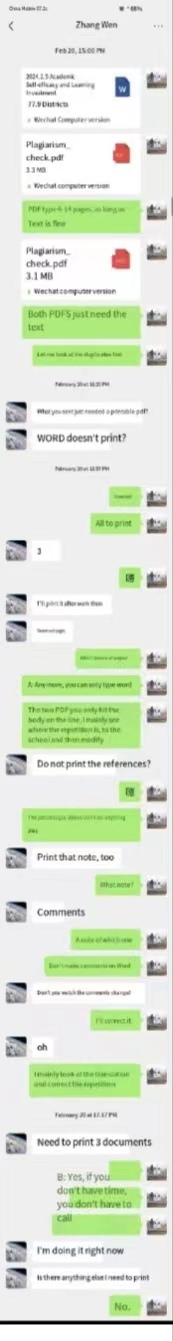

Supplement: Supplementary file 1 [file Data_Sheet_1.zip › supplementary materials/WeChat Chat Records Pictures/Feb.20.jpg]

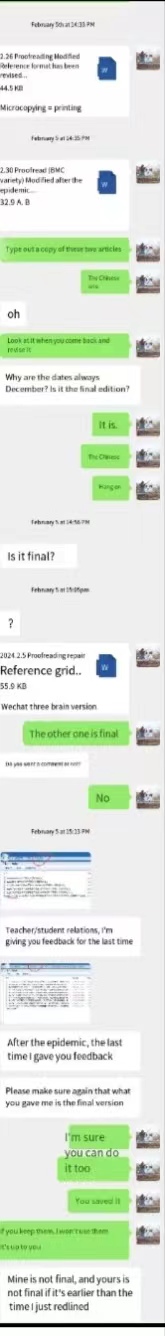

Supplement: Supplementary file 1 [file Data_Sheet_1.zip › supplementary materials/WeChat Chat Records Pictures/Feb.5.jpg]

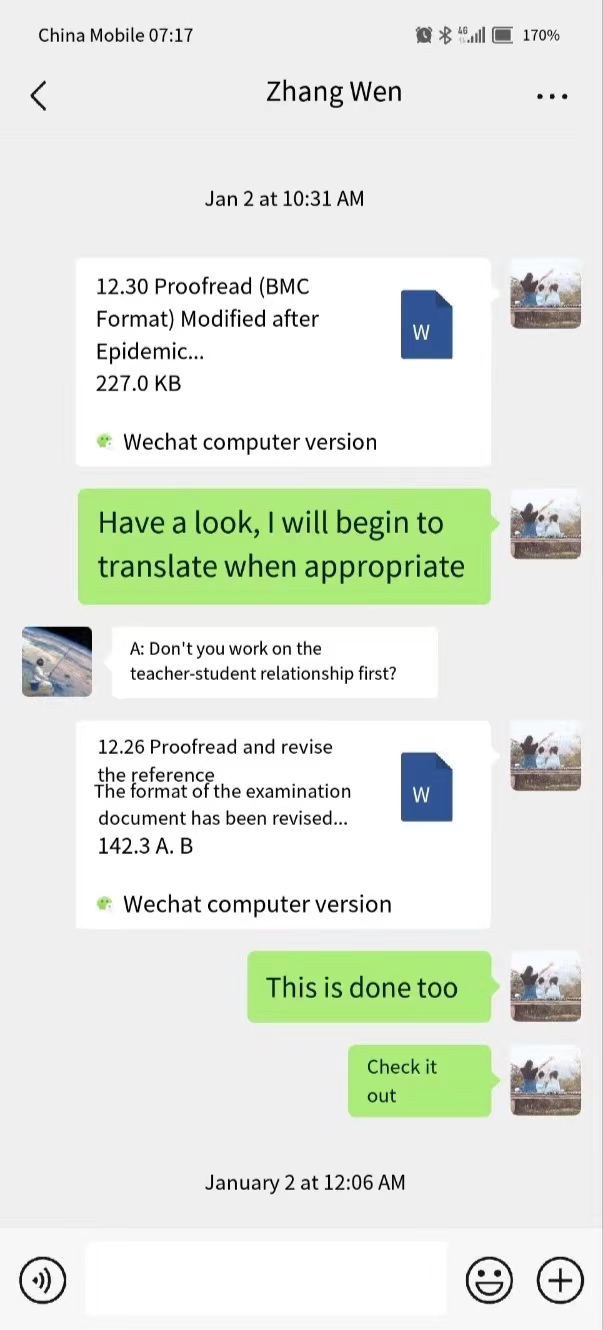

Supplement: Supplementary file 1 [file Data_Sheet_1.zip › supplementary materials/WeChat Chat Records Pictures/Jan.2.jpg]

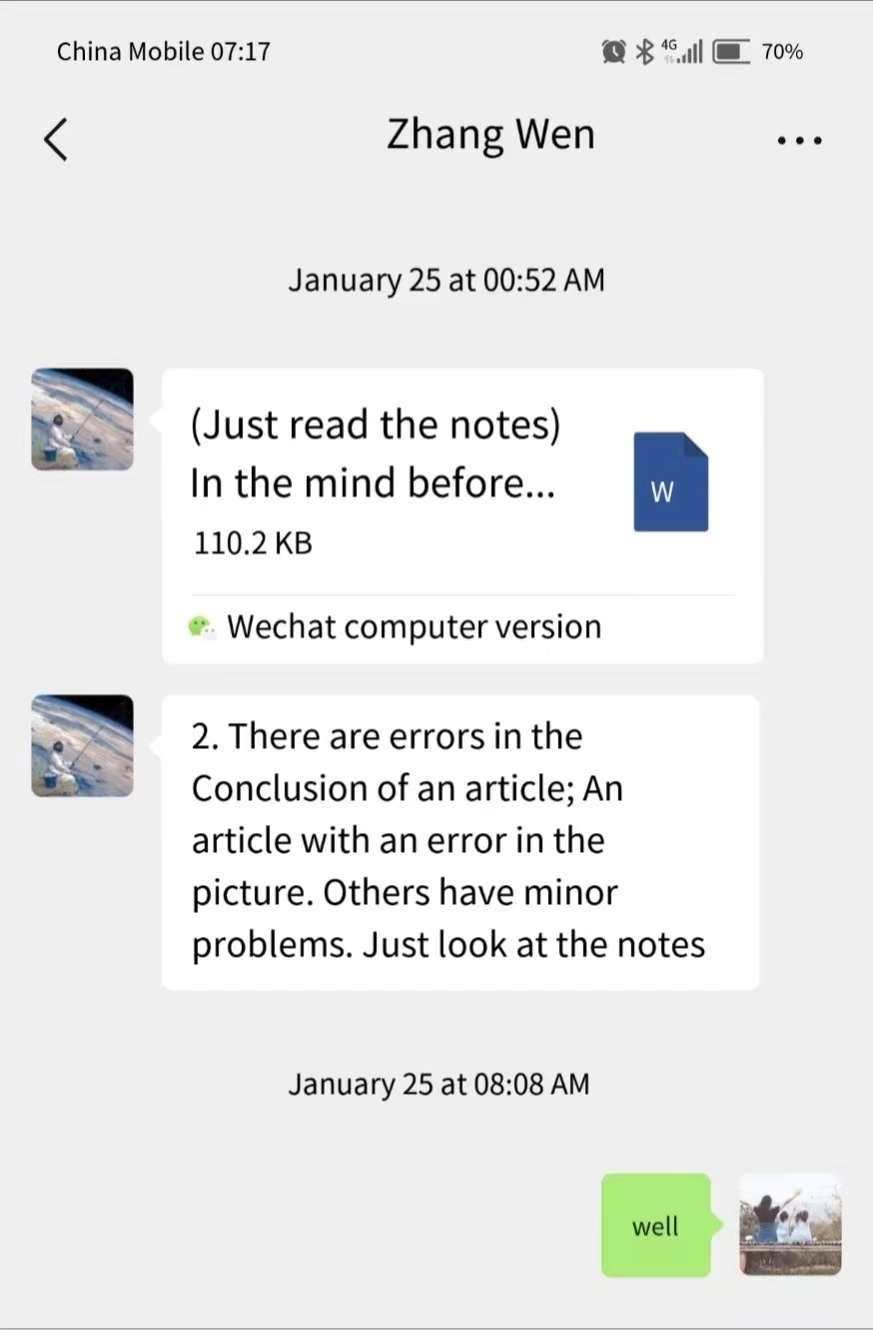

Supplement: Supplementary file 1 [file Data_Sheet_1.zip › supplementary materials/WeChat Chat Records Pictures/Jan.25.jpg]

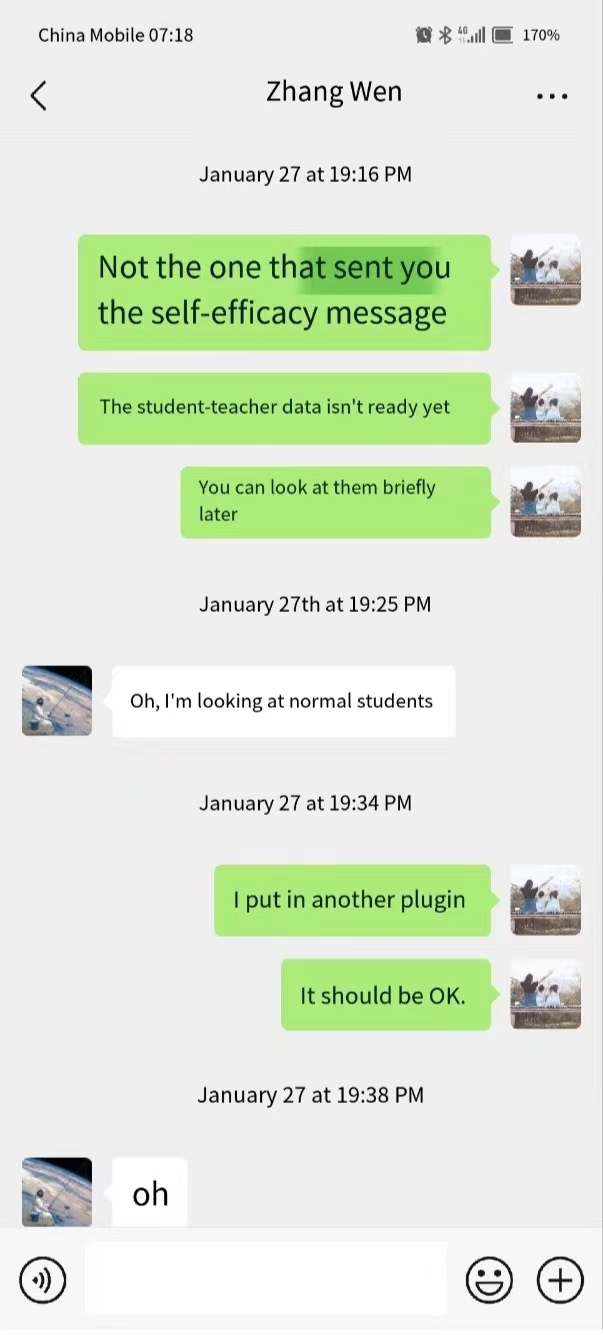

Supplement: Supplementary file 1 [file Data_Sheet_1.zip › supplementary materials/WeChat Chat Records Pictures/Jan.27 (2).jpg]

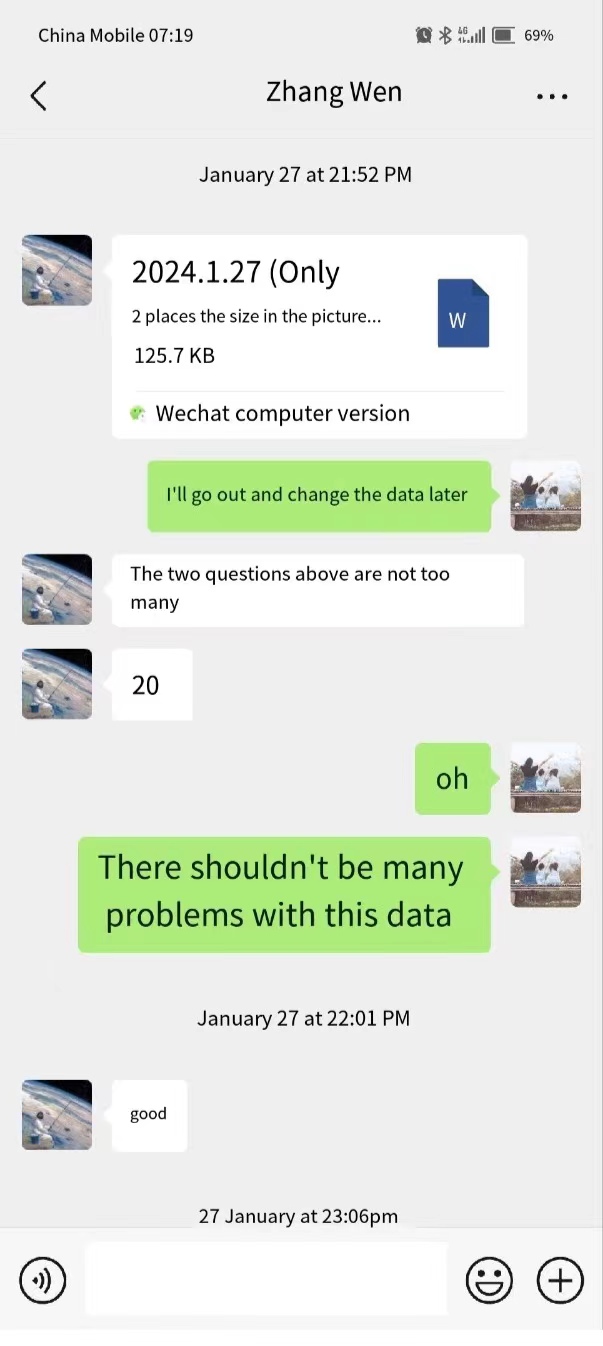

Supplement: Supplementary file 1 [file Data_Sheet_1.zip › supplementary materials/WeChat Chat Records Pictures/Jan.27 (3).jpg]

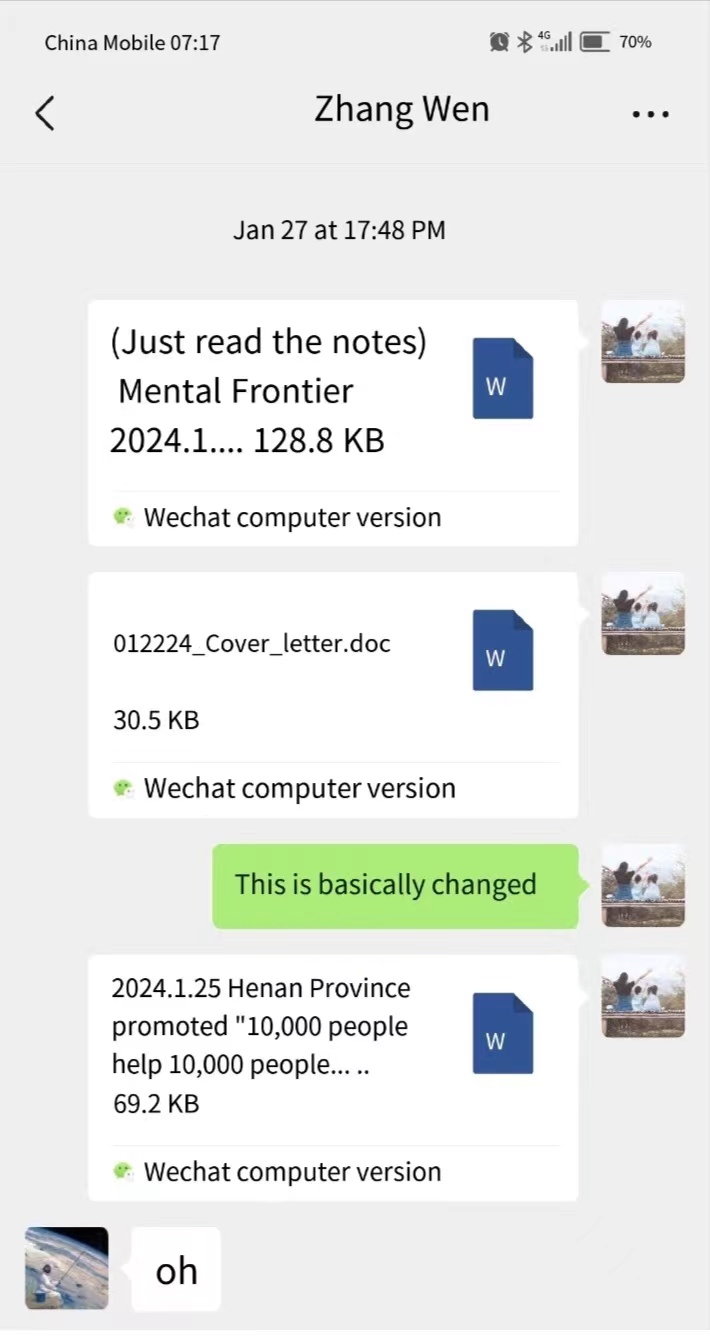

Supplement: Supplementary file 1 [file Data_Sheet_1.zip › supplementary materials/WeChat Chat Records Pictures/Jan.27.jpg]

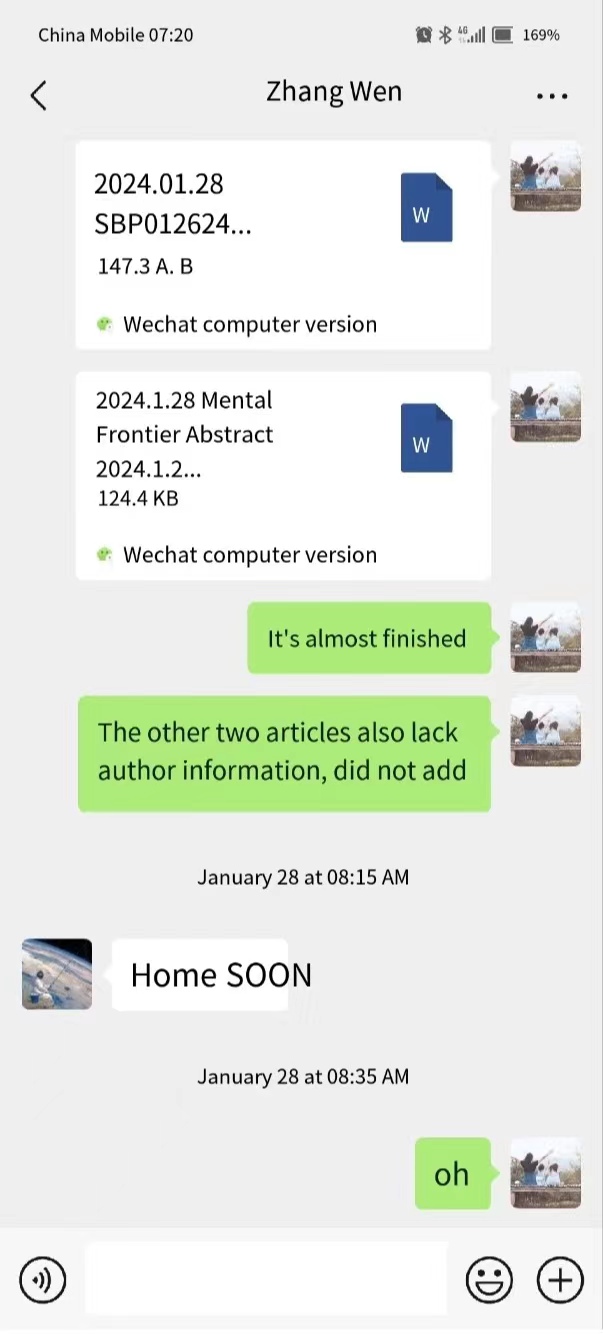

Supplement: Supplementary file 1 [file Data_Sheet_1.zip › supplementary materials/WeChat Chat Records Pictures/Jan.28.jpg]

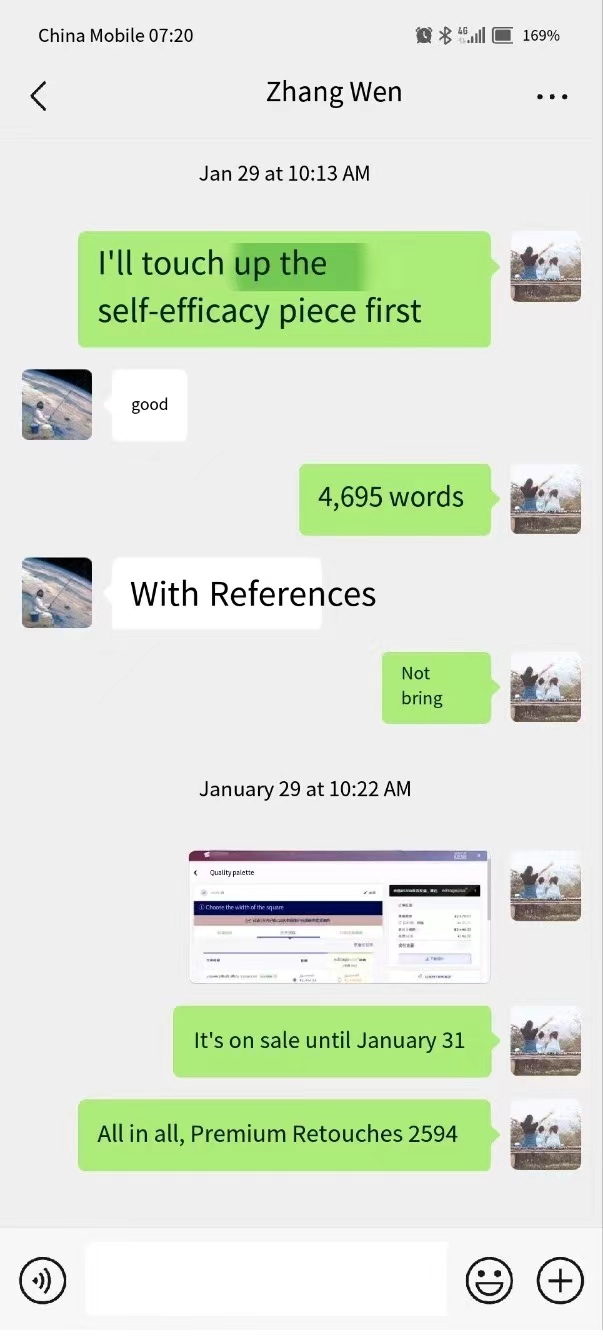

Supplement: Supplementary file 1 [file Data_Sheet_1.zip › supplementary materials/WeChat Chat Records Pictures/Jan.29.jpg]

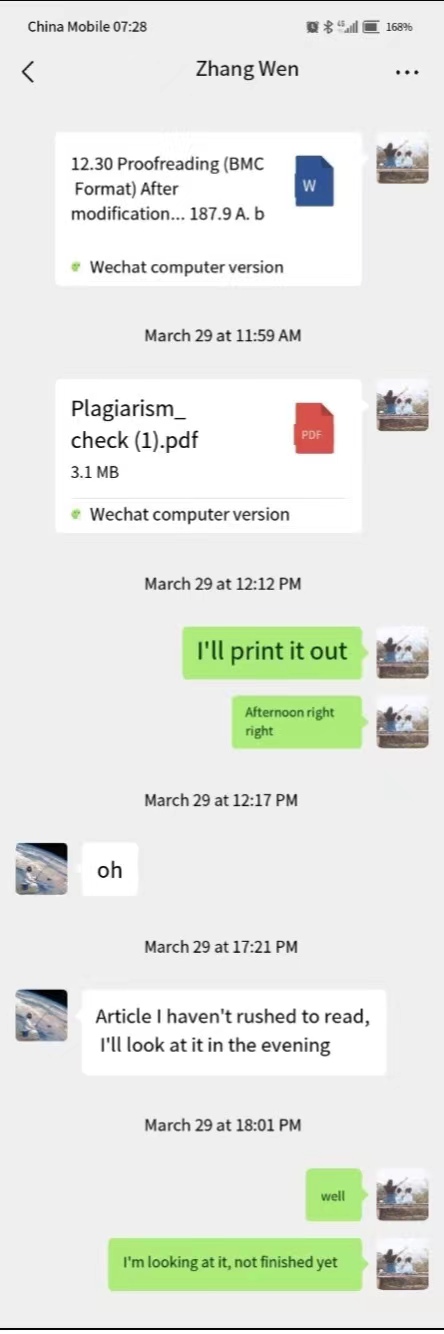

Supplement: Supplementary file 1 [file Data_Sheet_1.zip › supplementary materials/WeChat Chat Records Pictures/Mar.29.jpg]
