# Supplementary material for: The relationship between college students’ learning engagement and academic self-efficacy: a moderated mediation model
Source: Front Psychol. 2024 Sep 3;15:1425172. doi: 10.3389/fpsyg.2024.1425172 (PMC11407112; doi:10.3389/fpsyg.2024.1425172)
Supplement: Supplementary file 1 [file Data_Sheet_1.zip › supplementary materials/manuscript/Chinese article/2023.11.13Chinese article.doc]

后疫情时代大学生学习投入、学业自我效能感、专业承诺与心理弹性的关系研究

摘要：本研究依据社会认知理论，探究了学习投入、学业自我效能感、专业承诺和心理弹性之间的关系。研究以心理弹性、学业自我效能感中介变量、性别为调节变量，探讨学业自我效能感如何影响学习投入。为解决这一问题，我们以河南1032名大学生为被试，使用SPSS的process进行调节中介分析，结果表明学业自我效能感分别通过专业承诺和心理弹性对学习投入起作用，并且专业承诺的中介作用受到情绪的调节，对专业承诺感知水平较高的女生有更高的学习投入水平。性别对心理弹性的调节作用并不显著。

关键词：学习投入，学业自我效能感、专业承诺、心理弹性

**文章重点介绍**

- 学业自我效能感能够显著正向预测大学生学习投入行为。
- 心理弹性和专业承诺在学业自我效能感和学习投入之间起并行中介作用，专业承诺的中介作用大于心理弹性的正向中介作用。
- 性别调节专业承诺路径的后半段路径。具体而言，相对于男生，专业承诺的正向中介作用对女生更强。

引言

学习者的学习投入度是教育者关注的问题，后疫情时代如何开展有效的学习来提高学习投入度，是各个国家关注的问题。越来越多的研究表明有效的学习取决于学习者本人。有效学习的开展需要学生积极参与学习，并把学习到的知识进行内化，形成自己的学习经验。学习投入是影响学生学业成绩的重要因素。越来越多的国家把学习的参与度与学习成绩、奖惩制度，学生辍学率、毕业率联系起来。随着积极心理学的学习投入的效果促使对学习投入的研究激增，

目前关注学习质量。引用数据，说明重要性。学习投入是学习质量的重要预测变量。后疫情时代下大学生学习投入水平低，学习主动性不高，参与度不强，因此有必要进一步探讨在后疫情时代下如何提高大学生学习投入水平。

1.学习投入（Learning engagement）

学习投入是指学生在学习过程中投入的时间、精力及由此引发的持久充沛的情感状态及认知状态(Fredricks et al., 2004; Schaufeli et al., 2002)。有一些理论可以解释学习投入的过程，与本文相关的是社会认知理论和自我决定理论。该理论认为个体行为会受到社会环境因素与个人因素的影响。自我效能感是社会认知理论的核心变量。班杜拉认为自我效能感受到所处环境的影响，一方面会影响认知过程，高的自我效能会促进个体认知发展进而促进学业行为，另一方面会对个体的行为产生影响。以高自我效能为例，高自我效能感的个体会选择富有挑战的学业任务，并为之付出更多的努力，即使遇到重大挫折，也能快速恢复，继续向目标努力。已有研究验证了该理论，学习投入与心理弹性(Hartley, 2011; Smith et al., 2008; Zeng et al., 2016;Zhao et al., 2021)和学业自我效能感关系密切。

学业自我效能感与学习投入的关系

自我效能感指个体对自我能力的评估，是个体对自身组织和执行某一任务的信心和感受。（Bandura, A.，1986；Bandura, A.，1997）。最早由班杜拉提出，是一种重要的心理因素，强烈的影响着人们的行为和表现。自我效能感包括效能预期和结果预期两个组成部分。[1]一般自我效能感是广义的概念，在这一概念被提出后，各个领域开展了广泛的研究，陆续提出了学业效能感，组织效能感等概念。

学业自我效能感即在学习方面的自我效能感，指学生对自己学习能力的评估，是学习者对自己组织和执行某一学习任务，成功理解学习材料的信心和感受（Bandura, A.，1997）。作为学习的一个重要预测变量，它强烈的影响着学生的学习行为和学习表现，在很大程度上会影响学生学习的投入程度。已有众多研究表明大学生学业自我效能感与学习投入之间存在正相关关系。学业自我效能感强的学生，对自己完成学习任务的信心更强，对学业的投入度会更高。相反，学业自我效能感较低的学生，在学习中会体会到更多的无助感，体验到的消极情绪也更多，学习的参与度会降低。自我效能感激励学习者选择适合自己的方式实现目标。学业自我效能感对学生做出决定，完成学习任务有很大的影响。自我效能感强的人对学习过程有良好的认知，对于没有成功的归因多是缺乏努力，而不是能力。Bandura’s (2012)学生的自我效能感及学习投入对学业成功有积极作用。Olivier et al. (2018)，Namaziandost et al. (2022),基于此，提出假设1：学业自我效能感能正向预测学习投入。

3.心理弹性（Psychological resilience）

心理弹性是个体在面临困难时增强情绪容纳力和有效应对压力源的重要能力。（Ahern and Norris.，2011; Cooperet al.，2020）。心理弹性被定义为个体在面临逆境、创伤、不幸和重大压力源时，保持良好的适应状态或从逆境中“反弹”恢复正常生活的能力44。心理弹性框架理论认为人们在应对应激时会产生三种适应结果。第一种是弹性水平更高，第二种是维持原有弹性水平，第三种是受应激惊吓，弹性水平变低。之所以产生不同的适应结果，由所处环境、个体因素以及个人与环境交互作用影响。该理论认为心理弹性具有动态性和可塑性45，是心理发展过程中的一个重要保护因素43。心理弹性不是人格的内在特征，它是在生命的任何时期都可以被发展。(Gillespie al.，2007)文化被认为是影响心理弹性的重要环境因素。(Kitano and Lewis, 2005; Celik al.，2015 )Leontopoulou的研究发现即使在逆境中，积极的应对策略和回避的应对策略都会显著影响心理弹性。(Leontopoulou, 2006)心理弹性良好的个体有很强的适应能力，吸收和利用应对策的能力很强，有研究说明学习弹性能减轻考试焦虑。Alazemi et al.（2023）通过对高中生的调查发现，学业心理弹性和自我效能感之间关系密切。学业心理弹性水平越高，学生的自我效能感越强，反之自我效能感越低。(Rajan et al., 2017)

社会认知理论强调自我效能型的人对自己成功完成某项工作有更强的信念，设定的目标会更具挑战性，在遇到困难时能投入更多的精力和毅力来应对。在第二语言的学习过程中，提高自我效能感和毅力与学术自我效能感和控制消极怠工密切相关。其认为，第二语言学习是语言学习者学习进步的关键，自我效能感和毅力能使学习者在第二语言的学习过程中体验到更多的对学习结果的良好预期，进而长久的保持更加高效的学习投入。(Wicaksono et al., 2023)[2](Wicaksono et al., 2023)一项对不同年龄段小学六年级学生的学业成功、自我效能感和心理弹性进行的纵向随访表明，就开始上小学的年龄阶段而言，被跟踪的学生（Longitudinally Monitored Group）和整个研究组（Whole Group）学业成绩、自我效能感和心理弹性均无显著差异。[4]基于此，提出假设2：心理弹性在学业自我效能感和学习投入中起中介作用。

4.专业承诺（Professional commitment）

专业承诺反映个体对其所学专业的态度和行为，指个体认同所学专业并愿意在学习领域花费时间和精力(Lian et al., 2005)，是个体热爱和忠实于所学专业的表现，也是了解学生专业学习投入度的重要指标。以前的研究已经证实了专业承诺与学习投入之间有显著的相关关系。(Chen, 2018)以750名大学学前专业学生为被试，采用问卷法调查了学前专业大学生学习满意度、专业承诺与学习投入的关系，结果表明学前大学生专业承诺处于中等水平，但是学习投入得分较高。学习投入与专业承诺之间存在显著的正相关关系。

有研究表明自我效能感与专业承诺关系密切，尤其是与情感承诺的关系密切，此外，已有研究表明良好的自我效能水平对情感承诺有积极的影响。这种积极效应的产生是因为自我效能感高的员工比自我效能感低的员工更倾向于接受组织中的目标和价值观(Tsai et al. 2011;2018)。Orgambídez等(2019)的研究验证了工作投入、自我效能感与情感性组织承诺之间关系密切。具有良好自我效能感的个体在情感上更能接受自己的工作单位，更愿意把更多的精力投入到工作中。

专业承诺在学业自我效能感和学习投入之间起中介作用。基于此，提出假设3：专业承诺在学业自我效能感和学习投入中起中介作用。

5. 性别的调节作用

性别是影响学习投入的重要人口学变量，由于男女大脑认知结构的不同导致男女生在认知投入的策略偏好不同，相比女生来说，男生的信息加工能力更强，元认知监控、调节策略更好。女生更关注外在的学习辅助手段及认知策略的{Citation}学习。(刘, 1997)其次，影响学习投入的因素也存在性别差异。性别分化理论指出男女性别的生理分化会在社会建构中形成性别角色观念，也是个体不断成长和社会化的过程，男女生会以自己对专业期待的方式参与专业学习，并根据自己通过学习后形成的对所学专业的理解不断调整对所学专业的期待，由此建构的专业承诺水平也不一样，因此对学习投入的水平也不一样。例如，男生对培养动手操作能力、问题解决能力的专业更加适应，因而其在这类专业中的主动学习行为、互动学习行为更多，女生更加偏爱培养阅读能力、动手能力及思辨能力的专业，其在这类专业中的学习投入度会更高一些。由此推断，专业承诺对大学生学习投入行为的影响可能存在性别差异。的基于此，提出假设4：性别能够调节专业承诺与学习投入间的关系。

综上所述，本研究综合了社会认知理论构建了一个有调节的并行中介模型，探讨大学生的学业自我效能感如何通过心理弹性和专业承诺的并行中介作用影响学习投入行为以及性别在其中的调节作用，以期为提高大学生学习参与度提供参考。

:

图1：模型图

程序

被试

使用整群随机抽样的方式招募来自中国河南一所高校大一至大四本科生参加研究，该研究于2023年3月至9月进行，我们使用问卷星匿名收集材料，共收到问卷1187份，剔除漏题、规律作答等无效问卷后，最终得到有效问卷1032份。参与者包括大一376人，占36.4%；大二273人，占26.5%；大三262人，占25.5%；大四120人，占11.6%。参与者中男生479人，占46.4%；女生553人，占53.6%。学生干部220人，占21.3%；非学生干部812人，占78.7%。自主选择高考专业志愿的732人，占70.9%；遵从父母和他人意愿的107人，占10.4%；调剂安排专业的193人，占18.7%。

**使用问卷**

心理弹性

本研究中采用国内学者 Yu 和 Zhang 翻译并修订的中文版 CD-RISC。该量表由美国心理学家Connor 和 Davidson 于 2003年编制，包括自强、坚韧、乐观等 3个维度，总共25道题目，每个条目采用 Likert 5 级评分法：0 分=“从不”；1 分=“很少”；2分=“有时”；3 分=“经常’；4 分=“几乎总是”，总分为 (0-100)分。得分越高，说明心理弹性水平越好。中文版 CD-RISC 量表 Cronbach's α 系数0.916。本研究中Cronbach's α 系数为0.963.

学业自我效能感

本研究采用梁宇颂（2004）编制的《学业自我效能感量表》，该量表共22道题，包含学习能力自我效能感和学习行为自我效能感2个维度，该量表采用5级评分标准，从“非常不同意”到“非常同意”分别记1-5分。其中14,16,17,20题采用反向计分，其他题目均为正向计分，问卷得分越高说明学生的学业自我效能感越高。本研究中Cronbach's α 系数为0.915.

学习投入

大学生学习投入问卷用来评估学习投入程度(倪, 2020)，问卷共20个题目，包括行为投入、认知投入、情绪投入三个维度。采用五点计分。得分越高，表明被试的学习投入度越高。大学生学习投入问卷及行为投入、认知投入、情绪投入3个分问卷的Cronbach's α系数分别为0.918、0.825、0.858、0.858。该量表的Cronbach's α系数为0.918，表明该量表的信度较好。本研究中Cronbach's α 系数为0.969.

专业承诺

采用连榕等编制的《大学生专业承诺量表》［3］。该量表共 27 道题，并由情感承诺、继续承诺、规范承诺、理想承诺等维度组成。采用李克特 5 点计分法计算得分，从“完全不符合”到“完全符合”分别记1-5分。其中第 6、8、12 为反向计分题。被调查者得分越高，表明其专业承诺水平越高。根据连榕等人的研究，该量表测量的内部一致性 Cronbach α 系数为 0.92。本研究中Cronbach's α 系数为0.955.

共同方法偏差检验

本研究数据均来自于被试自我报告，可能导致共同方法偏差，因此采用 Harman 单因子检验法对其进行检验。结果显示，特征值大于 1的因子共11个，第一个因子解释变异量为 29.766% ，小于40% 的临界标准( 周浩，龙立荣，2004) 。因此，本研究不存在严重的共同方法偏差。

相关分析

**表 1 各变量的描述统计和相关分析（n=1156）**

| 变量 | 1 | 2 | 3 | 4 | 5 | 6 |
| --- | --- | --- | --- | --- | --- | --- |
| 1年级  2性别  3学业自我效能感  4心理弹性  5专业承诺  6.学习投入  平均值  标准差 | 1  -0.002  0.101^**^  0.073^*^  0.003  0.056  2.12  1.04 | 1  -0.039  0.015  -0.038  -0.065^*^  1.54  0.50 | 1  0.340^**^  0.227^**^  0.577^**^  3.42  0.50 | 1  0.370^**^  0.352^**^  3.45  0.64 | 1  0.320^**^  3.65  0.68 | 1  3.55  0.55 |

学业自我效能感与心理弹性、专业承诺、学习投入之间呈正相关，心理弹性与专业承诺、学习投入之间呈正相关，专业承诺与学习投入呈正相关。

并行中介作用检验

研究采用 Hayes( 2018) 编制的 SPSS 宏程序process 中的模型4 对心理弹性、专业承诺在学业自我效能感和学习投入之间的中介效应进行检验。结果如图2所示：在控制了性别、年级、是否班干、是否独生、生源地等变量的条件下，学业自我效能可正向预测心理弹性(β= 0.436，p＜0.001)和专业承诺(β= 0.640，p＜0.001)，心理弹性、专业承诺可预测正向学习投入(β= 0.312，p＜0.001；β= 0.263，p＜0.001)，学业自我效能感不能预测学习投入(β=0.001，p=0.976)。

并行中介模型图

**表2心理弹性和专业承诺在学业自我效能感和学习投入之间的回归分析**

中介效应检验的Bootstrap分析（n=1032）

中介路径	效应值	Boot标准误	95%CI	效应量

直接效应	0.004	[-0.091 0.098]	1.3%

间接效应	心理弹性	0.136	[0.094 0.183]	44.30%

专业承诺	0.170	[0.104 0.234]	55.37%

总效应	0.307	[0.232 0.379]

间接效应对比可知，专业承诺的中介效应值大于心理弹性。

性别的调节作用

回归方程	整体拟合指数 回归系数及显著性

结果变量	预测变量	R	R^2^	F	β	t

学习投入	0.341	0.117	16.877^＊＊＊^

性别	-0.035	-0.859

生源地	0.062	1.287

是否独生	0.107	1.739

志愿选择	0.000	0.003

是否学生干部	-0.022	-0.449

性别×专业承诺	-0.217	-2.999^＊＊^

为进一步探讨专业承诺性别差异的原因，使用模型14检验性别在原并行中介模型间的调节作用。结果如表3所示，原有路径的显著性与先前保持一致，性别对专业承诺中介后半段(β=-0.217，p＜0.01)的调节作用均显著，对专业承诺的前半段调节作用不显著，性别对心理弹性中介前后半段的调节作用均不显著。

为进一步探究专业承诺和性别对学习投入的调节作用，以专业承诺得分的上下一个标准差为原则，将专业承诺分成高专业承诺组和低专业承诺组对结果进行简单斜率分析。简单斜率图见图：

对女生来说，专业承诺对学习投入的影响呈上升趋势，专业承诺对学习投入的正向预测作用显著（β=0.445，t=8.107，p＜0.001) ，而对于男性来说，专业承诺对学习投入的预测作用仍然显著。（β= 0.228，t=3.843，p＜0.001)。

讨论

本研究结合社会认知理论和积极情绪的拓展建构理论，探究了学业自我效能感对学习投入行为的影响，揭示了学业自我效能感通过心理弹性和专业承诺对学习投入的影响机制以及性别差异，对提高学生的学习投入度具有重要的理论和实践价值。

**学业自我效能感与学习投入的关系**

学业自我效能感和学习投入呈正相关。学业自我效能感强的学生能更有效的处理教育生活中遇到的学业困难，而学业自我效能感弱的学生更容易产生自我怀疑，不愿意执行所面临的学习任务，以此避免学业失败。(Allari et al., 2020)马斯洛的需要层次理论提出每个人都有七种层次的需要，从低到高依次为生理需要、安全需要、归属与爱的需要、尊重的需要、认知需要、审美需要、自我实现的需要。马斯洛认为一般低层次需要得到满足，才有可能达到自我实现。该理论认为学生学习动机不强可能是某些层次的需要没有被满足。当学生在学习中对学习结果有很好预期且觉得自己有能力完成学习任务时，尊重的需要和认知的需要会变得异常强烈。这些需要一旦被满足，其会产生更高层次的求知需要，学生会继续选择有挑战性的任务，自愿投入更多的资源到学习过程中，展现出较高的学习参与水平。相反，当学生对学习的结果产生不良的预期，且对自己的能力产生怀疑时，一方面学生会担心成绩差而被老师和同学排斥，不在学习中投入过多精力，进而可能出现学习倦怠，出现逃学行为。另一方面，学生对自己的学习能力产生怀疑，可能会逐渐产生习得性无助，产生自卑心理。一般情况下归属害的需要及自尊的需要得不到满足，求知的需要动力就会变弱。

心理弹性的中介作用

研究发现学业自我效能感通过心理弹性影响学业投入，个体内部的注意、认知、情绪、行为等因素会影响心理弹性的发展。根据积极情绪的拓展建构理论(Fredrickson, 2001)（According to the Broaden-and-Build theory of Positive Emotions），积极情绪能扩展个体注意和认知的范围，还能不断建构个人的积极资源。心理弹性水平高的个体，情绪会更加积极、态度会更加乐观，在面对学习任务时会更加相信自己的能力采取更加积极的行动,这种积极主动的行为会使个体在学习过程中表现出更多的学习投入。学业自我心理弹性源于特定的信念系统，该系统包括一个人对自己、对周围人、对世界真善美的看法。信念系统会受到个人所处人生阶段的各种因素影响。(Jew et al., 1999)在较低水平的逆境环境中，个体一般运用逃避策略来维持自身的心理平衡；在高逆境条件下，个体灵活运用逃避策略和积极策略保持心理健康状态。

专业承诺的中介作用

本研究的结果显示专业承诺在学业自我效能感和学习投入之间起中介作用，这与 的研究结果一致。也就是说那些对自己学习能力有很好评估的学生往往会对自己所学的专业表现出强烈的热爱情感，并对自己所学专业的发展抱有较高的期望，愿意遵守所学专业的规范和要求，相信能以自己的能力去克服学习中所面临的内在和外在挑战，并在实际学习中不断体验和验证自己的想法，对专业学习的投入精力也会越来越多。

性别的调节作用

本研究发现学业自我效能感通过专业承诺影响学习投入的中介作用的后半段受到性别的调节。性别差异检验发现专业承诺可以显著影响男女大学生的学习投入，并且对女生的影响更大。这可能一方面与性别角色的传统定位或社会期望密切相关[5]。本研究中的大学生多为女生，在人们的传统观念中，文科类专业更适合女生就读，女生以后从事文科相关职业更符合社会期望。学生会被人们这种根深蒂固的性别角色认知与期待潜移默化地影响，从而对专业的认同水平更高。是因为另一方面女生更为感性、顺从性较强，在情感上更为认可自己所学专业时，会不自觉的对所学专业产生理想化，把更多的精力投入到学习中去。相比而言，男生会更为理性，控制感较强，对所学专业的评判更为综合和客观。因此，即使拥有相同的专业认同水平，女性的学习投入度更高一些。在专业承诺方面，首先，在填写入学志愿时，学校招生老师可以详细介绍学生所要填写专业的学习内容以及未来的就业方向、就业前景，以此来提高学生对所学专业的情感满意度。其次，在一年级入学一段时间后，允许学生对所学专业进行调整，学校相关部门可以协助学生进行职业能力测试，帮助其选择更为合适的专业在坚持以学生为本的情况下，不断提高本科院校学生的学习投入度，提高本科院校的办学质量。

结论

(1)学业自我效能感能够显著正向预测大学生学习投入行为。

(2)心理弹性和专业承诺在学业自我效能感和学习投入之间起并行中介作用，专业承诺的中介作用大于心理弹性的正向中介作用。

(3)性别调节专业承诺路径的后半段路径。具体而言，相对于男生，专业承诺的正向中介作用对女生更强。

研究价值和不足

研究价值

本研究对变量学习投入进行研究具有重要的理论意义和实践意义。首先，本研究尝试构建学业自我效能感通过心理弹性和专业承诺影响学习投入的中介摸，对社会认知理论中自我效能感对学习投入的相关影响因素进行了补充，扩充了学业自我效能感影响学习投入的作用路径，为进一步深入理解学业自我效能感影响学习投入的作用机制提供了理论基础。本研究对提高大学生学习投入具有重要的实践意义。根据研究的结果，可以从提高大学生心理弹性及专业承诺两方面入手对大学生学习投入进行干预。根据心理弹性正向效应，为提高大学生学习投入水平，应该一方面提倡学生在填写入学志愿时，详细了解所要填写专业的学习内容以及未来的就业方向、就业前景，以此提高对所学专业的情感满意度。另一方面，在一年级入学一段时间后，允许学生对所学专业进行调整，学校相关部门可以协助学生进行职业能力测试，帮助其选择更为合适的专业在坚持以学生为本的情况下，不断提高本科院校学生的学习投入度，提高本科院校的办学质量。根据专业承诺的正向效应，为提高大学生学习投入水平，在课堂及日常活动中增加提高挫折教育及心理弹性等积极心理学内容,以提高学生的心理弹性水平。

**不足**

本研究存在一些不足，首先，研究数据采用自我报告的形式，存在难以消除的共同偏差；其次，本研究收集了大学生被试性别、年级等信息，并未收集年龄等信息，无法考察学习投入行为的年龄差异；最后，本研究采用横断面设计，无法考虑变量间的因果关系，未来可采用实验设计和纵向研究的方法进一步验证变量之间的因果关系。

参考文献

Ahern NR, Norris AE. Examining factors that increase and decrease stress in adolescent community college students. J Pediatr Nurs 2011; 26: 530-540.

Bandura, A. Self-Efficacy: The Exercise ofControl; W.H. Freeman & Co., Ltd.: New York, NY, USA, 1997.

Bandura, A. Social Foundations ofThought and Action: A Social Cognitive Theory; Prentice-Hall: Englewood Cliffs, NJ, USA, 1986.

Bandura, A.; Pastorelli, C.; Barbaranelli, C.; Caprara, G.V. Self-efficacy pathways to childhood depression. J. Pers. Soc. Psychol. 1999, 76, 258–269. [CrossRef] 20. Lent, R.W.; Brown, S.D.; Hackett, G. Toward a unifying social cognitive theory of career and academic interest, choice, and performance. J. Vocat. Behav. 1994, 45, 79–122. [CrossRef]

Cooper AL, Brown JA, Rees CS, et al. Nurse resilience: A concept analysis. Int J Ment Health Nurs 2020; 29: 553-575.

Celik, D. A., Cetin , F., & Tutkun, E. (2015). The role of proximal and distal resilience factors and locus of control in understanding hpe, self-esteem and academic achievement among Turkish pre-adolescents. Current Psychology, 34(2), 321- 345.

Fredricks, J.A., Blumenfeld, P.C., Paris, A.H., 2004. School Engagement: Potential of the Concept, State of the Evidence. Review of Educational Research 74, 59–109. <https://doi.org/10.3102/00346543074001059>

Gillespie, B. M., Chaboyer, W., & Wallis, M. (2007).Development of a theoretically derived model of resilience through concept analysis.ContemporaryNurse,25(1-2),124-135

Lian R, Yang LX, Wu LH (2005) Relationship between professional commitment and learning burnout of undergraduates and scales developing. Acta Psychol Sin 37(05):632–636. https://kns.cnki.net/kcms/detail/detail.aspx?FileName= XLXB200505008&DbName=CJFQ2005

Murat M, Köse S, Savaşer S. Determination of stress, depression and burnout levels of front-line nurses during the COVID-19 pandemic. Int J Mental Health Nurs 2021; 30: 533-543.

Schaufeli, W.B., Martínez, I.M., Pinto, A.M., Salanova, M., Bakker, A.B., 2002. Burnout and Engagement in University Students: A Cross-National Study. Journal of Cross-Cultural Psychology 33, 464–481. <https://doi.org/10.1177/0022022102033005003>

Zhao, H., Xiong, J., Zhang, Z., & Qi, C. (2021). Growth mindset and college Students' learning engagement during the COVID-19 pandemic: A serial mediation model. Frontiers in Psychology, 12, 621094. ht t ps:// doi.org/10.3389/fpsyg.2021.621094

［43］Cheung V，Chan CY,Au R.The influence of resilence and coping strategies on suicidalideation among Chinese undergraduate freshmen in Hong Kong [J].Asia-Pacific Psychiatry.2019,11(2):e12339.

［44］American Psychological Association.Building your resilience[EB/OL].http://www.apa.org/topics/resilience.2020-02-01.

［45］Luthar SS,Backer CB.The construct of resilence:A critical evaluation and guidelines for future work[J].Child Development.2000,71(3):543-562.

[1]Research on the Relationship between Learning Engagement and Learning Completion of Online Learning Students

［3］梁宇颂．大学生成就目标、归因方式与学业自我效能感的研究［D］．武汉：华中师范大学，2000．

[2]I like language assessment: EFL learners’

voices about self‑assessment, self‑efficacy, grit

tendencies, academic resilience, and academic

demotivation in online instruction

[4]Investigation of academic achievement, self-efficacy and psychological resilience

of sixth grade students starting primary school at different ages
