# Supplementary material for: The relationship between college students’ learning engagement and academic self-efficacy: a moderated mediation model
Source: Front Psychol. 2024 Sep 3;15:1425172. doi: 10.3389/fpsyg.2024.1425172 (PMC11407112; doi:10.3389/fpsyg.2024.1425172)
Supplement: Supplementary file 1 [file Data_Sheet_1.zip › supplementary materials/manuscript/Chinese article/2024.1.3Chinese article.doc]

后疫情时代大学生学习投入与学业自我效能感的关系：性别、专业承诺与心理弹性的调节中介模型

摘要

Introduction后疫情时代，虽然大学生已经重返校园，但COVID-19对他们学习方式的深刻影响仍在持续。本研究依据社会认知理论，探究了后疫情时代大学生的学业自我效能感、专业承诺、心理弹性与学习投入之间的关系。研究以心理弹性、专业承诺为中介变量，以性别为调节变量，探讨学业自我效能感如何影响学习投入。

Methods采用心理弹性量表、学业自我效能感量表、大学生学习投入问卷、大学生专业承诺量表对中国河南省1032名大学生被试进行调查，使用SPSS及其插件process对数据进行中介调节作用分析。

Results结果表明学业自我效能感与大学生学习投入显著正相关，学业自我效能感对学习投入的正向预测通过心理弹性和专业承诺的完全并行中介作用发挥效应，且专业承诺的中介效应大于心理弹性的中介效应；进一步的研究发现专业承诺的中介作用受到性别的调节，对专业承诺感知水平较强的女生有更高的学习投入水平。性别对心理弹性的调节作用并不显著。

Conclusions为提高大学生的学习投入水平，必须关注他们的学业自我效能感、专业承诺水平和心理弹性。

关键词：学习投入，学业自我效能感、专业承诺、心理弹性

引言

学习者的学习投入水平是教育者关注的问题[1]，后疫情时代如何采取有效的措施来提高大学生的学习投入度，是许多国家关注的话题。越来越多的研究表明有效的学习取决于学习者本人[2]。有效学习的开展需要学生积极参与学习，并把学习到的知识进行内化，形成自己的学习经验[3]。学习投入是影响学生学业成绩的重要因素[4]。越来越多的国家把学习的参与度与学习成绩、奖惩制度、辍学率、毕业率联系起来。

学习投入是预测学习质量的重要因素[5]。疫情大爆发时期，大学生普遍居家通过网络进行学习，学习模式较疫情发生前发生重大改变，由传统的面对面的集体学习变成非接触式的独自学习。随着疫情的结束，大学生重返教室开始面对面的学习，但受疫情时期学习模式的影响，他们学习主动性不高，学习投入水平低。因此有必要探讨后疫情时代如何提高大学生的学习投入水平。

1.学习投入（Learning engagement）

学习投入是指学生在学习过程中投入的时间、精力及由此引发的持久充沛的情感状态及认知状态[6，7]。有一些理论可以解释学习投入的过程，如社会认知理论和自我决定理论。与本文相关的是社会认知理论，该理论认为个体行为会受到社会环境因素与个人因素的影响。自我效能感是社会认知理论中的一个重要理论。班杜拉认为自我效能感受到所处环境的影响，一方面会影响认知过程，高的自我效能会促进个体认知发展进而促进学业行为，另一方面会对个体的行为产生影响[8]。高自我效能感的个体会选择富有挑战的学业任务，并为之付出更多的努力，即使遇到重大挫折，也能快速恢复，继续向目标努力。已有研究验证了社会认知理论，学习投入与心理弹性[9-12]、感知学习无效[13]关系密切。

学业自我效能感与学习投入的关系

自我效能感指个体对自身组织和执行某一任务的信心和感受[14,15]。自我效能感最早由班杜拉提出，是一种重要的心理因素，强烈地影响着人们的行为和表现。自我效能感包括效能预期和结果预期两个组成部分[16]。一般自我效能感是广义的概念，在这一概念被提出后，各个领域开展了广泛的研究，陆续提出了学业效能感、组织效能感等衍生概念。

学业自我效能感即在学习方面的自我效能感，指学习者对自己学习能力的评估，是学习者对自己组织和执行某一学习任务，成功理解学习材料的信心和感受[15]。作为学习的一个重要预测变量，它强烈地影响着学生的学习行为和学习表现，在很大程度上会影响学生学习的投入程度。已有众多研究表明大学生学业自我效能感与学习投入之间存在正相关关系。学业自我效能感强的学生，对自己完成学习任务的信心更强，对学业的投入度会更高。相反，学业自我效能感较低的学生，在学习中会体会到更多的无助感，体验到的消极情绪也更多，学习的参与度会降低[17]。学业自我效能感激励学习者选择适合自己的方式实现目标，对完成学习任务有很大的影响。学业自我效能感强的人对学习过程有良好的认知，其对没有成功的归因多是缺乏努力，而不是能力。学生的学业自我效能感与学习投入之间关系密切[18]。基于此，提出假设1：学业自我效能感能正向预测学习投入。

3.心理弹性在学业自我效能感与学习投入之间的中介作用

（Psychological resilience）

心理弹性是个体在面临困难时增强自身容纳力和有效应对压力源的重要能力[19,20]。心理弹性被定义为个体在面临逆境、创伤、不幸和重大压力源时，保持良好的适应状态或从逆境中“反弹”恢复正常生活的能力[21]。心理弹性框架理论认为人们在应对应激时会产生三种适应结果：第一种是弹性水平更高；第二种是维持原有弹性水平；第三种是受应激惊吓后弹性水平变低。之所以产生不同的适应结果，由所处环境、个体因素以及个人与环境交互作用影响[22]。该理论认为心理弹性具有动态性和可塑性，是心理发展过程中的一个重要保护因素[23]。心理弹性不是人格的内在特征，它伴随着个体的整个生命历程不断发展，受到所处生活环境的影响[24,25]。Leontopoulou[26]的研究发现即使在逆境中，积极的应对策略和回避的应对策略都会显著影响心理弹性。心理弹性良好的个体有很强的适应能力，吸收和利用应对策略的能力很强。在学习中拥有良好情绪的个体会运用各种有效的策略来提高自己的学习积极性及参与度。Alazemi et al.[27]通过对高中生的调查发现，学生的学业心理弹性水平越高，自我效能感越强，反之自我效能感越低。学业自我效能感作为自我效能感在学习方面的表现，其研究结果也说明自我效能感与心理弹性之间关系密切。

社会认知理论强调自我效能型的人对自己成功完成某项工作有更强的信念，设定的目标会更具挑战性，在遇到困难时能投入更多的精力和毅力来应对。Wicaksono et al.[28]在研究第二语言学习时发现，自我效能感、毅力、学业弹性与学习动力不足（academic demotivation）之间的密切相关。自我效能感和毅力能使学习者在第二语言的学习过程中体验到更多的对学习结果的良好预期，增强学业弹性，进而长久地保持更加高效的学习投入。Shao and Kang [29]等人的研究表明学业心理弹性、自我效能感和学习投入之间关系密切。具有学业心理弹性的学生尽管面临困境，但其往往对自己完成学习任务具有较强的信心，认为自己有能力完成此任务。因此，具有较高的学习投入水平。Rajan et al.[30]通过对印度155名高中生的调查发现，学业弹性在男女生之间存在显著差异，高中生学业弹性与自我效能感存在正相关关系。基于以上研究，心理弹性、学业自我效能感和学习投入之间关系密切。然而三者之间的潜在机制仍然需要澄清，对此本研究提出假设2：心理弹性在学业自我效能感和学习投入之间起中介作用。

4.专业承诺（Professional commitment）在学业自我效能感与学习投入之间的中介作用

专业承诺反映个体对其所学专业的态度和行为，指个体认同所学专业并愿意在学习领域花费时间和精力[31]，是个体热爱和忠实于所学专业的表现。专业承诺是了解学生专业学习投入度的重要指标。以前的研究已经证实了专业承诺与学习投入之间有显著的相关关系。Chen[32]以750名学前教育专业大学生为被试，采用问卷法调查了他们学习满意度、专业承诺与学习投入的关系，结果表明学前教育大学生专业承诺处于中等水平，但是学习投入得分较高。学习投入与专业承诺之间存在显著的正相关关系。

有研究表明自我效能感与专业承诺关系密切，尤其是与情感承诺的关系密切。Tsai et al.[33]的研究认为，良好的自我效能水平对情感承诺有积极的影响。这种积极效应的产生是因为自我效能感高的个体比自我效能感低的个体更倾向于接受组织中的目标和价值观。Orgambídez et al.[34]的研究验证了工作投入、自我效能感与情感性组织承诺之间关系密切。具有良好自我效能感的个体在情感上更能接受自己的工作单位，更愿意把更多的精力投入到工作中。基于此，提出假设3：专业承诺在学业自我效能感和学习投入中起中介作用。

5. 性别在专业承诺和学习投入之间的调节作用

性别是影响学习投入的重要人口学变量。由于男女大脑认知结构的不同导致男女生在认知投入的策略偏好不同。相比女生来说，男生的信息加工能力更强，元认知监控、调节策略更好。女生更关注外在的学习辅助手段及认知策略的学习[35]。其次，影响学习投入的因素也存在性别差异。性别分化理论指出，伴随着性别的生理分化，个体在社会建构的过程中逐渐形成性别角色观念。这个过程意味着个体的不断成长和社会化进程的推进。不同性别的个体会以自己对专业期待的方式参与专业学习，并根据自己通过学习后形成的对所学专业的理解不断调整对所学专业的期待。由此建构的专业承诺水平也不一样，对学习投入的水平也不一样[32]。例如，男生对培养动手操作能力、问题解决能力的专业更加适应，因而其在这类专业中的主动学习行为、互动学习行为更多；女生更加偏爱培养阅读能力、思辨能力的专业，其在这类专业中的学习投入度会更高一些。由此推断，专业承诺对大学生学习投入行为的影响可能存在性别差异。基于此，提出假设4：性别能够调节专业承诺与学习投入间的关系。

综上所述，本研究综合社会认知理论，试图构建一个有调节的并行中介模型，探讨大学生的学业自我效能感如何通过心理弹性和专业承诺的并行中介作用影响学习投入行为以及性别在其中的调节作用，以期为提高大学生学习参与度提供参考。

图1：模型图

程序

被试

使用整群随机抽样的方式招募来自中国河南一所高校大一至大四本科生参加研究。我们使用问卷星匿名收集材料，共收到问卷1187份，剔除漏题、不认真作答等无效问卷后，最终得到有效问卷1032份。参与者包括大一376人，占36.4%；大二273人，占26.5%；大三263人，占25.5%；大四120人，占11.6%。参与者中男生479人，占46.4%；女生553人，占53.6%。学生干部220人，占21.3%；非学生干部812人，占78.7%。独生子女148人，占14.3%；非独生子女884人，占85.7%。自主选择高考专业志愿的732人，占70.9%；遵从父母和他人意愿的107人，占10.4%；调剂安排专业的193人，占18.7%。

该研究于2023年3月至9月进行。研究获得了黄淮学院学术委员会的批准，通过一个名为“问卷星”的在线问卷平台进行测试，参与者以班级为单位集体施测。施测前获得了所有被试的知情同意。

**使用问卷**

心理弹性

本研究中采用中国学者 Yu 和 Zhang 翻译并修订的中文版心理弹性量表（CD-RISC）[36]。CD-RISC由美国心理学家Connor 和 Davidson 于 2003年编制，包括自强、坚韧、乐观3个维度，总共25道题目。采用 Likert 5 级评分法，从“从不”到“几乎总是”分别记0-4分，总分为 (0-100)分。得分越高，说明心理弹性水平越好。中文版 CD-RISC 量表 Cronbach's α 系数为0.916。本研究中Cronbach's α 系数为0.963。

学业自我效能感

本研究采用梁宇颂（2004）编制的《学业自我效能感量表》[37]。该量表共22道题，包含学习能力自我效能感和学习行为自我效能感2个维度。该量表采用5级评分标准，从“非常不同意”到“非常同意”分别记1-5分。其中14,16,17,20题采用反向计分，其他题目均为正向计分，问卷得分越高说明学生的学业自我效能感越高。本研究中Cronbach's α 系数为0.915。

学习投入

大学生学习投入问卷用来评估学习投入程度[38]，问卷共20个题目，包括行为投入、认知投入、情绪投入三个维度。采用五点计分。得分越高，表明被试的学习投入度越高。大学生学习投入问卷及行为投入、认知投入、情绪投入3个分问卷的Cronbach's α系数分别为0.918、0.825、0.858、0.858。该量表的Cronbach's α系数为0.918，表明该量表的信度较好。本研究中Cronbach's α 系数为0.969。

专业承诺

采用连榕等编制的《大学生专业承诺量表》[39]。该量表共 27 道题，并由情感承诺、继续承诺、规范承诺、理想承诺等维度组成。采用李克特 5 点计分法计算得分，从“完全不符合”到“完全符合”分别记1-5分。其中第 6、8、12 为反向计分题。被调查者得分越高，表明其专业承诺水平越高。根据连榕等人的研究，该量表测量的内部一致性 Cronbach α 系数为 0.92。本研究中Cronbach's α 系数为0.955。

共同方法偏差检验

本研究数据均来自于被试自我报告，可能导致共同方法偏差，因此采用 Harman 单因子检验法对其进行检验。结果显示，特征值大于 1的因子共11个，第一个因子解释变异量为 29.766% ，小于40% 的临界标准[40] 。因此，本研究不存在严重的共同方法偏差。

相关分析

**表 1 各变量的描述统计和相关分析（n=1156）**

| 变量 | 1 | 2 | 3 | 4 | 5 | 6 |
| --- | --- | --- | --- | --- | --- | --- |
| 1年级  2性别  3学业自我效能感  4心理弹性  5专业承诺  6.学习投入  平均值  标准差 | 1  -0.002  0.101^**^  0.073^*^  0.003  0.056  2.12  1.04 | 1  -0.039  0.015  -0.038  -0.065^*^  1.54  0.50 | 1  0.340^**^  0.227^**^  0.577^**^  3.42  0.50 | 1  0.370^**^  0.352^**^  3.45  0.64 | 1  0.320^**^  3.65  0.68 | 1  3.55  0.55 |

表1的结果表明学业自我效能感与心理弹性、专业承诺、学习投入之间呈正相关，心理弹性与专业承诺、学习投入之间呈正相关，专业承诺与学习投入呈正相关。

并行中介作用检验

研究采用Hayes [41] 编制的 SPSS 宏程序process 中的模型4 对心理弹性、专业承诺在学业自我效能感和学习投入之间的中介效应进行检验。结果如表2，图2所示：在控制了性别、年级、是否班干、是否独生、生源地等变量的条件下，学业自我效能感可正向预测心理弹性(β= 0.436，p＜0.001)和专业承诺(β= 0.640，p＜0.001)，心理弹性、专业承诺可预测正向学习投入(β= 0.312，p＜0.001；β= 0.263，p＜0.001)。而学业自我效能感对学习投入的预测作用变得不显著(β=0.001，p=0.976)。这表明学业自我效能感对学习投入的直接作用不明显，专业承诺和心理弹性在学业自我效能感和学习投入之间起完全中介的作用。

0.645^＊＊＊^

-0.217^＊＊^

0.264^＊＊＊^

0.004

0.340^＊＊＊^

0.313^＊＊＊^

**图2：完全中介路径图**

**表2心理弹性和专业承诺的中介效应检验**

中介路径	效应值	95%CI	效应量

间接效应	心理弹性	0.136	[0.094 0.183]	44.30%

专业承诺	0.170	[0.104 0.234]	55.37%

总间接效应	0.307	[0.232 0.379]	99.67%

中介效应的分析结果表明，在学业自我效能感→心理弹性→学习投入的中介效应值为0.136，在学业自我效能感→专业承诺→学习投入的中介效应值为0.170。效应值95％的置信区间没有包括０，说明心理弹性和专业承诺在大学生学业自我效能感与学习投入之间的中介效应均显著，心理弹性与专业承诺在大学生学业自我效能感对学习投入的影响中起到完全中介作用。

性别的调节作用

回归方程	整体拟合指数 回归系数及显著性

结果变量	预测变量	R	R^2^	F	β	t

学习投入	0.341	0.117	16.877^＊＊＊^

性别	-0.035	-0.859

生源地	0.062	1.287

是否独生	0.107	1.739

志愿选择	0.000	0.003

是否学生干部	-0.022	-0.449

性别×专业承诺	-0.217	-2.999^＊＊^

**表3：有调节中介效应结果**

为进一步探讨专业承诺存在性别差异的原因，使用模型14检验性别在原并行中介模型间的调节作用。结果如表3所示，原有路径的显著性与先前保持一致，性别对专业承诺中介后半段(β=-0.217，p＜0.01)的调节作用均显著，对专业承诺的前半段调节作用不显著，性别对心理弹性中介前后半段的调节作用均不显著。

图2：性别在学习投入与专业承诺中的中介作用

为进一步探究专业承诺和性别对学习投入的调节作用，以专业承诺得分的上下一个标准差为原则，将专业承诺分成高专业承诺组和低专业承诺组对结果进行简单斜率分析。简单斜率图见图2。

对女生来说，专业承诺对学习投入的影响呈上升趋势，专业承诺对学习投入的正向预测作用显著（β=0.445，t=8.107，p＜0.001) 。而对于男性来说，专业承诺对学习投入的预测作用仍然显著。（β= 0.228，t=3.843，p＜0.001)。

讨论

本研究结合社会认知理论和心理弹性框架理论，探究了学业自我效能感对学习投入行为的影响，揭示了学业自我效能感通过心理弹性和专业承诺对学习投入的影响机制以及性别差异，对提高学生的学习投入度具有一定的理论和实践价值。

学业自我效能感和学习投入呈正相关,假设1得到证实。学业自我效能感强的学生能更有效地处理教育生活中遇到的学业困难。而学业自我效能感弱的学生更容易产生自我怀疑，不愿意执行所面临的学习任务，以此避免学业失败[42]。马斯洛的需要层次理论提出每个人都有七种层次的需要，从低到高依次为生理需要、安全需要、归属与爱的需要、尊重的需要、认知需要、审美需要、自我实现的需要。马斯洛认为一般低层次需要得到满足，才有可能达到自我实现。该理论认为学生学习动机不强可能是某些层次的需要没有被满足。当学生在学习中对学习结果有很好预期且觉得自己有能力完成学习任务时，尊重的需要和认知的需要会变得异常强烈。这些需要一旦被满足，其会产生更高层次的求知需要，学生会继续选择有挑战性的任务，自愿投入更多的资源到学习过程中，展现出更高的学习参与水平。相反，当学生对学习的结果产生不良的预期，且对自己的能力产生怀疑时，一方面学生会担心成绩差而被老师和同学排斥，不在学习中投入过多精力，进而可能出现学习倦怠，甚至出现逃学行为。另一方面，学生对自己的学习能力产生怀疑，可能会逐渐产生习得性无助，产生自卑心理。一般情况下归属与爱的需要及自尊的需要得不到满足，求知的需要动力就会变弱。

研究发现学业自我效能感通过心理弹性影响学业投入,假设2得到证实。个体的注意、认知、情绪、行为等因素会影响心理弹性的发展。心理弹性框架理论认为，个体之所以出现不同的适应结果，受到环境、个体内部因素以及个体与环境相互作用三个方面的内容共同决定。认知、情绪、身体、精神、行为五个方面构成心理弹性的个人因素。积极情绪能扩展个体注意和认知的范围，还能不断建构个人的积极资源，提高行为的积极性[43]。本研究支持了这一理论，证明了心理弹性水平高的个体，情绪会更加积极、态度会更加乐观，在面对学习任务时会更加相信自己的能力并采取更加积极的行动，这种积极主动的行为会使个体在学习过程中投入更多的精力。心理弹性源于特定的信念系统，该系统包括一个人对自己、对周围人、对世界真善美的看法。信念系统会受到个人所处人生阶段的各种因素影响[44]。

本研究的结果显示专业承诺在学业自我效能感和学习投入之间起中介作用，,假设3得到证实，这与先前的研究的研究结果一致。Zhou and Wu[45]的研究表明，专业承诺、学业自我效能感、教师支持和学习投入之间两两相关。专业承诺、学业自我效能感显著正向预测学习投入。Lu et al.[46]通过对400余名医学生的专业承诺调查研究发现，自我效能感通过专业承诺和学习投入影响学业成绩。也就是说那些对自己学习能力有很好评估的学生往往会对自己所学的专业表现出强烈的热爱情感，并对自己所学专业的发展抱有较高的期望，愿意遵守所学专业的规范和要求，相信能以自己的能力去克服学习中所面临的内在和外在挑战，并在实际学习中不断体验和验证自己的想法，对专业学习的投入精力也会越来越多。

本研究发现学业自我效能感通过专业承诺影响学习投入的中介作用的后半段受到性别的调节，假设4得到证实。性别差异检验发现专业承诺可以显著影响男女大学生的学习投入，并且对女生的影响更大。这可能一方面与性别角色的传统定位或社会期望密切相关。在社会化的过程中，个体习得性别认知图式，性别图式会使个体表现出明显的性别倾向[47]。女生更为感性、顺从性较强，在情感上更为认可自己所学专业，会不自觉地对所学专业产生理想化的憧憬，愿意把更多的精力投入到学习中去。相比而言，男生会更为理性，思想上求新求异，控制感较强，对所学专业的评判更为综合和客观。学生容易受人们这种根深蒂固的性别角色认知与期待的潜移默化地影响，从而对专业的认同水平产生性别上的差异。因此，即使拥有相同的专业认同水平，女性的学习投入度更高一些。

结论

本研究构建了一个有调节的中介模型来解释学业自我效能感与学习投入之间的关系。结果发现学业自我效能感能够显著正向预测大学生学习投入行为，并且心理弹性和专业承诺在学业自我效能感和学习投入之间起并行中介作用，专业承诺的中介作用大于心理弹性的正向中介作用；学业自我效能感对大学生学习投入行为的预测作用完全以心理弹性和专业承诺为中介。研究还发现，性别调节专业承诺的后半段路径。具体而言，女性的专业承诺比男性更强，比男性有更高的学习投入。

研究价值和不足

研究价值

本研究对后疫情时代如何提高学习投入进行研究具有一定的理论意义和实践意义。首先，本研究尝试构建学业自我效能感通过心理弹性和专业承诺影响学习投入的中介模型，对社会认知理论中自我效能感对学习投入的相关影响因素进行了补充，扩充了学业自我效能感影响学习投入的作用路径，为进一步深入理解学业自我效能感影响学习投入的作用机制提供了理论基础。其次，本研究对提高大学生学习投入具有重要的实践意义。后疫情时代，线上线下混合教学成为一种趋势，而学习投入是影响线上学习质量的关键因素。因此，有效提升学生的学习投入水平变得尤为重要。依据本研究的结果，可以从提高大学生心理弹性及专业承诺的水平两方面入手进行干预。根据专业承诺正向效应，一方面提倡学生在填写大学入学志愿时，充分考虑自己的个性特征、职业倾向，并详细了解所要填写专业的学习内容以及未来的就业方向、就业前景，以此提高对所学专业的情感满意度。另一方面，在一年级入学一段时间后，允许不能适应所学专业的学生对其专业进行调整。学校相关部门可以协助学生进行职业能力测试，帮助其选择更为合适的专业。根据心理弹性的正向效应，为提高大学生学习投入水平，在课堂及日常活动中增加挫折教育及心理弹性等积极心理学内容，以提高学生的心理弹性水平。特别是，针对因COVID-19造成心理创伤和学习困难的学生，有针对性地开展心理咨询、心理团体辅导、心理治疗等活动，帮助他们尽快恢复原有的心理弹性水平。

**不足**

本研究存在一些不足，首先，研究数据采用自我报告的形式，存在难以消除的共同偏差。其次，本研究采用横断面设计，无法深入考虑变量间的因果关系，未来可采用实验设计和纵向研究的方法进一步验证变量之间的因果关系。再次，本研究仅考察了专业承诺和心理弹性在学业自我效能感与学习投入之间的作用，未来应考虑更多的可能具有中介或调节作用的变量，如父母教养水平，同辈支持，未来取向等。

参考文献

1.Zheng C. Student Engagement and Academic Performance during the COVID-19 Pandemic: Does a Blended Learning Approach Matter?
International Journal for the Scholarship of Teaching and Learning*.*2023;17(1):1–9. https://doi.org/10.20429/ijsotl.2023.17107

2.Kumar S,Todd G.Effectiveness of online learning interventions on student engagement and academic performance amongst first-year students in allied health disciplines: A systematic review of the literature. Focus Health Prof Ed.2022;23(3);36–55. https://doi.org/10.11157/fohpe.v23i3.430

3.Rashid T, Asghar HM. Technology use, self-directed learning, student engagement and academic performance: Examining the interrelations. Comput Hum Behav*.*2016;63; 604–612. https://doi.org/10.1016/j.chb.2016.05.084

4.Sahni J. Is Learning Analytics the Future of Online Education?: Assessing Student Engagement and Academic Performance in the Online Learning Environment.
Int J Emerg Technol*.* 2023;18(2);33–49. https://doi.org/10.3991/ijet.v18i02.32167

5.Bayoumy HMM, Alsayed S. Investigating Relationship of Perceived Learning Engagement, Motivation, and Academic Performance Among Nursing Students: A Multisite Study. Adv Med Educ Pract.2021;12;351–369. https://doi.org/10.2147/AMEP.S272745

6.Fredricks JA, Blumenfeld PC, Paris AH. School Engagement: Potential of the Concept, State of the Evidence. Rev Educ Res*.* 2004;74; 59–109. https://doi.org/10.3102/00346543074001059

7.Schaufeli WB, Martínez IM, Pinto AM, Salanova M, Bakker AB.Burnout and Engagement in University Students: A Cross-National Study. J Cross Cult Psychol*.*2002; 33(5);464–481. https://doi.org/10.1177/0022022102033005003

8.Bandura A. On the Functional Properties of Perceived Self-Efficacy Revisited. J Manage. 2012;38(1);9–44. https://doi.org/10.1177/0149206311410606

9.Hartley MT.Examining the Relationships Between Resilience, Mental Health, and Academic Persistence in Undergraduate College Students. J Am Coll Health*.* 2011;59(7); 596–604. https://doi.org/10.1080/07448481.2010.515632

10.Smith BW, Dalen J, Wiggins K, Tooley E, Christopher P, Bernard J.The brief resilience scale: Assessing the ability to bounce back. *International Journal of Behavioral Medicine.* 2008 ; 15(3);194–200. https://doi.org/10.1080/10705500802222972

11..Zeng G, Hou H, Peng K.Effect of Growth Mindset on School Engagement and Psychological Well-Being of Chinese Primary and Middle School Students: The Mediating Role of Resilience.Front Psychol. 2016;7;1664-1078. https://doi.org/10.3389/fpsyg.2016.01873

12.Zhao H, Xiong J, Zhang Z, Qi C.Growth mindset and college Students' learning engagement during the COVID-19 pandemic: A serial mediation model. Front Psychol. 2021;12; 1664-1078.

https:// doi.org/10.3389/fpsyg.2021.621094

13. Ye JR, Wu YF, Nong W,Wu YT, Ye JN, Sun Y. The Association of Short-Video Problematic Use, Learning Engagement, and Perceived Learning Ineffectiveness among Chinese Vocational Students. Healthcare ;2023;11(2);161. https://doi.org/10.3390/healthcare11020161

14.Bandura A. Social Foundations of Thought and Action: A Social Cognitive Theory.Upper Saddle River;Prentice-Hall Press;1986 .

15.Bandura A.Self-Efficacy: The Exercise of Control.New York；W.H. Freeman & Company；1997.

16.You W.Research on the Relationship between Learning Engagement and Learning Completion of Online Learning Students. Int J Emerg Technol.2022; 17(1);102–117. https://doi.org/10.3991/ijet.v17i01.28545

17.Namaziandost E, Heydarnejad T, Saeedian S.Language Teacher Professional Identity: The Mediator Role of L2 Grit, Critical Thinking, Resilience, and Self-efficacy Beliefs . Iranian Journal of Applied Language Studies*.*2023; 14(2);107-130. https://doi.org/10.22111/IJALS.2022.7486

18.Xie D, Xie Z. Effects of Undergraduates’ Academic Self-Efficacy on Their Academic Help-Seeking Behaviors: The Mediating Effect of Professional Commitment and the Moderating Effect of Gender.J Coll Student Dev. 2019;60(3); 365–371.https://doi.org/10.1353/csd.2019.0035

19.Ahern NR,Norris AE .Examining factors that increase and decrease stress in adolescent community college students.J Pediatr Nurs.2011;.26(6);530-540.https://doi.org/530-540.10.1016/j.pedn.2010.07.011

20.Cooper AL, Brown JA, Rees CS, Leslie GD.Nurse resilience: A concept analysis. Int J Ment Health Nu , 2020; 29(4);553-575.https://doi.org/10.1111/inm.12721

21.Kumpfer KL.Factors and Processes Contributing to Resilience.2002 ; in: Glantz MD., Johnson JL. Resilience and Development, Longitudinal Research in the Social and Behavioral Sciences: An Interdisciplinary Series. Kluwer Academic Publishers, Boston, 179–224. https://doi.org/10.1007/0-306-47167-1_9

22.Luthar SS, Cicchetti D, Becker B. The construct of resilience: a critical evaluation and guidelines for future work. Child Dev,2000;71(3);543–562. <https://doi.org/10.1111/1467-8624.00164>

23.Cheung VHM, Chan CY, Au, RKC. The influence of resilience and coping strategies on suicidal ideation among Chinese undergraduate freshmen in Hong Kong. Asia-Pac Psychiat .2019;11(2);1758-5864. https://doi.org/10.1111/appy.12339

24.Gillespie BM, Chaboyer W, Wallis M. Development of a theoretically derived model of resilience through concept analysis*.*Contemp Nurse.2007;25(1-2);124-135https://doi.org/10.5172/conu.2007.25.1-2.124

25.Celik DA, Cetin F, Tutkun E. The role of proximal and distal resilience factors and locus of control in understanding hpe, self-esteem and academic achievement among Turkish pre-adolescents.Curr Psychol. 2015;34(2); 321- 345.

26.Leontopoulou.S. Resilience of Greek Youth at an Educational Transition Point: The Role of Locus of Control and Coping Strategies as Resources.Soc Indic Res .2006;76(1); 95–126. https://doi.org/10.1007/s11205-005-4858-3

27.
